# The patient advisor, an organizational resource as a lever for an enhanced oncology patient experience (PAROLE-onco): a longitudinal multiple case study protocol

**DOI:** 10.1186/s12913-020-06009-4

**Published:** 2021-01-04

**Authors:** M. P. Pomey, M. de Guise, M. Desforges, K. Bouchard, C. Vialaron, L. Normandin, M. Iliescu-Nelea, I. Fortin, I. Ganache, C. Régis, Z. Rosberger, D. Charpentier, L. Bélanger, M. Dorval, D. P. Ghadiri, M. Lavoie-Tremblay, A. Boivin, J. F. Pelletier, N. Fernandez, A. M. Danino

**Affiliations:** 1 Centre de recherche du Centre Hospitalier de l’Université de Montréal (CR-CHUM), 850, rue Saint-Denis, Montréal, Québec H2X 0A9 Canada; 2 Centre d’Excellence pour le Partenariat avec les Patients et le Public, 900, rue Saint-Denis, Porte S03.900, Montréal, Québec H2X 0A9 Canada; 3 École de santé publique de l’université de Montréal-Département de gestion, évaluation et politique de santé, 7101 Av du Parc, Montréal, Québec H3N 1X9 Canada; 4 Université de Montréal – Faculté de Médecine, 2900 boulevard Edouard-Montpetit, Montréal, Québec H3T 1J4 Canada; 5 Institut national d’excellence en santé et services sociaux (INESSS), 2021, avenue Union, 12e étage, bureau 1200, Montréal, Québec H3A 2S9 Canada; 6 Centre Intégré Universitaire de santé et services sociaux de l’Est-de-l’Île-de Montréal, Hôpital de Maisonneuve-Rosemont, 5415, boulevard de l’Assomption, Montréal, Québec H1T 2M4 Canada; 7 CHU de Québec-Université Laval, 10, Rue de l’Espinay, Québec, Québec G1L 3L5 Canada; 8 Université de Montréal – Faculté de Droit, 3101 chemin de la Tour, Montréal, Québec H3T 1J7 Canada; 9 Lady Davis Institute for Medical Research, Jewish General Hospital & McGill University, Gerald Bronfman Department of Oncology, 5100 de Maisonneuve Blvd West, Montréal, Québec H4A 3T2 Canada; 10 Centre Hospitalier Universitaire de Montréal (CHUM), 1000 rue Saint-Denis, Montréal, Québec H2X 0C1 Canada; 11 Université Laval – Faculté de pharmacie, 050, avenue de la Médecine, Québec, Québec G1V 0A6 Canada; 12 Centre de recherche du CHU de Québec-Université Laval, 1050 chemin Sainte-Foy, Québec, Québec G1S4L8 Canada; 13 Centre de recherche du CISSS Chaudière Appalaches, 143 rue Wolfe, Lévis, Québec G6V 3Z1 Canada; 14 HEC Montréal, Department of management, 3000, chemin de la Côte-Sainte-Catherine, Montréal, Québec H3T 2A7 Canada; 15 McGill University, Ingram School of Nursing (IsoN), 680 Sherbrooke Street West, Montreal, Québec H3A 2M7 Canada; 16 Centre Universitaire de Santé McGill (CUSM), 1650, avenue Cedar, Montréal, Québec H3G 1A4 Canada; 17 Centre de Recherche de l’Institut universitaire en santé mentale de Montréal, 7331 Rue Hochelaga, Montréal, Québec H1N 3V2 Canada

**Keywords:** Patient advisor, Oncology, Co-construction, Patient care experience, Patient partnership, Longitudinal case study

## Abstract

**Background:**

Quebec is one of the Canadian provinces with the highest rates of cancer incidence and prevalence. A study by the Rossy Cancer Network (RCN) of McGill university assessed six aspects of the patient experience among cancer patients and found that emotional support is the aspect most lacking. To improve this support, trained patient advisors (PAs) can be included as full-fledged members of the healthcare team, given that PA can rely on their knowledge with experiencing the disease and from using health and social care services to accompany cancer patients, they could help to round out the health and social care services offer in oncology. However, the feasibility of integrating PAs in clinical oncology teams has not been studied. In this multisite study, we will explore how to integrate PAs in clinical oncology teams and, under what conditions this can be successfully done. We aim to better understand effects of this PA intervention on patients, on the PAs themselves, the health and social care team, the administrators, and on the organization of services and to identify associated ethical and legal issues.

**Methods/design:**

We will conduct six mixed methods longitudinal case studies. Qualitative data will be used to study the integration of the PAs into clinical oncology teams and to identify the factors that are facilitators and inhibitors of the process, the associated ethical and legal issues, and the challenges that the PAs experience. Quantitative data will be used to assess effects on patients, PAs and team members, if any, of the PA intervention. The results will be used to support oncology programs in the integration of PAs into their healthcare teams and to design a future randomized pragmatic trial to evaluate the impact of PAs as full-fledged members of clinical oncology teams on cancer patients’ experience of emotional support throughout their care trajectory.

**Discussion:**

This study will be the first to integrate PAs as full-fledged members of the clinical oncology team and to assess possible clinical and organizational level effects. Given the unique role of PAs, this study will complement the body of research on peer support and patient navigation. An additional innovative aspect of this study will be consideration of the ethical and legal issues at stake and how to address them in the health care organizations.

**Supplementary Information:**

The online version contains supplementary material available at 10.1186/s12913-020-06009-4.

## Background

Quebec is one of the provinces in Canada with the highest rates of cancer incidence and prevalence. In 2019, 55,600 Quebecers received a cancer diagnosis [1]. Given that prevention and treatment of cancer are public health priorities in Quebec, the provincial ministry of health and social services has developed a strategic plan for the fight against cancer, namely the Quebec cancerology Program (PQC). The PQC includes a framework and an action plan to reduce the incidence and prevalence of cancer, as well as to improve the quality (accessibility, effectiveness, efficiency, relevance, person-centered care), safety (treatment errors, health care acquired infections), and experience of care and services for cancer patients [2, 3]. One way to help improve the experience of care and services is through Patient-provider partnerships which go beyond patient-centered care and include: 1) recognition of patients’ experiential knowledge; 2) survivors’ status as full-fledged members of the care and services team; 3) patients’ role as one’s own care giver; and 4) patients’ capacity for self-determination for making decisions concerning themselves according to their needs and values [4]. One application of the patient-provider partnership approaches to engage patients who have experienced a health problem and the health care system to help those with similar problems get through their experience or to participate in committees to improve the quality and safety of care. Patients are already well integrated in oncology governance committees in Quebec [5], but the integration of patient advisors in care and services teams has not been fully achieved.

Some studies show the potential of including patient advisors in clinical oncology teams to facilitate communication between patients and their health and social care team, and to improve the experience of care for patients with cancer as well as those at high risk for developing this disease, particularly in terms of information sharing, emotional support [6–12], and engagement in their care [13]. PA interventions are even more relevant since emotional support is an unmet need of people who have to deal with cancer [14].

Kowitt and collaborators’ 2019 study reveals that peer support is very common in all phases of the oncology continuum of care [9]. This support is usually provided by “Patient Navigator” programs where the patient navigator’s main role is to help patients access care; thereby, reducing the time to receive a diagnosis and treatment and reducing the number of patients lost to follow up [15–18]. Moreover, these navigators can include nurses, social workers, educators, as well as former patients [19]. These programs [19] have been shown to improve provide comfort to patients [6], increase adherence to treatment [20], improve their health [21, 22], help them find their way in the healthcare system [7], reduce wait times [20], and lower hospital readmission rates [23]. Such programs tend to focus on underprivileged groups, rather than targeting all cancer patients [24].

In oncology, patient advisors (PA) programs are similar to patient navigator ones, but in addition they consider PAs as being integral members of the clinical team with the skills to help other patients navigate their health situation [25]. Thus, PAs play a distinct and innovative role comparable to that of peer helpers in mental health contexts [23, 26]. A study carried out at the Centre of Expertise for Traumatic Amputation Victims Requiring Emergency Microsurgical Re-implantation (CEVARMU) in Quebec showed that including PAs on the health and social care team had positive impacts on the patients, the PAs, the health care professionals, the administrators, and the decision-makers [27]. Among the main advantages observed were the reduction of depressive symptoms, the reduction of pain and anxiety, the improvement of quality of life, treatment compliance, and healthy lifestyle habits [28]. However, this study also highlighted ethical and legal issues related to the status of PA and the limits of their role. The feasibility, acceptability, impacts and ethical/legal issues of the integration of patient advisors in oncology remain to be explored and documented.

This action-research intervention [29], inspired by realistic evaluation methodology [30–32] aims to evaluate the feasibility of integrating PAs within health and social care oncology teams and will be conducted in close collaboration with the actors on the ground. The specific objectives are to: 1) describe the mechanisms of co-constructing the implementation processes of the PA program; 2) identify factors that facilitate and hinder the integration and participation of PAs in health and social care oncology teams; 3) assess the effects of the PA intervention on the patients, the PAs, the healthcare team, the administrators, and the clinical organization of care; 4) identify ethical and legal issues of integrating PA in healthcare settings.

## Conceptual framework

**Fig. 1:**
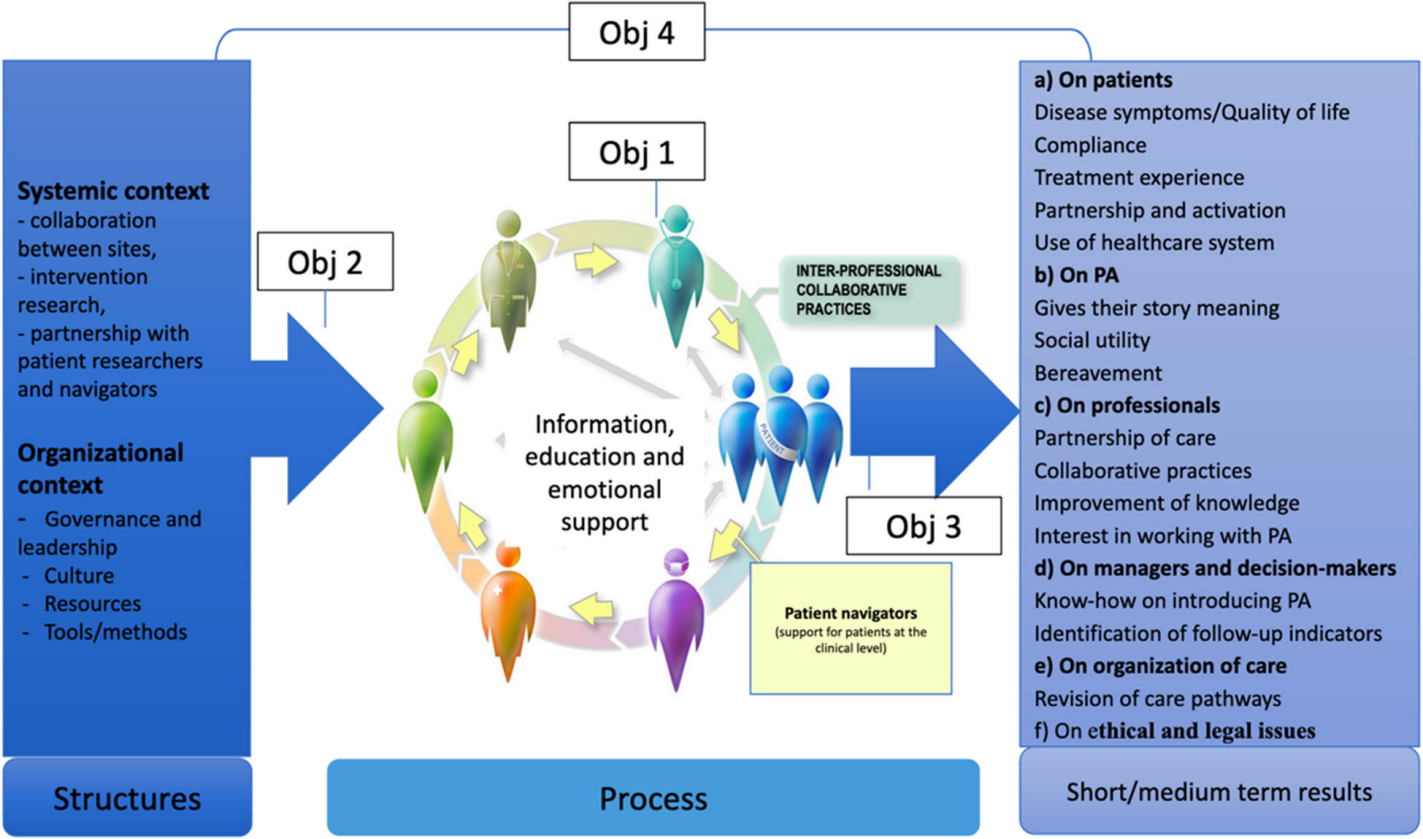
Conceptual framework and the 4 objectives

The conceptual framework that will guide this study is inspired by Donabedian [33], and highlights the importance of taking into consideration organizational factors that influence PA integration processes and effects, the processes of integration of PAs into clinical teams and the effects of this integration at the clinical and organizational levels (see Fig. 1). Specifically, the organizational factors that will be studied are: 1) the governance and leadership of those who make decisions and implement PAs [34, 35]; 2) the organizational culture, which includes beliefs, values, norms, and behaviors of those who work in the organization [36]; 3) the human, financial, structural, and information resources [37]; and 4) the methods and tools used to implement the PA intervention.

Regarding the integration processes, we are interested in how the PAs are integrated as full-fledged members of the clinical oncology teams and the nature of the meetings between patients and PAs. We will study the potential effects, if any, of the integration of PAs on the health and psychosocial care oncology teams on: 1) the patients (anxiety; depression; quality of life; compliance to treatments and to lifestyle changes; engagement in their care; experience of care; use of the healthcare system); 2) the healthcare teams (relationships with the patients, collaborative practices, knowledge of cancer patients’ needs, work satisfaction); 3) the administrators and decision makers (how to integrate PA into clinical oncology teams); 4) the PAs themselves (give their story meaning, feeling of usefulness); and 5) the clinical organizational of care .

## Methods/design

### Study design

The planned approach is a longitudinal multiple in-depth case study with two levels of analysis (clinical and organizational) that will be conducted over 4 years at six different sites. The longitudinal design is necessary to account for inevitable delays over the course of the implementation, to allow the time to consolidate changes made at each case site, and to allow the time needed to avoid making premature conclusions regarding the impacts of the changes [38, 39]. The multiple case study design allows for the in-depth analysis of the implementation processes in diverse oncology contexts. Qualitative and quantitative data will be used to document the intervention for each case, to examine the implementation processes, in their respective contexts, and to report their respective effects at the clinical and organizational levels [40]. This design is consistent with realistic evaluation [30] and intervention action-research [29] and structures our in-depth investigation of the links between the implementation and co-construction mechanisms and our ability to capture the challenges and the effects of the intervention.

### Settings

The settings for the cases are six health care organizations in different regions of Quebec: three university hospital centers (Centres Hospitaliers Universitaires) (CHU) and three regional health and social services authorities (Centres intégrés universitaires de santé et services sociaux) (CIUSSS). They were selected based on their interest in integrating PAs into a health and social care oncology team and their capacity to support care partnerships [41]. Specifically, in each setting the PA will be able to: 1) participate in therapeutic education activities about treatments and living with disease (education); 2) offer individualized support to oncology patients in conjunction with the clinical team (emotional); 3) help patients to be more involved in their care (e.g., participate in carrying out their personal action plan) (partnership). The patients selected to be PAs will be clinically stable, receive training (e.g., Knowing the limits of PA role, Communicating and listening, etc.), and will go to the care sites, several hours per week or per month to participate in the above-mentioned education, emotional, and partnership activities.

### Governance

Each site will assemble a local project committee made up of PAs, oncologists, administrators, nurses, oncology nurse coordinators, allied health professionals (e.g., social worker or psychologist), and a research assistant. An overarching study committee comprising the research coordinator for all sites, the research assistants of each site, and an administrator/clinician representative from each site will be in charge of setting up the PA program, discussing challenges and finding solutions, and facilitating the assessment. A community of practice of approximately 30 PAs (between 3 to 5 PAs will be recruited per site) will be formed so the PAs can share successes and challenges and learn from one another. Finally, a steering committee made up of the decision makers and researchers will meet annually to discuss the progress of the project, provide advice on how to carry out the research and comment on the results, and an advisory committee made up of the site representatives (the research assistant and the administrator or clinician) and researchers will review and interpret results of each phase of the study.

### Participants

For each setting, the representatives of high/mid-level managers working in the targeted program, the health and social care professional (HSP) team members, the patients, and the PAs (to be recruited) will be invited to participate in the project. For the patients, the inclusion criteria will be understanding written and spoken French, undergoing cancer screening or treatment, being at least 18 years old and have had at least one meeting with a patient advisor. All participants meeting the inclusion criteria will be invited to participate. This is therefore a consecutive sampling. Professionals and patient advisors will be approached by email. Patients will be approached by telephone or face-to-face through the patient advisor at their initial meeting. All participants will sign an informed consent form approved by Centre Hospitalier de l’Université de Montréal’s research ethics committee. Indeed, this study was submitted to and approved by the Centre Hospitalier de l’Université de Montréal’s research ethics committee.

### Qualitative data collection

Various qualitative data sources and methods will be used to study the co-construction mechanisms of the implementation of the PA program (obj 1), identify the enabling and inhibiting factors influencing PA integration (obj 2), assess the effects of the intervention (obj 3), and identify ethical and legal issues (obj 4). The triangulation of the data sources will contribute to the internal validity of the study [42].

#### Field documents

A document review will be conducted to study the evolution of the integration of the PAs in the health and social care oncology teams. It will include meeting minutes, action plans, emails, and other relevant documents produced during the project.

#### Interviews

At the beginning and the end of the study, semi-structured interviews will be conducted with approximately five stakeholders from each of the six settings among the directorates, the managers, the administrators/clinicians who will participate in the implementation of the PAs (total *n* = 60 (5x2x6)). As per qualitative research methodology, data collection and analysis will be concurrent, thus the actual number of interviews completed will be determined by data saturation, defined as no more new data/themes emerging from the interviews [43]. The interviews will focus on participants’ expectations of and experience with the implementation of the PA program, the facilitating and hindering factors, their perceptions of the stakes, and how to sustain the intervention. The interview guide will be developed and co-constructed with patients specifically for this study and will be pilot tested with a few people from the target population (see Additional file 1). Interviews will take place in the health care organization concerned or over the phone depending on the participant’s preference and be conducted by the research assistant. They will briefly describe their role in the project and their experiences with qualitative research to the participants. With the consent of the participants, an audio recording of the interview will be made, and field notes will be taken during the interview. They are scheduled to last 60 min.

#### Focus groups

In each setting, at the beginning and end of the project, two focus groups of eight to 12 participants among the representatives of the health and social care team, administrators, patients and PAs participating in the implementation of the PA program [44], will be conducted (total *n* = 12 (2 × 6)). The focus groups will address participants’ perceptions of their experience with the PAs, the interactions between the patients and the PAs and professionals, and the perceived effects of the program on the patients, the PAs and the health and social care oncology team. The focus group guide will be developed and co-constructed with patients specifically for this study and will be pilot tested with a few people from the target population (see Additional file 2). Focus groups will take place in the health care organization concerned and be conducted by the research assistant. They will briefly describe their role in the project and their experiences with qualitative research to the participants. With the consent of the participants, an audio and video recording of the focus group will be made, and field notes will be taken during the focus group. They are scheduled to last 90 min. However, the duration of the focus groups could be extended beyond the planned duration if new data/themes emerge in relation to the questions until saturation is reached.

#### Research logbook

A logbook will be used by the research assistant at each site to collect data regarding the context (the PA program chosen), the mechanisms of co-construction of the PA program (obj 1), the factors that facilitate and hinder the implementation (obj 2), the interactions among health and social care team (obj 3), PA and patients (knowledge exchange, tool used, evolution over time) (obj 3), the perceived influence of these dynamics on patients’ experience of care and quality of life (obj 3) and on the PAs and other health and social care team members experience of the PA program (obj 3), and the ethical and legal issues faced (PA status, PA training, PA integration into the clinical team, etc.) (obj 4).

### Qualitative data analyses

Conventional case study methods will be used to analyze the qualitative data [38]: the interviews and focus groups will be recorded and transcribed in full. Content analysis will be used to analyze these data, as well as the documents collected during the study [40] and will be structured in four phases: 1) creating a common codebook through independent near-data coding done by two researchers and discussion around inter-rater differences and using verbatim (about 10% of the verbatim); 2) coding the complete data corpus by one researcher using the codebook to systematize the description of each case; 3) identification of emerging common themes across cases to enrich the conceptual framework; 4) progressive construction of the cases through discussions with each site team. The analysis will be done with the help of the QDA Miner software (version 5.0) [45]. Validity will be ensured by triangulating data sources and this peer review publication of the protocol, peer debriefing during the analysis and case construction, confirmability will be enhanced by triangulation of the multiple data sources [46] and all data will be analyzed through our conceptual framework to help ensure the validity and the plausibility of findings. The COREQ checklist was used to report the methodology of the qualitative part (see Additional file 3).

### Quantitative data collection

Quantitative data collected will help to achieve the second and third objectives. Validated questionnaires will be used to collect data from each stakeholder group. A summary of the data collection timing and frequency is presented in Table 1. The secure online platform REDCAP (Research Electronic Data Capture), an application for building and managing online surveys and databases, will be used to administer the questionnaires, organize the data collection and analyze the data [52].

**Table 1:** List and description of quantitative data collection questionnaires

Outcomes	Questionnaires	Dimensions	Number of items	Response scale	Time of measurements	Question numbers in appendices
**Patient reported experience**	Patient’s care experience questionnaire ^a^	• Site of diagnosed cancer; cancer stage at time of diagnosis; type of treatment; date of start of treatments • Consultation in oncogenetics, genetic status, time of disclosure of results of genetic testing, surgery to reduce the risk of developing cancer, time of preventive surgery • Evaluation of information communicated by medical team, support of friends and family.	25 items	Varied	T0	Additional file 4: T0: 41 to 67
	Sociodemographic questionnaire ^a^	• Gender, age, country of origin, region, number of people in the household, number of dependents, family composition, level of education, perception of financial situation.	18 items	Varied	T0	Additional file 4: T0: 22 to 39
	Patient’s experience with PAs questionnaire ^a^	• Topics discussed, benefits and perceived contribution of PA • Expectations of patients that did not benefit from PA accompaniment	14 items	Varied	T0, T1, T2	Additional file 4: T0: 69 to 100 T1: same as T0 T2: 102 to 127
	Treatment adherence questionnaire [47]	• Intention of receiving treatments • Obstacles to treatment	8 items	5 point Likert scale (1 = strongly disagree and 5 = strongly agree)	T0, T1, T2	Additional file 4: T0: 148 to 159 T1: same as T0 T2: same as T0
	CASE [48]	**Ability to cope with cancer** • Understand and participate in care • Maintain positive attitude • Seek and obtain information	12 items	4 point Likert scale (1 = strongly disagree and 4 = strongly agree)	T0, T1, T2	Additional file 4: T0: 129 to 140 T1: same as T0 T2: same as T0
	Kessler Psychological Distress Scale (K6) [49]	• on emotional states	6 items	5 point Likert scale (1 = Always and 5 = never)	T0, T1, T2	Additional file 4: T0: 141 to 146 T1: same as T0 T2: same as T0
**PA reported experience**	PAs’ care experience questionnaire ^a^	• Site of diagnosed cancer; time of diagnostic; type of treatment; date of start of treatments • Consultation in oncogenetics, genetic status, time of disclosure of results of genetic testing, surgery to reduce the risk of developing cancer, time of preventive surgery • Evaluation of information communicated by medical team, support of friends and family.	25 items	Varied	T0	Additional file 5: T0: 39 to 60
	Sociodemographic questionnairev	• Gender, age, country of origin, region, number of people in the household, number of dependents, household situation, level of education, perception of financial situation.	18 items	Varied	T0	Additional file 5: T0: 19 to 36
	Previous personal experience with PAs questionnaire^a^	• Presence of pairing or not with a PA • Topics discussed, benefits and perceived contribution of PA • Expectations of patients that did not benefit from PA accompaniment are also evaluated	14 items	Varied	T0	Additional file 5: T0: 62 to 82
	Kessler Psychological Distress Scale (K6) [49]	• Psychological distress	6 items	5 point Likert scale (1 = Always and 5 = never)	T0, T2	Additional file 5: T0: 84 to 89 T2: idem to T0
	Review of PA experience with patient questionnaire^a^	• Support of clinical team • Quality of tools used • Useful information to effectively carry out their role as PA • Communication with the clinical team • Role in the clinical team • Perceived impact of their intervention on the patient and on the PA themself.	140 items	Varied	T2	Additional file 5: T2: 91 to 241
**Descriptive data of each meeting**	Logbook^a^	• PA name • Patient name • Date, time, duration • Context of the request • People present during the meeting • Context of the meeting • Step of patient trajectory • Topics discussed • Difficulties and questions • Feedback from the clinical team	28 items	Varied	After each meeting	Additional file 6: 3 to 42
**Health professional reported experience**	Adaptation of the Collaborative Practice Assessment Tool (CPAT) [50]	• Perceptions of concepts of collaborative practices, mission and goals of the teams (4 domains out of 8 domains retained and one created for the project) • General Relationships • Team leadership • General role responsibilities, autonomy • Communication and information exchange • Practice evaluation^a^	26 items retained out of 56	7 point Likert scale (1 = strongly disagree and 7 = strongly agree)	T0, T2	Additional file 7: T0: 22 to 47 T2: 103 to 128
	Group Innovation Inventory [51]	• Innovative culture (adaptation)	7 items retained out of 36	7 point Likert scale (1 = strongly disagree and 7 = strongly agree)	T0, T2	Additional file 7: T0: 48 to 54 T2: 129 to 135
	Readiness To Partner With Patient and Family Advisers - Patient and FamilyEngagement in the Surgical Environment Module. Content last reviewed May 2017. Agency for Healthcare Research and Quality,Rockville, MD. https://www.ahrq.gov/hai/tools/ambulatory-surgery/sections/implementation/training-tools/readiness.htm [50]	• Perception of health professionals concerning the partnership with patients (treatment plan, decision making) • Perception of health professionals concerning the role of PAs	11 items retained out of 18	5 point Likert scale (1 = strongly disagree and 5 = strongly agree)	T0, T2	Additional file 7: T0: 64 to 74 T2:145 to 155
	Questionnaire on perception towards the integration of PA in their team^a^	• Perception of the possibility of integrating PAs into the care team and eventual obstacles	5 items	Varied	T0, T2	Additional file 7: T0: 56 to 62 et 75 T2: 137 to 143 et 156
	Readiness To Partner With Patient and Family Advisers - Patient and Family Engagement in the Surgical Environment Module.Content last reviewed May 2017. Agency for Healthcare Research and Quality, Rockville, MD. https://www.ahrq.gov/hai/tools/ambulatorysurgery/sections/implementation/training-tools/readiness.html^a^			Varied	T0	Additional file 7: T0: 80 to 97

^a^ Questionnaire developed for this study by the research team

#### Questionnaires for patients

Measurements will be taken before the meeting with the PA (T0), immediately following this meeting (T1), and again 1 month after this meeting (T2). Two of the questionnaires used will be co-constructed with patients specifically for this study and assess: patients’ care experience (Additional file 4, questions 41 to 67) and patients’ experience with PAs (Additional file 4: questions 69 to 100 and 102 to 127), following the procedures and sequence of content validation outlined by Haynes and colleagues (1995) [53]. Three validated questionnaires will be used to assess patients’ psychological distress (K6) [49] (Additional file 4: questions 141 to 146), treatment adherence [47] (Additional file 4: questions 148 to 159), and ability to cope with cancer (CASE) [48] (Additional file 4: questions 129 to 140). Data variables of patients’ sociodemographic and clinical characteristics will be collected including sex, age, and type of cancer (Additional file 4, questions 22 to 39). To obtain a complete data set for 30 to 50 patients per site and based on a previously established response rate of 70% [54, 55], we will plan to recruit at least 40 to 70 patients from each site.

#### Questionnaires for patient advisors

For the PAs, outcome measurements will include one validated questionnaire to evaluate psychological distress (K6) [49] (Additional file 5: questions 84 to 89), and 3 questionnaires will be developed for this study: PA’s care experience (Additional file 5: questions 39 to 60), previous personal experience with PAs (Additional file 5: questions 62 to 82), and a descriptive review of their PA experience with patients during the project (Additional file 5: questions 91 to 241**).** Sociodemographic data will be collected also **(**Additional file 5: questions 19 to 36)**.**

#### Logbook

The PAs will be asked to complete a logbook entry after each meeting with a patient to document the duration of the meeting, topics discussed, difficulties experienced, perceived added value for the patient, and anything else they deem relevant. This logbook will be developed for this study (Additional file 6: questions 3 to 42).

#### Health and social care provider questionnaires

To better identify the organizational factors that can influence the integration of PAs in clinical oncology teams, we will administer validated questionnaires to health and social care provider participants at the beginning and end of the study to assess the evolution of their collaborative practices [50] (Additional file 7: questions 22 to 47), their capacity to make changes to their collaborative practice [51, 56, 57] (Additional file 7: questions 48 to 54), their perception about their relations with the patients and the PA’s [50] (Additional file 7: questions 64 to 74) and with a questionnaire that will be developed for this study about their openness to integrating PAs in their team and potential barriers to this integration (Additional file 7: questions 56 to 62 and 75). Sociodemographic data will be collected also **(**Additional file 7: questions 80 to 97)**.** Considering a minimum response rate of 35%, to obtain a sample size of 15 health and social care providers from each site, 43 will be invited, from each setting (*n* = 258 (6X43)), to respond to the questionnaires.

### Quantitative analyses

Statistical analyses will be performed using IBM SPSS Statistics version 25 [58] and SAS 9.4 [59]. Results with *p* < 0.05 will be considered significant. Descriptive statistics will be used to summarize each setting’s patients’ and PAs’ socio-demographic and clinical variables and the health and social care providers’ professional experience. Student t tests or non-parametric tests, such as the Mann-Whitney test, will be used for continuous variables. At the organizational level, the challenges encountered throughout the implementation of the PA program and the engagement and satisfaction of the health and social care oncology teams will be examined. At the clinical level, the effects on the patients, the PAs and the health and social care oncology teams will be assessed.

### Integrated knowledge translation

Our action research intervention will be conducted in close collaboration with the actors on the ground and is inspired by realistic evaluation methodology [30, 32]. The study will be conducted simultaneously with the co-construction of the intervention to enable knowledge and expertise sharing between the producers and the users of the results [27]. This strategy accelerates the adoption of new practices based on research results, for complex and innovative interventions [60, 61].

The study results will be shared with those participating in the implementation and assessment of the PA intervention according to the components of the Knowledge to Action framework [62]. This allows for recursive actions that will make it possible to write summaries together with each administrator/clinician representative and PAs on a site-by-site basis, offer exchange areas between researchers, health and social care teams and PAs, and implement results to improve health and social care service offerings and the health and social care system as a whole [63]. To this end, throughout the study we will regularly share the preliminary results with corresponding site. Also, over the 4 years of this study, we will hold annual events assembling researchers, decisionmakers, patients, PAs, health and social care oncology teams, and administrators to discuss the results and co-construct best practices based on them. In addition, our multidisciplinary research team includes stakeholders with expertise as: patients, family doctors, medical specialists, human factors, change management, administration, ethics, law, integrated knowledge translation, mixed methods.

### Deliverables

A toolbox (English and French) including the tools developed by the site stakeholders and the research team members to address the needs that emerge over the course of the co-construction and implementation phases of the study will be made available for all health and social care organizations. It will include: 1) educational material for the PA training dealing with subjects covered by the PA during their meetings with patients and the ethical and legal issues that are identified in the study; 2) implementation materials such as an assessment of the facilitating factors, PA recruitment process, confidentiality agreement forms for the PAs, PAs casefile notes templates that, once completed, are integrated into the accompanied patients’ health records, contracts regarding rights and responsibilities of the PAs and the study sites, a legal framework for the implementation of PA programs; 3) PA program promotion materials; 4) assessment tools, including questionnaires and their associated documentation, and informed consent forms. This toolbox will be freely available on the website of the research Chair for research and evaluation of leading edge technologies and practices – citizen and patient engagement in health system and organization transformation (https://chaireengagementpatient.openum.ca/), the website of the Centre for excellence in partnerships with patients and the public (https://ceppp.ca/en/), and the websites of the participating settings.

## Discussion

Research on peer support in oncology has shown promising results [6–13], particularly for helping people navigate the healthcare system. However, studies where patients who have experienced cancer care are integrated in the clinical teams are rare. This study will be the first to integrate PAs as full-fledged members of the clinical oncology team and to assess the potential effects at the clinical and organizational levels [64]. The only study we are aware of is currently underway in two regions in France and is exploring the effect of integrating “peers” who have experienced disease in clinical teams to support patients on communication and engagement in their care. Initial results indicate that recruiting peers and keeping them engaged in this role is difficult. However, unlike the study we will conduct, the peers are not full-fledged members of the clinical teams. Also, given that PAs will be integral members of the health and social care oncology team, our study will likely suggest means to successfully integrate care partnerships and services into clinical oncology teams. The originality of the PA’s role in our study will complement the body of research on peer support and patient navigators. Finally, given the mixed methods nature of our study, it will be possible to study variability between settings, and thus, delineate various situations that can be encountered and reinforce the external validity of our study.

Understanding the integration of patients into health and social care oncology care teams requires an analysis of the ethical and legal issues at play. For the most part, in this study, the potential issues are for PAs to access patient health records, the management of inform consent, the respect of confidentiality, the responsibility of the study settings towards the PAs and the patients, and the recognition and financial compensation of the PA’s as work [65]. These issues must be considered in conjunction with the role of the healthcare professionals and their relationships with the PA. Identifying the risks for the PAs, particularly psychological and social ones, as well as the implementation of terms and conditions to manage these risks are also part of the ethical challenges to document.

This study, funded in part by the Canadian Institutes of Health Research, will help to promote the care partnership approach at the clinical level and to develop the knowledge on this subject. It will also contribute to the knowledge regarding ethical and legal issues of developing care partnership for health and social care organizations interested in integrating PAs on clinical care teams.

### Study challenges and mitigation strategies

To carry out this research project, a certain number of challenges of the conception and implementation of PA and on the realization of the research are anticipated and actions to limit them have been planned.

Regarding the conception and implementation of PA, co-construction of the implementation and management mechanisms, including recruitment, training, and the definition of ethical and legal benchmarks, will be simultaneous. Differences in the perceptions and availability of the healthcare professionals and the PAs will be anticipated by planning for mitigating mechanisms throughout the study, particularly during the “site preparation” phase by creating training and discussing spaces to adequately define roles and responsibilities [66]. To consider the conditions of the COVID-19 pandemic, we will propose alternatives to the physical presence of PAs by teleconsultation.

For the realization of the research, it is important to maintain cohesion in the interdisciplinary research team that includes researchers from various universities and research centers as well as decision makers. Implementing steering and advisory committees as well as holding for an annual day to share and discuss the study’s progress and results will help maintain stakeholders’ interest. Knowing that care teams are overwhelmed by their workload, data collection will be done in direct collaboration with the settings and the questionnaires we will administer are relatively short. We will also benefit from the contextual knowledge and support of the clinical researcher members of our team. Finally, this study will collect an important quantity of data from various sites and sources which can lead to storage and data management challenges. Thanks to the qualitative data software QDA Miner and the quantitative data online platform and REDCap, we will be able to aptly manage, analyze, and integrate all data collected. In addition, to consider the COVID pandemic, we will follow the sites rules regarding research activities.

## Conclusion

Given oncology patients’ needs to be able to count on educational, emotional and partnership support over the course of their care trajectory, it is necessary to develop research projects that involve the various stakeholder groups and that yield results that decision makers can act on quickly. This study will evaluate the feasibility of integrating PAs into clinical oncology teams and highlight this integration procedure to identify the factors which enable or hinder their integration, to identify ethical and legal issues and means to address them, and to explore the effects of PA on patients, the PAs themselves, the care team, the administrators, and the organization of care. Should the results be promising, we will use them to plan and conduct a pragmatic randomized trial of the integration of PAs into clinical oncology care teams, to measure the impact of PAs integration on the teams on patients, and to better understand how this integration can improve the quality, safety and performance of our healthcare systems.

## Supplementary Information

**Additional file 1.** Individual Interview Guide (Management representatives - organizational level).

**Additional file 2.** Focus Group Guide (Members of the working committee)

**Additional file 3.** COREQ checklist (32 items) – Question number, Item, Guide questions/description and reference in this manuscript for each item on the checklist.

**Additional file 4.** PAROLE-Onco-2-Patients accompagnés-English-Code du dictionnaire de données - Variable name, question formulation and response options for each question in the patients questionnaires.

**Additional file 5.** PAROLE-Onco-2-Patient accompagnateur-English- Code du dictionnaire de données - Variable name, question formulation and response options for each question in the patients advisors questionnaires.

**Additional file 6.** Journal de bord-English-Code du dictionnaire de données - Variable name, question formulation and response options for each question in the patients advisors logbook.

**Additional file 7.** PAROLE-Onco-Professionnels-English-Code du dictionnaire de données - Variable name, question formulation and response options for each question in the professionals questionnaires.

## Data Availability

All the data collection tools, including individual interview guides, focus group guides and questionnaires, are available in the appendices. However, since this is an article on a study protocol, we are not presenting results, so we do not have a database to share in this article.

## Abbreviations

CASE-cancer: Communication and Attitudinal Self-Efficacy scale for cancer
CEVARMU: Centre d’Expertise pour les Victimes d’Amputation traumatique nécessitant une Réimplantation ou une revascularisation Microchirurgicale d’Urgence
CHU: Centre Hospitalier Universitaire
CHUM: Centre Hospitalier Universitaire de Montréal
CISSS: Centre Intégré de Santé et de Services Sociaux
CIUSSS: Centre Intégré Universitaire de Santé et de Services Sociaux
COVID-19: COronaVirus Disease 2019
CR-CHUM: Centre de recherche du Centre Hospitalier de l’Université de Montréal
CUSM: Centre Universitaire de Santé McGill
HSP: Health and Social care Professional
INESSS: Institut national d’excellence en santé et services sociaux
IsoN: Ingram School of Nursing
K6: Kessler Psychological Distress Scale – 6 items
PA: patient advisor
PAROLE-onco: Patient AdvisoR, an Organizational resource as a Lever for an Enhanced Oncology patient experience
PQC: Programme Québécois de Cancérologie
RCN: Rossy Cancer Network
REDCap: Research Electronic Data Capture
