# Correction to: The patient advisor, an organizational resource as a lever for an enhanced oncology patient experience (PAROLEonco): a longitudinal multiple case study protocol

**DOI:** 10.1186/s12913-021-06065-4

**Published:** 2021-01-14

**Authors:** M. P. Pomey, M. de Guise, M. Desforges, K. Bouchard, C. Vialaron, L. Normandin, M. Iliescu-Nelea, I. Fortin, I. Ganache, C. Régis, Z. Rosberger, D. Charpentier, L. Bélanger, M. Dorval, D. P. Ghadiri, M. Lavoie-Tremblay, A. Boivin, J. F. Pelletier, N. Fernandez, A. M. Danino

**Affiliations:** 1 Centre de recherche du Centre Hospitalier de l’Université de Montréal (CR-CHUM), 850, rue Saint-Denis, Montréal, Québec H2X 0A9 Canada; 2 Centre d’Excellence pour le Partenariat avec les Patients et le Public, 900, rue Saint-Denis, Porte S03.900, Montréal, Québec H2X 0A9 Canada; 3 École de santé publique de l’université de Montréal-Département de gestion, évaluation et politique de santé, 7101 Av du Parc, Montréal, Québec H3N 1X9 Canada; 4 Université de Montréal – Faculté de Médecine, 2900 boulevard Edouard-Montpetit, Montréal, Québec H3T 1J4 Canada; 5 Institut national d’excellence en santé et services sociaux (INESSS), 2021, avenue Union, 12e étage, bureau 1200, Montréal, Québec H3A 2S9 Canada; 6 Centre Intégré Universitaire de santé et services sociaux de l’Est-de-l’Île-de Montréal, Hôpital de Maisonneuve-Rosemont, 5415, boulevard de l’Assomption, Montréal, Québec H1T 2M4 Canada; 7 CHU de Québec-Université Laval, 10, Rue de l’Espinay, Québec, Québec G1L 3L5 Canada; 8 Université de Montréal – Faculté de Droit, 3101 chemin de la Tour, Montréal, Québec H3T 1J7 Canada; 9 Lady Davis Institute for Medical Research, Jewish General Hospital & McGill University, Gerald Bronfman Department of Oncology, 5100 de Maisonneuve Blvd West, Montréal, Québec H4A 3T2 Canada; 10 Centre Hospitalier Universitaire de Montréal (CHUM), 1000 rue Saint-Denis, Montréal, Québec H2X 0C1 Canada; 11 Université Laval – Faculté de pharmacie, 050, avenue de la Médecine, Québec, Québec G1V 0A6 Canada; 12 Centre de recherche du CHU de Québec-Université Laval, 1050 chemin Sainte-Foy, Québec, Québec G1S4L8 Canada; 13 Centre de recherche du CISSS Chaudière Pomey et al. BMC Health Services Research (2021) 21:10 Page 10 of 12 Appalaches, 143 rue Wolfe, Lévis, Québec, G6V 3Z1 Canada; 14 HEC Montréal, Department of management, 3000, chemin de la Côte-Sainte-Catherine, Montréal, Québec H3T 2A7 Canada; 15 McGill University, Ingram School of Nursing (IsoN), 680 Sherbrooke Street West, Montréal, Québec H3A 2M7 Canada; 16 Centre Universitaire de Santé McGill (CUSM), 1650, avenue Cedar, Montréal, Québec H3G 1A4 Canada; 17 Centre de Recherche de l’Institut universitaire en santé mentale de Montréal, 7331 Rue Hochelaga, Montréal, Québec H1N 3V2 Canada

**Correction to: BMC Health Serv Res 21, 10 (2021)**

**https://doi.org/10.1186/s12913-020-06009-4**

Following the publication of the original article [1], it was noted that due to a typesetting error Fig. 1 needs to be updated with a new version.

**Fig. 1:**
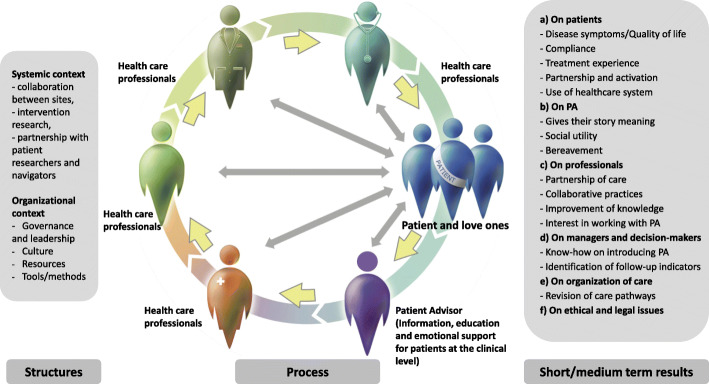
Conceptual framework and the 4 objectives

The updated figure has been included in this correction, and the original article has been corrected.
