# Supplementary material for: The patient advisor, an organizational resource as a lever for an enhanced oncology patient experience (PAROLE-onco): a longitudinal multiple case study protocol
Source: BMC Health Serv Res. 2021 Jan 4;21:10. doi: 10.1186/s12913-020-06009-4 (PMC7780212; doi:10.1186/s12913-020-06009-4)
Supplement: Supplementary file 1 — Additional file 1. Individual Interview Guide (Management representatives - organizational level). [file 12913_2020_6009_MOESM1_ESM.docx]

Individual Interview Guide

(Management representatives - organizational level)

PREAMBLE

- Thank the participant for agreeing to answer our questions as part of our study.
- Specify that the interview will last approximately 30 minutes (CEOs, Board Chairs, and other key stakeholder categories).

**Interview Rules**

- Everything that will be said will remain **confidential**. To protect your privacy, your name will not appear anywhere, and you will remain completely anonymous.
- At any time during our conversation, let me know if you have any **questions** or if you prefer **not to answer** a particular question. Also, you may decide to **stop the interview** at any time.
- Remember that we want to know **what you think and feel**. So, there is no right or wrong answer.
- Ensure that the participant agrees to have the interview taped.

**Information and Consent Form**

- Ensure that the participant has signed the ICF.

**[Start recording]**

**BACKGROUND**

This research project aims to evaluate the implementation of oncology patient advisors (PAs) in health care teams. You will have the opportunity to express yourself and share your experiences and opinions on the subject during this meeting. The themes that will be discussed are the following: your expectations, potential facilitating and hindering factors, challenges and sustainability.

1. What are your expectations regarding the involvement of PAs in relation to your function?

1. What specific resources (human, financial, infrastructural or informational) could and/or should be allocated by the institution for the integration of PAs?
2. What elements of organizational culture might facilitate or hinder the integration of Pas with other patients?

- *Institutional leadership?*
- *Policies and procedures?*
- *Teamwork?*

1. In your opinion, what are/will be the effects of integrating PAs?

*For the moderator:*

*Which dimensions are concerned according to you?*

- - *on patients (Disease Symptoms/Quality of Life, Adherence, Care Experience, Partnership and Activation, Health System Utilization)*
  - *on PAs (Gives meaning to their history, Social utility, Grief)*
  - *on the team (Partnership of Care, Collaborative Practices, Knowledge Enhancement and Transfer, Interest in working with IPs)*
  - *on organization (redesigning care pathways)*

1. What impact do you think the integration of PAs will have on workloads?
2. In your opinion, what are the legal and/or ethical issues related to the participation of PAs in your program? How do you plan to address them?
3. What do you think about the sustainability of integrating PAs into your institution / program?
4. Do you think your institution is ready to integrate PAs?
5. Do you have anything else you would like to add? Other topics that you would like to talk about and that you think are important to explore?
6. In conclusion, what is **the most important message** you would like to see come out of the interview?

*Thank you very much for your participation in this interview.*
