# Supplementary material for: The patient advisor, an organizational resource as a lever for an enhanced oncology patient experience (PAROLE-onco): a longitudinal multiple case study protocol
Source: BMC Health Serv Res. 2021 Jan 4;21:10. doi: 10.1186/s12913-020-06009-4 (PMC7780212; doi:10.1186/s12913-020-06009-4)
Supplement: Supplementary file 2 — Additional file 2. Focus Group Guide (Members of the working committee) [file 12913_2020_6009_MOESM2_ESM.docx]

Focus Group Guide

(Members of the working committee)

PREAMBLE

**Information and Consent Form**

Ensure that the participants have signed the ICF.

Ensure that participants agree to have the focus group recorded.

**[Start video recording]**

This research project aims to evaluate the implementation of oncology patient advisors (Pas) in health care teams. You will have the opportunity to express yourself and share your experiences and opinions on the subject during this meeting. We will base this focus group on a few questions, which I will present to you later. The themes that will be discussed are the following: definition of PA, factors facilitating and hindering intervention, effects, issues, and sustainability.

**Group rules**

- Thank the participants for agreeing to answer our questions as part of our study.
- Please note that the group will last approximately 120 minutes.
- Announcement of audio and video recordings
- Explanation of and reasons for the presence of remote observers
- Closing cell phones
- Anonymity and confidentiality are respected, and participants are identified by their first name only.
- No right or wrong answers
- Invitation to react to what others say and not to interrupt
- This is not an individual performance test.

1. I would like to start with a round table introduction. In turn, I invite you to state your first name and tell us what led you to sit on this working committee.
2. How do you define the integration of PAs into your care teams?

For the moderator: get the group to clearly define what the concept of integration refers to....

1. What do you think the role of the PA is?

What distinguishes him/her from other members of the health care team?

1. Why do you think your organization/program decided to include PA on health care teams? Is your facility ready? Explain…
2. To your knowledge, what **factors** could promote the integration of **PAs**?
3. To your knowledge, what factors could hinder the integration of PAs?

In an ideal world, what should be the specific resources (human, financial, infrastructural, or informational) allocated by the institution for the integration of PAs?

1. In your opinion, what are/will be the effects of the integration of PAs ?

For the moderator:

Which dimensions are concerned according to you?

- - - On patients (disease symptoms/quality of Life, adherence, care experience, partnership and activation, health system utilization)
    - On PAs (gives meaning to their history, social utility, grief)
    - On the team (partnership of care, collaborative practices, knowledge enhancement and transfer, interest in working with PAs)
    - On organization (redesigning care pathways)

1. What impact do you think the integration of PAs will have on workloads?
2. Do you anticipate ethical and/or legal issues? If so, which ones? How do you plan to address them?
3. What do you think about the sustainability of integrating PAs into your institution/program?
4. Do you have anything else you would like **to add**? Any other topics that you would like to discuss and **feel it is important to explore**?

Thank you very much for your participation in this focus group.
