# Supplementary material for: The patient advisor, an organizational resource as a lever for an enhanced oncology patient experience (PAROLE-onco): a longitudinal multiple case study protocol
Source: BMC Health Serv Res. 2021 Jan 4;21:10. doi: 10.1186/s12913-020-06009-4 (PMC7780212; doi:10.1186/s12913-020-06009-4)
Supplement: Supplementary file 3 — Additional file 3. COREQ checklist (32 items) – Question number, Item, Guide questions/description and reference in this manuscript for each item on the checklist. [file 12913_2020_6009_MOESM3_ESM.docx]

| No | Item | Guide questions/description | Reference in this manuscript |
| --- | --- | --- | --- |
| Domain 1: Research team and reflexivity | | | |
| Personal Characteristics | | | |
| 1. | Interviewer/facilitator | Which author/s conducted the interview or focus group? | Section “Methods/design”, page 10, line 20 and 44  Karine Bouchard (KB)  Louise Normandin (LN)  Monica Iliescu-Nelea (MIN) |
| 2. | Credentials | What were the researcher's credentials? E.g. PhD, MD | KB (M.Sc.)  LN (PhD)  MIN (Ing. PhD) |
| 3. | Occupation | What was their occupation at the time of the study? | KB: research assistant and coordinator  LN: research assistant and coordinator  MIN: research assistant and coordinator |
| 4. | Gender | Was the researcher male or female? | KB: female  LN: female  MIN: female |
| 5. | Experience and training | What experience or training did the researcher have? | KB has conducted and published qualitative research.  LN has conducted and published qualitative research.  MIN has conducted and published qualitative research. |
| Relationship with participants | | | |
| 6. | Relationship established | Was a relationship established prior to study commencement? | N/A since it is an article on the protocol, so no mention will be made of the participants recruited. |
| 7. | Participant knowledge of the interviewer | What did the participants know about the researcher? e.g. personal goals, reasons for doing the research | Section “Methods/design”, page 10, line 20-22 and line 44-48. |
| 8. | Interviewer characteristics | What characteristics were reported about the interviewer/facilitator? e.g. Bias, assumptions, reasons and interests in the research topic | KB, LN and MIN have experience in qualitative research, and they all have an interest in patient partnerships and have already been involved in projects related to the integration of patient advisors or resources into the care trajectory. |
| Domain 2: study design | | | |
| Theoretical framework | | | |
| 9. | Methodological orientation and Theory | What methodological orientation was stated to underpin the study? e.g. grounded theory, discourse analysis, ethnography, phenomenology, content analysis | Section “Methods/design”, page 11, line 33-46 |
| Participant selection | | | |
| 10. | Sampling | How were participants selected? e.g. purposive, convenience, consecutive, snowball | Section “Methods/design”, page 9, line 11-13 |
| 11. | Method of approach | How were participants approached? e.g. face-to-face, telephone, mail, email | Section “Methods/design”, page 9, line 15-17 |
| 12. | Sample size | How many participants were in the study? | N/A since it is an article on the protocol, so no mention will be made of the participants recruited. |
| 13. | Non-participation | How many people refused to participate or dropped out? Reasons? | N/A since it is an article on the protocol, so no mention will be made of the participants recruited. |
| Setting | | | |
| 14. | Setting of data collection | Where was the data collected? e.g. home, clinic, workplace | Section “Methods/design”, page 10, line 17-19 and 42-44 |
| 15. | Presence of non-participants | Was anyone else present besides the participants and researchers? | No |
| 16. | Description of sample | What are the important characteristics of the sample? e.g. demographic data, date | Section “Methods/design”, page 8 line 50-52 and page 9, line 7-11 |
| Data collection | | | |
| 17. | Interview guide | Were questions, prompts, guides provided by the authors? Was it pilot tested? | Section “Methods/design”, page 10, line 13-17 and 38-42 and appendices 1 and 2. |
| 18. | Repeat interviews | Were repeat interviews carried out? If yes, how many? | N/A since it is an article on the protocol, so no mention will be made of the interviews conducted. |
| 19. | Audio/visual recording | Did the research use audio or visual recording to collect the data? | Section “Methods/design”, page 10, line 24 and 48. |
| 20. | Field notes | Were field notes made during and/or after the interview or focus group? | Section “Methods/design”, page 10, line 24 and 50. |
| 21. | Duration | What was the duration of the interviews or focus group? | Section “Methods/design”, page 10, line 26 and 50, page 11, line 7. |
| 22. | Data saturation | Was data saturation discussed? | Section “Methods/design”, page 9, line 52, page 10, line 7-9 and page 11, line 7-9. |
| 23. | Transcripts returned | Were transcripts returned to participants for comment and/or correction? | It is not scheduled. |
| Domain 3: analysis and findings | | | |
| Data analysis | | | |
| 24. | Number of data coders | How many data coders coded the data? | Section “Methods/design”, page 11, line 37-39. |
| 25. | Description of the coding tree | Did authors provide a description of the coding tree? | N/A since this manuscript relates to the protocol, it does not present the results. |
| 26. | Derivation of themes | Were themes identified in advance or derived from the data? | Section “Methods/design”, page 11, line 33-46. |
| 27. | Software | What software, if applicable, was used to manage the data? | Section “Methods/design”, page 11, line 46. |
| 28. | Participant checking | Did participants provide feedback on the findings? | N/A since this manuscript relates to the protocol, it does not present the results. |
| Reporting | | | |
| 29. | Quotations presented | Were participant quotations presented to illustrate the themes / findings? Was each quotation identified? e.g. participant number | N/A since this manuscript relates to the protocol, it does not present the results. |
| 30. | Data and findings consistent | Was there consistency between the data presented and the findings? | N/A since this manuscript relates to the protocol, it does not present the results. |
| 31. | Clarity of major themes | Were major themes clearly presented in the findings? | N/A since this manuscript relates to the protocol, it does not present the results. |
| 32. | Clarity of minor themes | Is there a description of diverse cases or discussion of minor themes? | N/A since this manuscript relates to the protocol, it does not present the results. |
