# Supplementary material for: The patient advisor, an organizational resource as a lever for an enhanced oncology patient experience (PAROLE-onco): a longitudinal multiple case study protocol
Source: BMC Health Serv Res. 2021 Jan 4;21:10. doi: 10.1186/s12913-020-06009-4 (PMC7780212; doi:10.1186/s12913-020-06009-4)
Supplement: Supplementary file 4 — Additional file 4. PAROLE-Onco-2-Patients accompagnés-English-Code du dictionnaire de données - Variable name, question formulation and response options for each question in the patients questionnaires. [file 12913_2020_6009_MOESM4_ESM.pdf]

PAROLE-Onco-2-Patients accompagnés-English

Guide des codes

Code du dictionnaire de données

09-10-2020 21:02

Collapse all instruments

	#	Nom de variable/champ	Étiquette de champ<br>*Note de champ*	Attributs de champ (type de champ, validation, choix, logique de branchement, calculs, etc.)
Formulaire : **Participant identification form** (participant_identification_form) Expand				
Formulaire : **Integrated consent** (integrated_consent) Expand				
Formulaire : **Socio-demographic questionnaire** (sociodemographic_questionnaire) Collapse				
	21	pa_bidon6pa	To begin the questionnaire, here are some socio-demographic questions.	descriptive
	22	pa_sexepa	You are... ?	radio<br>1 A woman<br>2 A man<br>Alignement personnalisé : LV
	23	pa_agepa	What is your age group?	radio<br>1 24 years old and under<br>2 25-34 years old<br>3 35-44 years old<br>4 45-54 years old<br>5 55-64 years old<br>6 65-74 years old<br>7 75 ans years old and over<br>Alignement personnalisé : LV
	24	pa_nepa	Where were you born?	radio<br>1 In Quebec<br>2 Elsewhere in Canada<br>3 Outside Canada<br>4 Does not answer<br>Alignement personnalisé : LV
	25	pa_neepascanadapa<br>Afficher le champ UNIQUEMENT si :<br>[pa_nepa] = '3'	If you were born outside of Canada, please specify the country.	text<br>Alignement personnalisé : LV
	26	pa_anqcpa<br>Afficher le champ UNIQUEMENT si :<br>[pa_nepa] = '2' or [pa_nepa] = '3'	How many years have you lived in Quebec?	radio<br>1 Less than 5 years<br>2 5 to 10 years<br>3 More than 10 years<br>4 Don't know<br>Alignement personnalisé : LV

	27	ppa57pa	In which administrative region do you live?	radio<br>0 Bas-Saint-Laurent<br>1 Saguenay-Lac-Saint-Jean<br>2 Capitale-Nationale (Québec)<br>3 Mauricie-et-Centre-du-Québec<br>4 Estrie<br>5 Montréal<br>6 Outaouais<br>7 Abitibi-Témiscamingue<br>8 Côte-Nord<br>9 Nord-du-Québec<br>10 Gaspésie-Îles-de-la-Madeleine<br>11 Chaudière-Appalaches<br>12 Laval<br>13 Lanaudière<br>14 Laurentides<br>15 Montérégie<br>16 Nunavik<br>17 Terres-Cries-de-la-Baie-James<br><br>Alignement personnalisé : LV
	28	pa_nbmenagepa	How many people compose your household and reside permanently in your home, including you?	text (number)<br>Alignement personnalisé : LV
	29	pa_nbchargepa	How many of the people in your household are dependent on you?	text (number)<br>Alignement personnalisé : LV
	30	pa_situationpa	What type of situation best suits the composition of your household?	radio<br>1 Person living alone in the household<br>2 Couple without children at home<br>3 Couple with children at home<br>4 Single-parent family (children of any age)<br>5 Family with other people<br>6 Related persons only (siblings, etc.)<br>7 Non-related persons only (roommates)<br>8 Other (multiple families, etc.)<br><br>Alignement personnalisé : LV
	31	pa_situationautrepa<br>Afficher le champ UNIQUEMENT si :<br>[pa_situationpa] = '8'	If you answered "Other", please specify the composition of your household.	text<br>Alignement personnalisé : LV
	32	ppa_entou_v2	Is there one or more people in your entourage (spouse, child, friend, other) who can help you if necessary with your current medical follow-up?	radio<br>1 Yes<br>2 No<br><br>Alignement personnalisé : LV
	33	ppa_entouprecis_v2<br>Afficher le champ UNIQUEMENT si :<br>[ppa_entou_v2] = '1'	This person is your (you can choose more than one answer) :	checkbox<br>1 ppa_entouprecis_v2___1 Spouse<br>2 ppa_entouprecis_v2___2 Child<br>3 ppa_entouprecis_v2___3 Parent<br>4 ppa_entouprecis_v2___4 Friend<br>5 ppa_entouprecis_v2___5 Other<br><br>Alignement personnalisé : LV
	34	pro_entouautre_v2<br>Afficher le champ UNIQUEMENT si :<br>[ppa_entouprecis_v2(5)] = '1'	If you answered "Other", please specify:	text<br>Alignement personnalisé : LV

	35	pa_educpa	What is the highest level of education you have completed?	radio<br>1 No diploma<br>2 Elementary education<br>3 Partial studies at high school (I-IV)<br>4 High School Diploma (Sec V or Grade 12)<br>5 Partial studies in a CEGEP, technical school or professional training school<br>6 Diploma or certificate from a CEGEP, technical school or professional training school<br>7 Partial studies at university<br>8 University degree<br>9 Other<br><br>Alignement personnalisé : LV
	36	pa_eduautrepa<br><br>Afficher le champ UNIQUEM ENT si :<br>[pa_educpa] = '9'	If you checked "Other", please specify the highest level of education you have completed.	text<br>Alignement personnalisé : LV
	37	pa_occupa	In the past 12 months, what was your main occupation?	radio<br>1 Full-time worker<br>2 Part-time worker<br>3 Student<br>4 Volunteer/Unpaid Worker<br>5 Retired<br>6 Homemaker<br>7 On maternity/paternity leave<br>8 Employment Insurance claimant<br>9 Income assistance recipient (income security)<br>10 Other<br><br>Alignement personnalisé : LV
	38	pa_occuautrepa<br><br>Afficher le champ UNIQUEM ENT si :<br>[pa_occupa] = '10'	If you answered "Other", please specify what was your main occupation in the last 12 months.	text<br>Alignement personnalisé : LV
	39	pa_finanpa	How do you feel about your financial situation compared to people your own age?	radio<br>1 You consider yourself financially comfortable<br>2 You consider that your income is sufficient to meet your basic needs or those of your family.<br>3 You consider yourself poor<br>4 You consider yourself very poor<br><br>Alignement personnalisé : LV
	40	sociodemographic_questionnaire_complete	En-tête de section : *Form Status*<br>Complete?	dropdown<br>0 Incomplete<br>1 Unverified<br>2 Complete

Formulaire : **Patient's care experience questionnaire** (patients_care_experience_questionnaire) Collapse

	41	pa_recrupa	En-tête de section : *The following questions focus on your clinical experience and the information provided by your health care team. By health care team, we mean the various health care professionals who work together: doctors, nurses, pivot nurses, pharmacists, nutritionists, psychologists, social workers, physiotherapists, etc...*<br>Where were you given this questionnaire?	radio<br>1 At CHUM<br>2 At CIUSSS Mauricie-Centre-du-Québec<br>3 At CIUSSS de l'Est-de-l'Île-de-Montréal<br>4 At CHU de Québec-Université Laval<br>5 At CISSS Laval<br>6 At CIUSSS du Centre-Ouest-de-l' Île-de-Montréal<br>7 At CISSS de la Gaspésie<br>8 At CIUSSS du Saguenay-Lac-Saint-Jean<br>9 At CUSM<br>Alignement personnalisé : LV
	42	pa_suivipa	Your current medical follow-up takes place in the context of :	radio<br>1 An episode of cancer<br>2 An episode of cancer that led to an oncogenetic consultation<br>3 A consultation in oncogenetic<br>Alignement personnalisé : LV
	43	pa_cancerpa<br>Afficher le champ UNIQUEMENT si :<br>[pa_suivipa] = '1' or [pa_suivipa] = '2'	What is the main type of cancer you are currently being treated for?	radio<br>1 Oral cavity<br>2 Brain<br>3 Colorectal (colon, rectum)<br>4 Cervix<br>5 Endometrium / Uterus<br>6 Stomach / Esophagus<br>7 Liver<br>8 Leukemia<br>9 Non-Hodgkin's lymphoma<br>10 Melanoma<br>11 Ovary<br>12 Pancreas<br>13 Skin (without melanoma)<br>14 Lung / Bronchus<br>15 Prostate<br>16 Kidney<br>17 Breast<br>18 Thyroid<br>19 Bladder<br>20 Other cancer<br>Alignement personnalisé : LV
	44	pa_cancerautrepa<br>Afficher le champ UNIQUEMENT si :<br>[pa_cancerpa] = '20'	If you are currently being followed for a type of cancer other than those listed, please specify which one:	text<br>Alignement personnalisé : LV
	45	pa_cancernewpa<br>Afficher le champ UNIQUEMENT si :<br>[pa_suivipa] = '1' or [pa_suivipa] = '2'	Is this cancer :	radio<br>1 A new cancer (a cancer treated for the first time)<br>2 A recurrence of a cancer treated in the past<br>3 A second or third cancer<br>4 Don't know<br>Alignement personnalisé : LV

	46	pa_cancermetaspa<br>Afficher le champ UNIQUEMENT si :<br>[pa_suivipa] = '1' or [pa_suivipa] = '2'	Is it a metastatic cancer?	radio<br>1 Yes<br>2 No<br>Alignement personnalisé : LV
	47	pa_canceranpa<br>Afficher le champ UNIQUEMENT si :<br>[pa_suivipa] = '1' or [pa_suivipa] = '2'	When were you diagnosed with the cancer you are being treated for now?	radio<br>1 Less than one month<br>2 Between 1 and 6 months<br>3 Between 7 and 11 months<br>4 Between 1 and 3 years<br>5 Between 3 and 5 years<br>6 More than 5 years<br>7 Don't know<br>Alignement personnalisé : LV
	48	pa_cancer5anpa<br>Afficher le champ UNIQUEMENT si :<br>[pa_canceranpa] = '6'	If you have been diagnosed with cancer for more than 5 years, please specify the number of years.	text (number)<br>Alignement personnalisé : LV
	49	pa_trajectpa<br>Afficher le champ UNIQUEMENT si :<br>[pa_suivipa] = '1' or [pa_suivipa] = '2'	At the time of answering this questionnaire, what step best describes where you are in your care trajectory to treat this cancer?	radio, Required<br>1 Before starting treatment<br>2 Treatment in progress<br>3 Treatment completed<br>Alignement personnalisé : LV
	50	pa_traitpa<br>Afficher le champ UNIQUEMENT si :<br>[pa_trajectpa] = '2' or [pa_trajectpa] = '3'	What type of treatment or surgery did you receive (check all that apply)?	checkbox<br>1 pa_traitpa___1 Vein Chemotherapy (Intravenous)<br>2 pa_traitpa___2 Chemotherapy with swallowable tablets<br>3 pa_traitpa___3 Hormonal therapy (antihormones)<br>4 pa_traitpa___4 Radiotherapy or brachytherapy<br>5 pa_traitpa___5 Partial Surgery (remove the tumor while preserving as much healthy tissue as possible)<br>6 pa_traitpa___6 Complete Surgery (completely remove the affected organ)<br>7 pa_traitpa___7 Reconstructive surgery<br>8 pa_traitpa___8 Transplantation or grafting<br>9 pa_traitpa___9 Medication for symptoms (pain, nausea)<br>10 pa_traitpa___10 Alternative Medicine<br>11 pa_traitpa___11 Other<br>12 pa_traitpa___12 Don't know<br>Alignement personnalisé : LV
	51	pa_traitautrepa<br>Afficher le champ UNIQUEMENT si :<br>[pa_traitpa(11)] = '1'	If you have received any type of treatment other than those listed, please specify:	text<br>Alignement personnalisé : LV

	52	pa_traitdebutpa<br><br>Afficher le champ UNIQUEM ENT si :<br>[pa_trajectpa] = '2' or [pa_traj ectpa] = '3'	This (or these) treatment(s) was (or were) started:	radio<br>1 less than a month ago<br>2 between 1 and 6 months ago<br>3 between 7 and 11 months ago<br>4 between 1 and 3 years<br>5 between 3 and 5 years<br>6 more than 5 years ago<br>7 Don't know<br><br>Alignement personnalisé : LV
	53	pa_traitdebut5pa<br><br>Afficher le champ UNIQUEM ENT si :<br>[pa_traitdebutpa] = '6'	If your treatments began more than 5 years ago, please indicate the number of years.	text<br>Alignement personnalisé : LV
	54	pa_genetiquepa<br><br>Afficher le champ UNIQUEM ENT si :<br>[pa_suivipa] = '1'	Have you consulted in oncogenetic or undergone genetic testing to find out your hereditary predisposition to cancer?	radio<br>1 Yes<br>2 No<br>3 Don't know<br><br>Alignement personnalisé : LV
	55	pa_bidon7<br><br>Afficher le champ UNIQUEM ENT si :<br>[pa_suivipa] = '3' or [pa_suivip a] = '2' or [pa_genetiquepa] ="1"	YOUR MEDICAL FOLLOW-UP TOOK PLACE IN THE CONTEXT OF AN ONCOGENETIC CONSULTATION	descriptive<br>1 Décision thérapeutique (décision chirurgicale ou thérapie ciblée) 2, Pour connaitre mon statut génétique à la suite d'un diagnostic de cancer 3, Pour connaitre mon statut génétique suite à l'identification d'une mutation génétique dans la famille 4, Pour connaitre mon statut génétique en raison de mon historique familial de cancer
	56	pa_motifgenpa<br><br>Afficher le champ UNIQUEM ENT si :<br>[pa_suivipa] = '3' or [pa_suivip a] = '2' or [pa_genetiquepa] ="1"	Why are you undergoing oncogenetic consultation OR genetic testing?	radio<br>1 Therapeutic decision (surgical decision or targeted therapy)<br>2 To find out my genetic status following a cancer diagnosis<br>3 To know my genetic status following the identification of a genetic mutation in the family<br>4 To know my genetic status due to my family history of cancer<br><br>Alignement personnalisé : LV
	57	pa_oncogenpa<br><br>Afficher le champ UNIQUEM ENT si :<br>[pa_suivipa] = '3' or [pa_suivip a] = '2' or [pa_genetiquepa] ="1"	At the time of answering this questionnaire, what step best describes where you are in your trajectory in oncogenetic?	radio<br>1 You have just attended an information meeting in oncogenetic<br>2 You are waiting for the result of your genetic test<br>3 You have just received your genetic test result.<br>4 You are a carrier of a genetic mutation and you are considering preventive surgery.<br><br>Alignement personnalisé : LV
	58	pa_resultdatepa<br><br>Afficher le champ UNIQUEM ENT si :<br>[pa_oncogenpa] = '3' or [pa_o ncogenpa] = '4'	You received your genetic test result :	radio<br>1 Less than 1 month ago<br>2 Between 1 and 6 months ago<br>3 Between 7 and 11 months ago<br>4 Between 1 and 3 years ago<br>5 Between 3 and 5 years ago<br>6 More than 5 years ago<br>7 Don't know<br><br>Alignement personnalisé : LV

	59	pa_result5pa<br><br>Afficher le champ UNIQUEMENT si :<br>[pa_resultdatepa] = '6'	If you received your result more than 5 years ago, please indicate the number of years.	text<br>Alignement personnalisé : LV
	60	pa_mastectpa<br><br>Afficher le champ UNIQUEMENT si :<br>[pa_oncogenpa] = '3' or [pa_oncogenpa] = '4'	Have you had or are you considering having a preventative mastectomy (breast removal) to reduce your risk of breast cancer?	radio<br>0 | Yes, I had this surgery<br>1 | Yes, I am considering this surgery<br>2 | No, I have not had this surgery and I am not considering it.<br>3 | Not applicable<br><br>Alignement personnalisé : LV
	61	pa_mastectanpa<br><br>Afficher le champ UNIQUEMENT si :<br>[pa_mastectpa] = '0'	In what year did you have your preventive mastectomy?	text<br>Alignement personnalisé : LV
	62	pa_reconstpa<br><br>Afficher le champ UNIQUEMENT si :<br>[pa_mastectpa] = '0'	Have you had a breast reconstruction?	radio<br>1 | Yes<br>2 | No<br><br>Alignement personnalisé : LV
	63	pa_reconsttypepa<br><br>Afficher le champ UNIQUEMENT si :<br>[pa_reconstpa] = '1'	What type of breast reconstruction have you had?	radio<br>1 | Reconstruction by adding breast implants<br>2 | Reconstruction from your own tissues<br>3 | Don't know<br><br>Alignement personnalisé : LV
	64	pa_ovariectpa<br><br>Afficher le champ UNIQUEMENT si :<br>[pa_oncogenpa] = '3' or [pa_oncogenpa] = '4'	Have you had or are you considering preventive bilateral salpingo ovariectomy (removal of ovaries and tubes) to reduce your risk of ovarian cancer?	radio<br>0 | Yes, I had this surgery<br>1 | Yes, I am considering this surgery<br>2 | No, I have not had this surgery and I am not considering it.<br>3 | Not applicable<br><br>Alignement personnalisé : LV
	65	pa_ovariectanpa<br><br>Afficher le champ UNIQUEMENT si :<br>[pa_ovariectpa] = '0'	In what year did you have your preventive ovariectomy?	text<br>Alignement personnalisé : LV
	66	pa_infopa	During your meetings with the health care team, the information provided allowed you to fully understand:	checkbox<br>1 | pa_infopa___1 | Your condition (stakes, diagnosis, evolution)<br>2 | pa_infopa___2 | Your examinations (tests and investigations)<br>3 | pa_infopa___3 | Your options, benefits and side effects<br>4 | pa_infopa___4 | Your follow-up and care<br>5 | pa_infopa___5 | None of the above<br>6 | pa_infopa___6 | Other<br><br>Alignement personnalisé : LV
	67	pa_infoautrepa<br><br>Afficher le champ UNIQUEMENT si :<br>[pa_infopa(6)] = '1'	If you checked "Other", please specify.	text<br>Alignement personnalisé : LV
	68	patients_care_experience_questionnaire_complete	En-tête de section : *Form Status*<br><br>Complete?	dropdown<br>0 | Incomplete<br>1 | Unverified<br>2 | Complete

Formulaire : **Patient's experience with PAs questionnaire** (patients_experience_with_pas_questionnaire) Collapse

	69	pa_jumelage	Have you ever been accompanied by someone (this may be one or more meetings) who has had the same experience as you (cancer, surgery or genetic testing experience)? In health care settings, these people are called "patients advisors".	radio<br>0 Yes<br>1 Not yet but I wish to benefit from it<br>2 No and I don't think I need it<br><br>Alignement personnalisé : LV
	70	pa_bidon2_v2_v2<br><br>Afficher le champ UNIQUEMENT si :<br>[pa_jumelage] = '0'	What topics did you discuss with the patient advisor? (Check all that apply)	descriptive
	71	pa_themeorg<br><br>Afficher le champ UNIQUEMENT si :<br>[pa_jumelage] = '0'	Organizational aspects	checkbox<br>0 pa_themeorg___0 Her/His role as a patient advisor<br>1 pa_themeorg___1 The role of different health care professionals<br>2 pa_themeorg___2 The role of external and internal organizations<br>3 pa_themeorg___3 The care trajectory<br>4 pa_themeorg___4 Rights as a patient (e.g., refusing treatment, asking questions)<br>5 pa_themeorg___5 Where and how to get to medical appointments<br>6 pa_themeorg___6 Financial support for patients and transportation assistance<br>7 pa_themeorg___7 The Parole-Onco Research Project<br>8 pa_themeorg___8 Other information<br><br>Alignement personnalisé : LV
	72	pa_asp_org_autre<br><br>Afficher le champ UNIQUEMENT si :<br>[pa_themeorg(8)] = '1'	If other information, please specify which one:	text<br>Alignement personnalisé : LV

<table>
<tr><td></td><td>73</td><td>pa_themecli

Afficher le champ UNIQUEM ENT si :
[pa_jumelage] = '0'</td><td>Clinical aspects</td><td>checkbox
<table><tr><td>0</td><td>pa_themecli___0</td><td>The announcement of the cancer diagnosis or genetic predisposition by the doctor</td></tr><tr><td>1</td><td>pa_themecli___1</td><td>Cancer</td></tr><tr><td>2</td><td>pa_themecli___2</td><td>Genetic testing</td></tr><tr><td>3</td><td>pa_themecli___3</td><td>Therapeutic options in the case of cancer</td></tr><tr><td>4</td><td>pa_themecli___4</td><td>Ways to reduce risk in carriers of a gene mutation that increases the risk of cancer</td></tr><tr><td>5</td><td>pa_themecli___5</td><td>Surgical and reconstructive options for cancer</td></tr><tr><td>6</td><td>pa_themecli___6</td><td>Breast implants</td></tr><tr><td>7</td><td>pa_themecli___7</td><td>Hormonal issues</td></tr><tr><td>8</td><td>pa_themecli___8</td><td>Reproductive issues</td></tr><tr><td>9</td><td>pa_themecli___9</td><td>Urinary and erectile dysfunction issues</td></tr><tr><td>10</td><td>pa_themecli___10</td><td>Fatigue following treatments</td></tr><tr><td>11</td><td>pa_themecli___11</td><td>Pain and discomfort after treatment</td></tr><tr><td>12</td><td>pa_themecli___12</td><td>Pain and discomfort after surgery</td></tr><tr><td>13</td><td>pa_themecli___13</td><td>Possible emotions</td></tr><tr><td>14</td><td>pa_themecli___14</td><td>Stress and anxiety management</td></tr><tr><td>15</td><td>pa_themecli___15</td><td>Impact on physical appearance and self-esteem</td></tr><tr><td>16</td><td>pa_themecli___16</td><td>Decision-making processes</td></tr><tr><td>17</td><td>pa_themecli___17</td><td>Other information</td></tr></table>
Alignement personnalisé : LV</td></tr>
<tr><td></td><td>74</td><td>pa_aspclin_autre

Afficher le champ UNIQUEM ENT si :
[pa_themecli(17)] = '1'</td><td>If other information, please specify which one:</td><td>text
Alignement personnalisé : LV</td></tr>
</table>

	75	pa_themequo<br><br>Afficher le champ UNIQUEM ENT si :<br>[pa_jumelage] = '0'	Consequences on daily life	checkbox<br>0 | pa_themequo___0 | Consequences on daily life<br>1 | pa_themequo___1 | Consequences on children<br>2 | pa_themequo___2 | Consequences on conjugal life<br>3 | pa_themequo___3 | Consequences on sexual life<br>4 | pa_themequo___4 | Returning to work<br>5 | pa_themequo___5 | The return to daily life<br>6 | pa_themequo___6 | The consequences on the spiritual life<br>7 | pa_themequo___7 | How to announce a diagnosis to your loved ones<br>8 | pa_themequo___8 | Financial implications<br>9 | pa_themequo___9 | Consequences on insurance<br>10 | pa_themequo___10 | Consequences on professional life<br>11 | pa_themequo___11 | Social perception (the reaction of others)<br>12 | pa_themequo___12 | Strategies for living the treatments in the best conditions<br>13 | pa_themequo___13 | How to regain control over the disease<br>14 | pa_themequo___14 | Other information<br><br>Alignement personnalisé : LV
	76	pa_asp_viequot_autre<br><br>Afficher le champ UNIQUEM ENT si :<br>[pa_themequo(14)] = '1'	If other information, please specify which one:	text<br>Alignement personnalisé : LV
	77	pa_bene<br><br>Afficher le champ UNIQUEM ENT si :<br>[pa_jumelage] = '0'	In what area has the accompaniment been beneficial? (You may check more than one answer.)	checkbox<br>0 | pa_bene___0 | My understanding of my care pathway within the health care institution (steps, role of each person...)<br>1 | pa_bene___1 | My relationships and exchanges with the medical and nursing teams<br>2 | pa_bene___2 | My experience of the disease (sharing experiences on side effects, on the impact of the disease on daily life, with those around me...)<br>3 | pa_bene___3 | My quality of life (decrease in stress level, anxiety, feeling understood...)<br>4 | pa_bene___4 | My knowledge of services and associations that can help me.<br>5 | pa_bene___5 | This accompaniment has not been particularly beneficial to me.<br>6 | pa_bene___6 | Other<br><br>Alignement personnalisé : LV
	78	pa_bene_autre<br><br>Afficher le champ UNIQUEM ENT si :<br>[pa_bene(6)] = '1'	If other benefit, please specify which one:	text<br>Alignement personnalisé : LV

	79	pa_contri<br>Afficher le champ UNIQUEMENT si :<br>[pa_jumelage] = '0'	In your opinion, what is the contribution of the patient advisor? You may check more than one answer.	checkbox<br>1 pa_contri___1 Giving information<br>2 pa_contri___2 Giving support<br>3 pa_contri___3 Sharing experiences<br>4 pa_contri___4 Facilitate communication between the health care team and the patient being accompanied.<br>5 pa_contri___5 Helping to bring the patient's perspective to the health care team<br>6 pa_contri___6 Improving understanding of information shared between patient and health care team<br>7 pa_contri___7 Helping to remember information<br>8 pa_contri___8 Preparing the patient for medical appointments<br>9 pa_contri___9 Accompanying patients to medical appointments<br>10 pa_contri___10 Helping the patient participate in decision making<br>11 pa_contri___11 Bringing a new perspective to decision making<br>12 pa_contri___12 Referring patients to a variety of resources<br>13 pa_contri___13 Help develop a trusting relationship between the patient and the health care team<br>14 pa_contri___14 Helping the patient become a partner in his or her own care<br>15 pa_contri___15 Listening to the patient<br>16 pa_contri___16 Other contribution<br>17 pa_contri___17 No, the patient advisor has no particular contribution to make to the care trajectory.<br>Alignement personnalisé : LV
	80	pa_contriautre<br>Afficher le champ UNIQUEMENT si :<br>[pa_jumelage] = '0' and [pa_contri(16)] = '1'	Please indicate what other contribution the patient advisor has had.	text<br>Alignement personnalisé : LV
	81	pa_postacc_mat<br>Afficher le champ UNIQUEMENT si :<br>[pa_jumelage] = '0'	To what extent do you agree with each of the following statements?	descriptive
	82	mat_post_bes<br>Afficher le champ UNIQUEMENT si :<br>[pa_jumelage] = '0'	This/these meeting(s) with a patient advisor has/have met my needs:	radio (Matrice)<br>1 Strongly Disagree<br>2 Disagree<br>3 Neither agree nor disagree<br>4 Agree<br>5 Strongly agree<br>6 I don't know
	83	mat_post_lib<br>Afficher le champ UNIQUEMENT si :<br>[pa_jumelage] = '0'	I feel free to discuss all the subjects that preoccupy me during this/these meeting(s) with a patient advisor:	radio (Matrice)<br>1 Strongly Disagree<br>2 Disagree<br>3 Neither agree nor disagree<br>4 Agree<br>5 Strongly agree<br>6 I don't know

	84	mat_post_complem<br>Afficher le champ UNIQUEMENT si :<br>[pa_jumelage] = '0'	This/these meeting(s) with a patient advisor is/are in complementarity with the interventions of my health professionals:	radio (Matrice)<br>1 Strongly Disagree<br>2 Disagree<br>3 Neither agree nor disagree<br>4 Agree<br>5 Strongly agree<br>6 I don't know
	85	pa_postacc_mat2<br>Afficher le champ UNIQUEMENT si :<br>[pa_jumelage] = '0'	How satisfied are you with the following?	descriptive
	86	mat_post_freq<br>Afficher le champ UNIQUEMENT si :<br>[pa_jumelage] = '0'	The frequency of your exchanges with the patient advisor	radio (Matrice)<br>1 Very dissatisfied<br>2 Somewhat dissatisfied<br>3 Neither satisfied nor dissatisfied<br>4 Somewhat satisfied<br>5 Very satisfied<br>6 Not applicable
	87	mat_post_dur<br>Afficher le champ UNIQUEMENT si :<br>[pa_jumelage] = '0'	The duration of your exchanges with the patient advisor	radio (Matrice)<br>1 Very dissatisfied<br>2 Somewhat dissatisfied<br>3 Neither satisfied nor dissatisfied<br>4 Somewhat satisfied<br>5 Very satisfied<br>6 Not applicable
	88	mat_post_adapt<br>Afficher le champ UNIQUEMENT si :<br>[pa_jumelage] = '0'	The adaptation of physical spaces to exchanges (confidentiality...)	radio (Matrice)<br>1 Very dissatisfied<br>2 Somewhat dissatisfied<br>3 Neither satisfied nor dissatisfied<br>4 Somewhat satisfied<br>5 Very satisfied<br>6 Not applicable
	89	mat_post_facil<br>Afficher le champ UNIQUEMENT si :<br>[pa_jumelage] = '0'	Ease of access to the patient advisor when needed	radio (Matrice)<br>1 Very dissatisfied<br>2 Somewhat dissatisfied<br>3 Neither satisfied nor dissatisfied<br>4 Somewhat satisfied<br>5 Very satisfied<br>6 Not applicable
	90	pa_recom<br>Afficher le champ UNIQUEMENT si :<br>[pa_jumelage] = '0'	In the end, do you think this type of meeting would be interesting to deploy to help more patients and caregivers?	radio<br>1 Yes, absolutely<br>2 Yes, but by reviewing some of the terms and conditions.<br>3 Rather not, it doesn't really matter<br>4 No, not at all
	91	pa_recom_precis<br>Afficher le champ UNIQUEMENT si :<br>[pa_recom] = '2'	Please specify these terms and conditions to be reviewed	text
	92	pa_bidon3_v2_v2<br>Afficher le champ UNIQUEMENT si :<br>[pa_jumelage] = '1'	What topics would you like to discuss with a patient advisor? You can check more than one answer.	descriptive

<table>
<tr>
<td></td>
<td>93</td>
<td>pa_orgpo

Afficher le champ UNIQUEMENT si :
[pa_jumelage] = '1'</td>
<td>Organizational aspects</td>
<td>checkbox
<table>
<tr><td>0</td><td>pa_orgpo___0</td><td>Her/His role as a patient advisor</td></tr>
<tr><td>1</td><td>pa_orgpo___1</td><td>The role of different health care professionals</td></tr>
<tr><td>2</td><td>pa_orgpo___2</td><td>The role of external and internal organizations</td></tr>
<tr><td>3</td><td>pa_orgpo___3</td><td>The care trajectory</td></tr>
<tr><td>4</td><td>pa_orgpo___4</td><td>Rights as a patient (e.g., refusing treatment, asking questions)</td></tr>
<tr><td>5</td><td>pa_orgpo___5</td><td>Where and how to get to medical appointments</td></tr>
<tr><td>6</td><td>pa_orgpo___6</td><td>Financial support for patients and transportation assistance</td></tr>
<tr><td>7</td><td>pa_orgpo___7</td><td>The Parole-Onco Research Project</td></tr>
<tr><td>8</td><td>pa_orgpo___8</td><td>Other information</td></tr>
</table>
Alignement personnalisé : LV</td>
</tr>
<tr>
<td></td>
<td>94</td>
<td>pa_asporg2_autre

Afficher le champ UNIQUEMENT si :
[pa_orgpo(8)] = '1'</td>
<td>If other information, please specify which one:</td>
<td>text
Alignement personnalisé : LV</td>
</tr>
<tr>
<td></td>
<td>95</td>
<td>pa_ppaclipo_v2_v2

Afficher le champ UNIQUEMENT si :
[pa_jumelage] = '1'</td>
<td>Clinical aspects</td>
<td>checkbox
<table>
<tr><td>0</td><td>pa_ppaclipo_v2_v2___0</td><td>The announcement of the cancer diagnosis or genetic predisposition by the doctor</td></tr>
<tr><td>1</td><td>pa_ppaclipo_v2_v2___1</td><td>Cancer</td></tr>
<tr><td>2</td><td>pa_ppaclipo_v2_v2___2</td><td>Genetic testing</td></tr>
<tr><td>3</td><td>pa_ppaclipo_v2_v2___3</td><td>Therapeutic options in the case of cancer</td></tr>
<tr><td>4</td><td>pa_ppaclipo_v2_v2___4</td><td>Ways to reduce risk in carriers of a gene mutation that increases the risk of cancer</td></tr>
<tr><td>5</td><td>pa_ppaclipo_v2_v2___5</td><td>Surgical and reconstructive options for cancer</td></tr>
<tr><td>6</td><td>pa_ppaclipo_v2_v2___6</td><td>Breast implants</td></tr>
<tr><td>7</td><td>pa_ppaclipo_v2_v2___7</td><td>Hormonal issues</td></tr>
<tr><td>8</td><td>pa_ppaclipo_v2_v2___8</td><td>Reproductive issues</td></tr>
<tr><td>9</td><td>pa_ppaclipo_v2_v2___9</td><td>Urinary and erectile dysfunction issues</td></tr>
<tr><td>10</td><td>pa_ppaclipo_v2_v2___10</td><td>Fatigue following treatments</td></tr>
<tr><td>11</td><td>pa_ppaclipo_v2_v2___11</td><td>Pain and discomfort after treatment</td></tr>
<tr><td>12</td><td>pa_ppaclipo_v2_v2___12</td><td>Pain and discomfort after surgery</td></tr>
<tr><td>13</td><td>pa_ppaclipo_v2_v2___13</td><td>Possible emotions</td></tr>
<tr><td>14</td><td>pa_ppaclipo_v2_v2___14</td><td>Stress and anxiety management</td></tr>
<tr><td>15</td><td>pa_ppaclipo_v2_v2___15</td><td>Impact on physical appearance and self-esteem</td></tr>
<tr><td>16</td><td>pa_ppaclipo_v2_v2___16</td><td>Decision-making processes</td></tr>
<tr><td>17</td><td>pa_ppaclipo_v2_v2___17</td><td>Other information</td></tr>
</table>
Alignement personnalisé : LV</td>
</tr>
</table>

	96	pa_apsclin2_autre<br><br>Afficher le champ UNIQUEMENT si :<br>[pa_ppaclipo_v2_v2(17)] = '1'	If other information, please specify which one:	text<br>Alignement personnalisé : LV
	97	pa_quopo<br><br>Afficher le champ UNIQUEMENT si :<br>[pa_jumelage] = '1'	Consequences on daily life	checkbox<br>1 pa_quopo___1 Consequences on daily life<br>2 pa_quopo___2 Consequences on children<br>3 pa_quopo___3 Consequences on conjugal life<br>4 pa_quopo___4 Consequences on sexual life<br>5 pa_quopo___5 Returning to work<br>6 pa_quopo___6 The return to daily life<br>7 pa_quopo___7 The consequences on the spiritual life<br>8 pa_quopo___8 How to announce a diagnosis to your loved ones<br>9 pa_quopo___9 Financial implications<br>10 pa_quopo___10 Consequences on insurance<br>11 pa_quopo___11 Consequences on professional life<br>12 pa_quopo___12 Social perception (the reaction of others)<br>13 pa_quopo___13 Strategies for living the treatments in the best conditions<br>14 pa_quopo___14 How to regain control over the disease<br>15 pa_quopo___15 Other information<br><br>Alignement personnalisé : LV
	98	pa_ppaquopoautre_v2_v2<br><br>Afficher le champ UNIQUEMENT si :<br>[pa_quopo(15)] = '1'	If other information, please specify which one:	text<br>Alignement personnalisé : LV

<table>
<tr><td></td><td>99</td><td>pa_contripo

Afficher le champ UNIQUEMENT si :
[pa_jumelage] = '1' or [pa_jumelage] = '2'</td><td>In your opinion, what could be the contribution of the patient advisor?</td><td>checkbox
<table><tr><td>1</td><td>pa_contripo___1</td><td>Giving information</td></tr><tr><td>2</td><td>pa_contripo___2</td><td>Giving support</td></tr><tr><td>3</td><td>pa_contripo___3</td><td>Sharing experiences</td></tr><tr><td>4</td><td>pa_contripo___4</td><td>Facilitate communication between the health care team and the patient being accompanied.</td></tr><tr><td>5</td><td>pa_contripo___5</td><td>Helping to bring the patient's perspective to the health care team</td></tr><tr><td>6</td><td>pa_contripo___6</td><td>Improving understanding of information shared between patient and health care team</td></tr><tr><td>7</td><td>pa_contripo___7</td><td>Helping to remember information</td></tr><tr><td>8</td><td>pa_contripo___8</td><td>Preparing the patient for medical appointments</td></tr><tr><td>9</td><td>pa_contripo___9</td><td>Accompanying patients to medical appointments</td></tr><tr><td>10</td><td>pa_contripo___10</td><td>Helping the patient participate in decision making</td></tr><tr><td>11</td><td>pa_contripo___11</td><td>Bringing a new perspective to decision making</td></tr><tr><td>12</td><td>pa_contripo___12</td><td>Referring patients to a variety of resources</td></tr><tr><td>13</td><td>pa_contripo___13</td><td>Help develop a trusting relationship between the patient and the health care team</td></tr><tr><td>14</td><td>pa_contripo___14</td><td>Helping the patient become a partner in his or her own care</td></tr><tr><td>15</td><td>pa_contripo___15</td><td>Listening to the patient</td></tr><tr><td>16</td><td>pa_contripo___16</td><td>Other contribution</td></tr><tr><td>17</td><td>pa_contripo___17</td><td>No, the patient advisor has no particular contribution to make to the care trajectory.</td></tr></table>
Alignement personnalisé : LV</td></tr>
<tr><td></td><td>100</td><td>pa_ppacontripoautre_v2_v2

Afficher le champ UNIQUEMENT si :
[pa_contripo(16)] = '1'</td><td>Please indicate what other contribution the patient advisor could make.</td><td>text
Alignement personnalisé : LV</td></tr>
<tr><td></td><td>101</td><td>patients_experience_with_pas_questionnaire_complete</td><td>En-tête de section : Form Status
Complete?</td><td>dropdown
<table><tr><td>0</td><td>Incomplete</td></tr><tr><td>1</td><td>Unverified</td></tr><tr><td>2</td><td>Complete</td></tr></table></td></tr>
<tr><td colspan="5">Formulaire : Patient's experience with PAs questionnaire-T2 (patients_experience_with_pas_questionnairet2) Collapse</td></tr>
<tr><td></td><td>102</td><td>pa_jumelage_v2</td><td>Have you ever been accompanied by someone (this may be one or more meetings) who has had the same experience as you (cancer, surgery or genetic testing experience)? In health care settings, these people are called "patients advisors".</td><td>radio
<table><tr><td>0</td><td>Yes</td></tr><tr><td>1</td><td>Not yet but I wish to benefit from it</td></tr><tr><td>2</td><td>No and I don't think I need it</td></tr></table>
Alignement personnalisé : LV</td></tr>
<tr><td></td><td>103</td><td>pa_nbre_renc

Afficher le champ UNIQUEMENT si :
[pa_jumelage_v2] = '0'</td><td>How many meetings have you had with a patient advisor?</td><td>radio
<table><tr><td>1</td><td>Only one</td></tr><tr><td>2</td><td>Several</td></tr></table></td></tr>
</table>

	104	bidon_65<br>Afficher le champ UNIQUEMENT si :<br>[pa_nbre_renc] = '1' or [pa_jumelage_v2] = '1' or [pa_jumelage_v2] = '2'	Since you have had only one meeting with a patient advisor or have not yet met one, you do not have to answer this questionnaire. Thank you for your participation.	descriptive
	105	bidon_66<br>Afficher le champ UNIQUEMENT si :<br>[pa_jumelage_v2] = '0' and [pa_nbre_renc] = '2'	The following questions focus on your evaluation of the second (and subsequent if applicable) meeting with a patient advisor.	descriptive
	106	pa_bidon2_v2_v2_v2<br>Afficher le champ UNIQUEMENT si :<br>[pa_jumelage_v2] = '0' and [pa_nbre_renc] = '2'	What topics did you discuss with the patient advisor? (Check all that apply)	descriptive
	107	pa_themeorg_v2<br>Afficher le champ UNIQUEMENT si :<br>[pa_jumelage_v2] = '0' and [pa_nbre_renc] = '2'	Organizational aspects	checkbox<br>0 | pa_themeorg_v2___0 | Her/His role as a patient advisor<br>1 | pa_themeorg_v2___1 | The role of different health care professionals<br>2 | pa_themeorg_v2___2 | The role of external and internal organizations<br>3 | pa_themeorg_v2___3 | The care trajectory<br>4 | pa_themeorg_v2___4 | Rights as a patient (e.g., refusing treatment, asking questions)<br>5 | pa_themeorg_v2___5 | Where and how to get to medical appointments<br>6 | pa_themeorg_v2___6 | Financial support for patients and transportation assistance<br>7 | pa_themeorg_v2___7 | The Parole-Onco Research Project<br>8 | pa_themeorg_v2___8 | Other information<br>Alignement personnalisé : LV
	108	pa_asp_org_autre_v2<br>Afficher le champ UNIQUEMENT si :<br>[pa_themeorg_v2(8)] = '1'	If other information, please specify which one:	text<br>Alignement personnalisé : LV

	109	pa_themecli_v2<br><br>Afficher le champ UNIQUEMENT si :<br>[pa_jumelage_v2] = '0' and [pa_nbre_renc] = '2'	Clinical aspects	checkbox<br>0 | pa_themecli_v2___0 | The announcement of the cancer diagnosis or genetic predisposition by the doctor<br>1 | pa_themecli_v2___1 | Cancer<br>2 | pa_themecli_v2___2 | Genetic testing<br>3 | pa_themecli_v2___3 | Therapeutic options in the case of cancer<br>4 | pa_themecli_v2___4 | Ways to reduce risk in carriers of a gene mutation that increases the risk of cancer<br>5 | pa_themecli_v2___5 | Surgical and reconstructive options for cancer<br>6 | pa_themecli_v2___6 | Breast implants<br>7 | pa_themecli_v2___7 | Hormonal issues<br>8 | pa_themecli_v2___8 | Reproductive issues<br>9 | pa_themecli_v2___9 | Urinary and erectile dysfunction issues<br>10 | pa_themecli_v2___10 | Fatigue following treatments<br>11 | pa_themecli_v2___11 | Pain and discomfort after treatment<br>12 | pa_themecli_v2___12 | Pain and discomfort after surgery<br>13 | pa_themecli_v2___13 | Possible emotions<br>14 | pa_themecli_v2___14 | Stress and anxiety management<br>15 | pa_themecli_v2___15 | Impact on physical appearance and self-esteem<br>16 | pa_themecli_v2___16 | Decision-making processes<br>17 | pa_themecli_v2___17 | Other information<br><br>Alignement personnalisé : LV
	110	pa_aspclin_autre_v2<br><br>Afficher le champ UNIQUEMENT si :<br>[pa_themecli_v2(17)] = '1'	If other information, please specify which one:	text<br>Alignement personnalisé : LV

	111	pa_themequo_v2<br><br>Afficher le champ UNIQUEMENT si :<br>[pa_jumelage_v2] = '0' and [pa_nbre_renc] = '2'	Consequences on daily life	checkbox<br>0 - pa_themequo_v2___0 - Consequences on daily life<br>1 - pa_themequo_v2___1 - Consequences on children<br>2 - pa_themequo_v2___2 - Consequences on conjugal life<br>3 - pa_themequo_v2___3 - Consequences on sexual life<br>4 - pa_themequo_v2___4 - Returning to work<br>5 - pa_themequo_v2___5 - The return to daily life<br>6 - pa_themequo_v2___6 - The consequences on the spiritual life<br>7 - pa_themequo_v2___7 - How to announce a diagnosis to your loved ones<br>8 - pa_themequo_v2___8 - Financial implications<br>9 - pa_themequo_v2___9 - Consequences on insurance<br>10 - pa_themequo_v2___10 - Consequences on professional life<br>11 - pa_themequo_v2___11 - Social perception (the reaction of others)<br>12 - pa_themequo_v2___12 - Strategies for living the treatments in the best conditions<br>13 - pa_themequo_v2___13 - How to regain control over the disease<br>14 - pa_themequo_v2___14 - Other information<br><br>Alignement personnalisé : LV
	112	pa_asp_viequot_autre_v2<br><br>Afficher le champ UNIQUEMENT si :<br>[pa_themequo_v2(14)] = '1'	If other information, please specify which one:	text<br>Alignement personnalisé : LV
	113	pa_bene_v2<br><br>Afficher le champ UNIQUEMENT si :<br>[pa_jumelage_v2] = '0' and [pa_nbre_renc] = '2'	In what area has the accompaniment been beneficial? (You may check more than one answer.)	checkbox<br>0 - pa_bene_v2___0 - My understanding of my care pathway within the health care institution (steps, role of each person...)<br>1 - pa_bene_v2___1 - My relationships and exchanges with the medical and nursing teams<br>2 - pa_bene_v2___2 - My experience of the disease (sharing experiences on side effects, on the impact of the disease on daily life, with those around me...)<br>3 - pa_bene_v2___3 - My quality of life (decrease in stress level, anxiety, feeling understood...)<br>4 - pa_bene_v2___4 - My knowledge of services and associations that can help me.<br>5 - pa_bene_v2___5 - This accompaniment has not been particularly beneficial to me.<br>6 - pa_bene_v2___6 - Other<br><br>Alignement personnalisé : LV
	114	pa_bene_autre_v2<br><br>Afficher le champ UNIQUEMENT si :<br>[pa_bene_v2(6)] = '1'	If other benefit, please specify which one:	text<br>Alignement personnalisé : LV

	115	pa_contri_v2<br><br>Afficher le champ UNIQUEM ENT si :<br>[pa_jumelage_v2] = '0' and [pa_nbre_renc] = '2'	In your opinion, what is the contribution of the patient advisor? You may check more than one answer.	checkbox<br>1 pa_contri_v2___1 Giving information<br>2 pa_contri_v2___2 Giving support<br>3 pa_contri_v2___3 Sharing experiences<br>4 pa_contri_v2___4 Facilitate communication between the health care team and the patient being accompanied.<br>5 pa_contri_v2___5 Helping to bring the patient's perspective to the health care team<br>6 pa_contri_v2___6 Improving understanding of information shared between patient and health care team<br>7 pa_contri_v2___7 Helping to remember information<br>8 pa_contri_v2___8 Preparing the patient for medical appointments<br>9 pa_contri_v2___9 Accompanying patients to medical appointments<br>10 pa_contri_v2___10 Helping the patient participate in decision making<br>11 pa_contri_v2___11 Bringing a new perspective to decision making<br>12 pa_contri_v2___12 Referring patients to a variety of resources<br>13 pa_contri_v2___13 Help develop a trusting relationship between the patient and the health care team<br>14 pa_contri_v2___14 Helping the patient become a partner in his or her own care<br>15 pa_contri_v2___15 Listening to the patient<br>16 pa_contri_v2___16 Other contribution<br>17 pa_contri_v2___17 No, the patient advisor has no particular contribution to make to the care trajectory.<br><br>Alignement personnalisé : LV
	116	pa_contriautre_v2<br><br>Afficher le champ UNIQUEM ENT si :<br>[pa_contri_v2(16)] = '1'	Please indicate what other contribution the patient advisor made.	text<br>Alignement personnalisé : LV
	117	pa_postacc_mat_v2<br><br>Afficher le champ UNIQUEM ENT si :<br>[pa_jumelage_v2] = '0' and [pa_nbre_renc] = '2'	To what extent do you agree with each of the following statements?	descriptive
	118	mat_post_bes_v2<br><br>Afficher le champ UNIQUEM ENT si :<br>[pa_jumelage_v2] = '0' and [pa_nbre_renc] = '2'	This/these meeting(s) with a patient advisor met my needs:	radio (Matrice)<br>1 Strongly Disagree<br>2 Disagree<br>3 Neither agree nor disagree<br>4 Agree<br>5 Strongly agree<br>6 I don't know

	119	mat_post_lib_v2<br>Afficher le champ UNIQUEMENT si :<br>[pa_jumelage_v2] = '0' and [pa_nbre_renc] = '2'	I feel free to discuss all the subjects that preoccupy me during this/these meeting(s) with a patient advisor:	radio (Matrice)<br>1 Strongly Disagree<br>2 Disagree<br>3 Neither agree nor disagree<br>4 Agree<br>5 Strongly agree<br>6 I don't know
	120	mat_post_complem_v2<br>Afficher le champ UNIQUEMENT si :<br>[pa_jumelage_v2] = '0' and [pa_nbre_renc] = '2'	This/these meeting(s) with a patient advisor is/are in complementarity with the interventions of my health professionals:	radio (Matrice)<br>1 Strongly Disagree<br>2 Disagree<br>3 Neither agree nor disagree<br>4 Agree<br>5 Strongly agree<br>6 I don't know
	121	pa_postacc_mat2_v2<br>Afficher le champ UNIQUEMENT si :<br>[pa_jumelage_v2] = '0' and [pa_nbre_renc] = '2'	How satisfied are you with the following?	descriptive
	122	mat_post_freq_v2<br>Afficher le champ UNIQUEMENT si :<br>[pa_jumelage_v2] = '0' and [pa_nbre_renc] = '2'	The frequency of your exchanges with the patient advisor	radio (Matrice)<br>1 Very dissatisfied<br>2 Somewhat dissatisfied<br>3 Neither satisfied nor dissatisfied<br>4 Somewhat satisfied<br>5 Very satisfied<br>6 Not applicable
	123	mat_post_dur_v2<br>Afficher le champ UNIQUEMENT si :<br>[pa_jumelage_v2] = '0' and [pa_nbre_renc] = '2'	The duration of your exchanges with the patient advisor	radio (Matrice)<br>1 Very dissatisfied<br>2 Somewhat dissatisfied<br>3 Neither satisfied nor dissatisfied<br>4 Somewhat satisfied<br>5 Very satisfied<br>6 Not applicable
	124	mat_post_adapt_v2<br>Afficher le champ UNIQUEMENT si :<br>[pa_jumelage_v2] = '0' and [pa_nbre_renc] = '2'	The adaptation of physical spaces to exchanges (confidentiality...)	radio (Matrice)<br>1 Very dissatisfied<br>2 Somewhat dissatisfied<br>3 Neither satisfied nor dissatisfied<br>4 Somewhat satisfied<br>5 Very satisfied<br>6 Not applicable
	125	mat_post_facil_v2<br>Afficher le champ UNIQUEMENT si :<br>[pa_jumelage_v2] = '0' and [pa_nbre_renc] = '2'	Ease of access to the patient advisor when needed.	radio (Matrice)<br>1 Very dissatisfied<br>2 Somewhat dissatisfied<br>3 Neither satisfied nor dissatisfied<br>4 Somewhat satisfied<br>5 Very satisfied<br>6 Not applicable
	126	pa_recom_v2<br>Afficher le champ UNIQUEMENT si :<br>[pa_jumelage_v2] = '0' and [pa_nbre_renc] = '2'	In the end, do you think this type of meeting would be interesting to deploy to help more patients and caregivers?	radio<br>1 Yes, absolutely<br>2 Yes, but by reviewing some of the terms and conditions.<br>3 Rather not, it doesn't really matter<br>4 No, not at all

	127	pa_recom_precis_v2<br><br>Afficher le champ UNIQUEM ENT si :<br>[pa_recom_v2] = '2'	Please specify these terms and conditions to be reviewed	text
	128	patients_experience_with_pas_questionnairet2_complete	En-tête de section : *Form Status*<br><br>Complete?	dropdown<br>0 Incomplete<br>1 Unverified<br>2 Complete
Formulaire : **CASE and K6** (case_and_k6)				Collapse
	129	pa_caseface_v2_v2	En-tête de section : *ABILITY TO COPE WITH CANCER Please indicate the extent to which you disagree or agree with the following statements by checking the answer that best describes you at this time.*<br><br>I am confident that I can deal with any unexpected health problems.	radio (Matrice)<br>1 Strongly Disagree<br>2 Disagree<br>3 Agree<br>4 Strongly agree
	130	pa_caseecrit_v2_v2	I am confident in my ability to understand written information about cancer.	radio (Matrice)<br>1 Strongly Disagree<br>2 Disagree<br>3 Agree<br>4 Strongly agree
	131	pa_casemd_v2_v2	I am confident in my ability to understand the instructions of my doctor(s).	radio (Matrice)<br>1 Strongly Disagree<br>2 Disagree<br>3 Agree<br>4 Strongly agree
	132	pa_caseparti_v2_v2	I know that I will be able to actively participate in decisions about my treatments.	radio (Matrice)<br>1 Strongly Disagree<br>2 Disagree<br>3 Agree<br>4 Strongly agree
	133	pa_casedecou_v2_v2	I will not be discouraged by cancer.	radio (Matrice)<br>1 Strongly Disagree<br>2 Disagree<br>3 Agree<br>4 Strongly agree
	134	pa_caseatti_v2_v2	It's easy for me to keep a positive attitude.	radio (Matrice)<br>1 Strongly Disagree<br>2 Disagree<br>3 Agree<br>4 Strongly agree
	135	pa_casehumou_v2_v2	It's easy for me to keep a sense of humor.	radio (Matrice)<br>1 Strongly Disagree<br>2 Disagree<br>3 Agree<br>4 Strongly agree
	136	pa_casesenti_v2_v2	I feel confident that I can control my negative feelings about cancer.	radio (Matrice)<br>1 Strongly Disagree<br>2 Disagree<br>3 Agree<br>4 Strongly agree

	137	pa_casedeman_v2_v2	If I don't understand something, it's easy for me to ask for help.	radio (Matrice)<br>1 Strongly Disagree<br>2 Disagree<br>3 Agree<br>4 Strongly agree
	138	pa_caseposer_v2_v2	It's easy for me to ask the nurse(s) questions.	radio (Matrice)<br>1 Strongly Disagree<br>2 Disagree<br>3 Agree<br>4 Strongly agree
	139	pa_caseques_v2_v2	It's easy for me to ask my doctor(s) questions.	radio (Matrice)<br>1 Strongly Disagree<br>2 Disagree<br>3 Agree<br>4 Strongly agree
	140	pa_caseobte_v2_v2	It's easy for me to get information about cancer.	radio (Matrice)<br>1 Strongly Disagree<br>2 Disagree<br>3 Agree<br>4 Strongly agree
	141	pa_k6nerv_v2_v2	En-tête de section : *YOUR EMOTIONAL STATE*<br>In the last 30 days, how often did you feel nervous?	radio (Matrice)<br>1 All of the time<br>2 Most of the time<br>3 Some of the time<br>4 A little of the time<br>5 None of the time
	142	pa_k6deses_v2_v2	In the past 30 days, how often did you feel hopeless?	radio (Matrice)<br>1 All of the time<br>2 Most of the time<br>3 Some of the time<br>4 A little of the time<br>5 None of the time
	143	pa_k6agite_v2_v2	In the past 30 days, how often did you feel restless or fidgety?	radio (Matrice)<br>1 All of the time<br>2 Most of the time<br>3 Some of the time<br>4 A little of the time<br>5 None of the time
	144	pa_k6sourire_v2_v2	In the past 30 days, how often did you feel so depressed that nothing could cheer you up?	radio (Matrice)<br>1 All of the time<br>2 Most of the time<br>3 Some of the time<br>4 A little of the time<br>5 None of the time
	145	pa_k6effort_v2_v2	In the last 30 days, how often did you feel everything was an effort?	radio (Matrice)<br>1 All of the time<br>2 Most of the time<br>3 Some of the time<br>4 A little of the time<br>5 None of the time

	146	pa_k6rien_v2_v2	In the last 30 days, how often did you feel worthless?	radio (Matrice)<br>1 All of the time<br>2 Most of the time<br>3 Some of the time<br>4 A little of the time<br>5 None of the time
	147	case_and_k6_complete	En-tête de section : *Form Status*<br>Complete?	dropdown<br>0 Incomplete<br>1 Unverified<br>2 Complete

Formulaire : **Treatment adherence questionnaire** (treatment_adherence_questionnaire) Collapse

	148	pa_traitement	At the time of completing this questionnaire, which stage best describes where you are in your cancer care?	radio<br>1 Before starting treatment<br>2 Treatement in progress<br>3 Treatment completed<br><br>Alignement personnalisé : LV
	149	bidon54<br><br>Afficher le champ UNIQUEMENT si :<br>[pa_traitement] = '1' OR [pa_traitement] = '3'	Since you are not currently undergoing treatment, you do not have to answer this questionnaire. Thank you for your participation.	descriptive
	150	bidon_55<br><br>Afficher le champ UNIQUEMENT si :<br>[pa_traitement] = '2'	YOUR ADHERENCE TO YOUR TREATMENTS<br><br>The questions below relate to your treatments.<br>Please indicate the extent to which you disagree or agree with the following statements by checking the answer that best describes you at this time.	descriptive
	151	pa_adheenga2<br><br>Afficher le champ UNIQUEMENT si :<br>[pa_traitement] = '2'	I have made a commitment to follow my treatment plan.	radio (Matrice)<br>1 Strongly disagree<br>2 Disagree<br>3 Neither agree nor disagree<br>4 Agree<br>5 Strongly agree
	152	pa_adheplan2<br><br>Afficher le champ UNIQUEMENT si :<br>[pa_traitement] = '2'	Following my treatment plan is not in my plans.	radio (Matrice)<br>1 Strongly disagree<br>2 Disagree<br>3 Neither agree nor disagree<br>4 Agree<br>5 Strongly agree
	153	pa_adheinten2<br><br>Afficher le champ UNIQUEMENT si :<br>[pa_traitement] = '2'	I intend to follow my treatment plan.	radio (Matrice)<br>1 Strongly disagree<br>2 Disagree<br>3 Neither agree nor disagree<br>4 Agree<br>5 Strongly agree
	154	pa_adhepainten2<br><br>Afficher le champ UNIQUEMENT si :<br>[pa_traitement] = '2'	I have no intention of following my treatment plan.	radio (Matrice)<br>1 Strongly disagree<br>2 Disagree<br>3 Neither agree nor disagree<br>4 Agree<br>5 Strongly agree
	155	pa_bidon52<br><br>Afficher le champ UNIQUEMENT si :<br>[pa_traitement] = '2'	Please indicate the extent to which you disagree or agree with the following statements by checking the answer that best describes you at this time.	descriptive

	156	pa_adheempe2<br><br>Afficher le champ UNIQUEM ENT si :<br>[pa_traitement] = '2'	Lots of things get in the way of following my treatment plan.	radio (Matrice)<br>1 Strongly disagree<br>2 Disagree<br>3 Neither agree nor disagree<br>4 Agree<br>5 Strongly agree
	157	pa_adhebesoin2<br><br>Afficher le champ UNIQUEM ENT si :<br>[pa_traitement] = '2'	I need more assistance in order to follow my treatment plan.	radio (Matrice)<br>1 Strongly disagree<br>2 Disagree<br>3 Neither agree nor disagree<br>4 Agree<br>5 Strongly agree
	158	pa_adheaide2<br><br>Afficher le champ UNIQUEM ENT si :<br>[pa_traitement] = '2'	I get the help I need to carry out my treatment plan.	radio (Matrice)<br>1 Strongly disagree<br>2 Disagree<br>3 Neither agree nor disagree<br>4 Agree<br>5 Strongly agree
	159	pa_adhecapa2<br><br>Afficher le champ UNIQUEM ENT si :<br>[pa_traitement] = '2'	I am able to deal with any problems in following my treatment plan.	radio (Matrice)<br>1 Strongly disagree<br>2 Disagree<br>3 Neither agree nor disagree<br>4 Agree<br>5 Strongly agree
	160	treatment_adherence_questionnaire_complete	En-tête de section : *Form Status*<br>Complete?	dropdown<br>0 Incomplete<br>1 Unverified<br>2 Complete

Formulaire : **Accompaniment** (accompaniment) Expand

Formulaire : **Focus group** (focus_group) Expand

Formulaire : **Interview** (interview) Expand

Formulaire : **End-of-study status** (endofstudy_status) Expand

Formulaire : **Focus group general form** (focus_group_general_form) Expand
