# Supplementary material for: The patient advisor, an organizational resource as a lever for an enhanced oncology patient experience (PAROLE-onco): a longitudinal multiple case study protocol
Source: BMC Health Serv Res. 2021 Jan 4;21:10. doi: 10.1186/s12913-020-06009-4 (PMC7780212; doi:10.1186/s12913-020-06009-4)
Supplement: Supplementary file 5 — Additional file 5. PAROLE-Onco-2-Patient accompagnateur-English- Code du dictionnaire de données - Variable name, question formulation and response options for each question in the patients advisors questionnaires. [file 12913_2020_6009_MOESM5_ESM.pdf]

PAROLE-Onco-2-Patient accompagnateur-English

Guide des codes

Code du dictionnaire de données

09-10-2020 21:10

Collapse all instruments

	#	Nom de variable/champ	Étiquette de champ<br>*Note de champ*	Attributs de champ (type de champ, validation, choix, logique de branchement, calculs, etc.)
Formulaire : **Formulaire d'identification des participants** (formulaire_didentification_des_participants) Expand				
Formulaire : **Consentement abrégé** (consentement_abrg) Expand				
Formulaire : **PA-Socio-demographic questionnaire** (pasociodemographic_questionnaire) Collapse				
	18	pa_bidon6pa_v2	To begin the questionnaire, here are some socio-demographic questions.	descriptive
	19	pa_sexepa_v2	You are...?	radio<br>1 A woman<br>2 A man<br>Alignement personnalisé : LV
	20	pa_agepa_v2	What is your age group?	radio<br>1 24 years old and under<br>2 25-34 years old<br>3 35-44 years old<br>4 45-54 years old<br>5 55-64 years old<br>6 65-74 years old<br>7 75 years old and over<br>Alignement personnalisé : LV
	21	pa_nepa_v2	Where were you born?	radio<br>1 In Quebec<br>2 Elsewhere in Canada<br>3 Outside Canada<br>4 Does not answer<br>Alignement personnalisé : LV
	22	pa_neepascanadapa_v2<br>Afficher le champ UNIQUEMENT si :<br>[pa_nepa_v2] = '3'	If you were born outside of Canada, please specify the country.	text<br>Alignement personnalisé : LV
	23	pa_anqcpa_v2<br>Afficher le champ UNIQUEMENT si :<br>[pa_nepa_v2] = '2' or [pa_nepa_v2] = '3'	How many years have you lived in Quebec?	radio<br>1 Less than 5 years<br>2 5 to 10 years<br>3 More than 10 years<br>4 Don't know<br>Alignement personnalisé : LV

	24	ppa57pa_v2	In which administrative region do you live?	radio<br>0 Bas-Saint-Laurent<br>1 Saguenay-Lac-Saint-Jean<br>2 Capitale-Nationale (Québec)<br>3 Mauricie-et-Centre-du-Québec<br>4 Estrie<br>5 Montréal<br>6 Outaouais<br>7 Abitibi-Témiscamingue<br>8 Côte-Nord<br>9 Nord-du-Québec<br>10 Gaspésie-Îles-de-la-Madeleine<br>11 Chaudière-Appalaches<br>12 Laval<br>13 Lanaudière<br>14 Laurentides<br>15 Montérégie<br>16 Nunavik<br>17 Terres-Cries-de-la-Baie-James<br>Alignement personnalisé : LV
	25	pa_nbmenagepa_v2	How many people compose your household and reside permanently in your home, including you?	text (number)<br>Alignement personnalisé : LV
	26	pa_nbchargepa_v2	How many of the people in your household are dependent on you?	text (number)<br>Alignement personnalisé : LV
	27	pa_situationpa_v2	What type of situation best suits the composition of your household?	radio<br>1 Person living alone in the household<br>2 Couple without children at home<br>3 Couple with children at home<br>4 Single-parent family (children of any age)<br>5 Family with other people<br>6 Related persons only (siblings, etc.)<br>7 Non-related persons only (roommates)<br>8 Other (multiple families, etc.)<br>Alignement personnalisé : LV
	28	pa_situationautrepa_v2<br>Afficher le champ UNIQUEMENT si :<br>[pa_situationpa_v2] = '8'	If you answered "Other", please specify the composition of your household.	text<br>Alignement personnalisé : LV
	29	ppa_entou_v2_v2	Is there one or more people in your entourage (spouse, child, friend, other) who can help you as needed for your medical follow-up?	radio<br>1 Yes<br>2 No<br>Alignement personnalisé : LV
	30	ppa_entouprecis_v2_v2<br>Afficher le champ UNIQUEMENT si :<br>[ppa_entou_v2_v2] = '1'	This person is your (you can choose more than one answer) :	checkbox<br>1 ppa_entouprecis_v2_v2___1 Spouse<br>2 ppa_entouprecis_v2_v2___2 Child<br>3 ppa_entouprecis_v2_v2___3 Parent<br>4 ppa_entouprecis_v2_v2___4 Friend<br>5 ppa_entouprecis_v2_v2___5 Other<br>Alignement personnalisé : LV
	31	pro_entouautre_v2_v2<br>Afficher le champ UNIQUEMENT si :<br>[ppa_entouprecis_v2_v2(5)] = '1'	If you answered "Other", please specify:	text<br>Alignement personnalisé : LV

	32	pa_educpa_v2	What is the highest level of education you have completed?	radio<br>1 No diploma<br>2 Elementary education<br>3 Partial studies in High School (I-IV)<br>4 High School Diploma (Sec V or Grade 12)<br>5 Partial studies in a CEGEP, technical school or professional training school<br>6 Diploma or certificate from a CEGEP, technical school or professional training school<br>7 Partial studies at university<br>8 University degree<br>9 Other<br><br>Alignement personnalisé : LV
	33	pa_eduautrepa_v2<br>Afficher le champ UNIQUEMENT si :<br>[pa_educpa_v2] = '9'	If you checked "Other", please specify the highest level of education you have completed.	text<br>Alignement personnalisé : LV
	34	pa_occupa_v2	In the past 12 months, what was your main occupation?	radio<br>1 Full-time worker<br>2 Part-time worker<br>3 Student<br>4 Volunteer/Unpaid Worker<br>5 Retired<br>6 Homemaker<br>7 On maternity/paternity leave<br>8 Employment Insurance claimant<br>9 Income assistance recipient (income security)<br>10 Other<br><br>Alignement personnalisé : LV
	35	pa_occuautrepa_v2<br>Afficher le champ UNIQUEMENT si :<br>[pa_occupa_v2] = '10'	If you answered "Other", please specify what was your main occupation in the last 12 months.	text<br>Alignement personnalisé : LV
	36	pa_finanpa_v2	How do you feel about your financial situation compared to people your own age?	radio<br>1 You consider yourself financially comfortable<br>2 You consider that your income is sufficient to meet your basic needs or those of your family.<br>3 You consider yourself poor<br>4 You consider yourself very poor<br><br>Alignement personnalisé : LV
	37	pasociodemographic_questionnaire_complete	En-tête de section : *Form Status*<br>Complete?	dropdown<br>0 Incomplete<br>1 Unverified<br>2 Complete

Formulaire : **PA's care experience questionnaire** (pas_care_experience_questionnaire) Collapse

	38	intro	The following questions focus on your clinical experience and the information provided by your health care team during your own episode of care related to cancer or genetic predisposition to cancer.<br>By care team, we mean the various health care professionals working together: doctors, nurses, pivot nurses, pharmacists, nutritionists, psychologists, social workers, physiotherapists, etc...	descriptive

	39	ppa_recru	In which institution were you recruited for this project?	radio<br>0 At CHUM<br>1 At CIUSSS de la Mauricie-et-du-Centre-du-Québec<br>2 At CIUSSS de l'Est-de-l'Île-de-Montréal<br>3 At CHU de Québec-Université Laval<br>4 At CISSS de Laval<br>5 At CIUSS du Centre-Ouest-de-l'Ile-de-Montréal<br>6 At CISSS de Gaspésie<br>7 At CIUSSS de Saguenay-Lac-Saint-Jean<br>8 At CUSM<br><br>Alignement personnalisé : LV
	40	ppa_suivi	Your medical follow-up took place in the context of :	radio<br>1 An episode of cancer<br>2 A cancer episode that led to an oncogenetic consultation<br>3 A consultation in oncogenetic<br><br>Alignement personnalisé : LV
	41	ppa_cancer<br>Afficher le champ UNIQUEMENT si :<br>[ppa_suivi] = '1' or [ppa_suivi] = '2'	What is the main type of cancer you have been treated for?	radio<br>1 Oral cavity<br>2 Brain<br>3 Colorectal (colon, rectum)<br>4 Cervix<br>5 Endometrium / Uterus<br>6 Stomach / Esophagus<br>7 Liver<br>8 Leukemia<br>9 Non-Hodgkin's lymphoma<br>10 Melanoma<br>11 Ovary<br>12 Pancreas<br>13 Skin (without melanoma)<br>14 Lung / Bronchus<br>15 Prostate<br>16 Kidney<br>17 Breast<br>18 Thyroid<br>19 Bladder<br>20 Other cancer<br><br>Alignement personnalisé : LV
	42	ppa_cancerautre<br>Afficher le champ UNIQUEMENT si :<br>[ppa_cancer] = '20'	If other cancer, specify the type of cancer:	text<br>Alignement personnalisé : LV
	43	ppa_metasta<br>Afficher le champ UNIQUEMENT si :<br>[ppa_suivi] = '1' or [ppa_suivi] = '2'	Is it a metastatic cancer?	radio<br>0 No<br>1 Yes
	44	ppa_canceran<br>Afficher le champ UNIQUEMENT si :<br>[ppa_suivi] = '1' or [ppa_suivi] = '2'	In what year were you diagnosed with this cancer?	text (number, Min. : 1950, Max. : 2021)<br>Alignement personnalisé : LV

	45	ppa_trait<br>Afficher le champ UNIQUEMENT si :<br>[ppa_suivi] = '1' or [ppa_suivi] = '2'	What type of treatment or surgery have you received (check all that apply)?	checkbox<br>1 ppa_trait___1 Vein Chemotherapy (Intravenous)<br>2 ppa_trait___2 Chemotherapy with swallowable tablets<br>3 ppa_trait___3 Hormonal therapy (antihormones)<br>4 ppa_trait___4 Radiotherapy or brachytherapy<br>5 ppa_trait___5 Partial Surgery (remove the tumor while preserving as much healthy tissue as possible)<br>6 ppa_trait___6 Complete Surgery (completely remove the affected organ)<br>7 ppa_trait___7 Reconstructive surgery<br>8 ppa_trait___8 Transplantation or grafting<br>9 ppa_trait___9 Immunotherapy<br>10 ppa_trait___10 Alternative Medicine<br>11 ppa_trait___11 Don't know<br>12 ppa_trait___12 Other treatment<br>Alignement personnalisé : LV
	46	ppa_traitautre<br>Afficher le champ UNIQUEMENT si :<br>[ppa_trait(12)] = '1'	What other treatment have you received?	text<br>Alignement personnalisé : LV
	47	ppa_traitementfin<br>Afficher le champ UNIQUEMENT si :<br>[ppa_suivi] = '1' or [ppa_suivi] = '2'	Your last treatment ended :	radio<br>1 Less than one year ago<br>2 Between 1 and 3 years ago<br>3 Between 4 and 5 years ago<br>4 More than 5 years ago<br>5 Does not apply, I am still in treatment<br>6 Don't know<br>Alignement personnalisé : LV
	48	ppa_traitfin5<br>Afficher le champ UNIQUEMENT si :<br>[ppa_traitementfin] = '4'	If your last treatment was completed more than 5 years ago, please indicate the number of years	text<br>Alignement personnalisé : LV
	49	ppa_motifgen<br>Afficher le champ UNIQUEMENT si :<br>[ppa_suivi] = '2' or [ppa_suivi] = '3'	Why did you consult in oncogenetic OR genetic testing?	radio<br>1 Therapeutic decision (surgical decision or targeted therapy)<br>2 To find out my genetic status following a cancer diagnosis<br>3 To know my genetic status following the identification of a genetic mutation in the family<br>5 To know my genetic status due to my family history of cancer<br>Alignement personnalisé : LV
	50	ppa_etapegen<br>Afficher le champ UNIQUEMENT si :<br>[ppa_suivi] = '2' or [ppa_suivi] = '3'	At the time of answering this questionnaire, which step best describes where you are in your oncogenetic trajectory?	radio<br>1 You have just attended an information meeting in oncogenetics<br>2 You are waiting for the result of your genetic test<br>3 You are not a carrier of a genetic mutation<br>4 You are a carrier of a genetic mutation<br>5 You are a carrier of a genetic mutation and you have had preventive surgery on your breasts or ovaries.<br>Alignement personnalisé : LV

	51	ppa_qdresult<br><br>Afficher le champ UNIQUEM ENT si :<br>[ppa_etapegen] = '3' or [ppa_etapegen] = '4' or [ppa_etapegen] = '5'	You received your genetic test result :	radio<br>1 Less than one month ago<br>2 Between 1 and 6 months ago<br>3 Between 7 and 11 months ago<br>4 Between 1 and 2 years ago<br>5 Between 3 and 5 years ago<br>6 More than 5 years ago<br>7 Don't know<br><br>Alignement personnalisé : LV
	52	ppa_qdresult5<br><br>Afficher le champ UNIQUEM ENT si :<br>[ppa_qdresult] = '6'	If more than 5 years, please indicate the number of years that have elapsed since the release of your genetic test result.	text<br>Alignement personnalisé : LV
	53	ppa_mastect<br><br>Afficher le champ UNIQUEM ENT si :<br>[ppa_etapegen] = '4' or [ppa_etapegen] = '5'	Have you had or are you considering preventative mastectomy (breast removal) to reduce your risk of breast cancer?	radio<br>1 Yes, I had this surgery<br>2 Yes, I am considering this surgery<br>3 No, I have not had this surgery and I am not considering it.<br>4 Not applicable<br><br>Alignement personnalisé : LV
	54	ppa_mastectan<br><br>Afficher le champ UNIQUEM ENT si :<br>[ppa_mastect] = '1'	In what year did you have your preventive mastectomy?	text (number, Min. : 1950, Max. : 2021)<br>Alignement personnalisé : LV
	55	ppa_reconstruct<br><br>Afficher le champ UNIQUEM ENT si :<br>[ppa_mastect] = '1'	Have you had a breast reconstruction?	radio<br>1 Yes<br>2 No<br><br>Alignement personnalisé : LV
	56	ppa_typereconstruct<br><br>Afficher le champ UNIQUEM ENT si :<br>[ppa_reconstruct] = '1'	What type of breast reconstruction have you had?	radio<br>1 Reconstruction by adding breast implants<br>2 Reconstruction from your own tissues<br>3 Don't know<br><br>Alignement personnalisé : LV
	57	ppa_ovariect<br><br>Afficher le champ UNIQUEM ENT si :<br>[ppa_etapegen] = '4' or [ppa_etapegen] = '5'	Have you undergone or are you considering preventive bilateral salpingo ovariectomy (removal of ovaries and tubes) to reduce your risk of ovarian cancer?	radio<br>1 Yes, I had this surgery<br>2 Yes, I am considering this surgery<br>3 No, I have not had this surgery and I am not considering it.<br>4 Not applicable<br><br>Alignement personnalisé : LV
	58	ppa_ovariectan<br><br>Afficher le champ UNIQUEM ENT si :<br>[ppa_ovariect] = '1'	In what year did you have a preventive bilateral salpingo ovariectomy?	text (number, Min. : 1950, Max. : 2021)<br>Alignement personnalisé : LV

	59	ppa_info	During your meetings with the health care team, the information provided allowed you to fully understand:	checkbox<br>1 ppa_info___1 Your condition (stakes, diagnosis, evolution)<br>2 ppa_info___2 Your examinations (tests and investigations)<br>3 ppa_info___3 Your options, benefits and side effects<br>4 ppa_info___4 Your follow-up and care<br>5 ppa_info___5 None of the above<br>6 ppa_info___6 Other<br>Alignement personnalisé : LV
	60	ppa_infoautre<br>Afficher le champ UNIQUEMENT si :<br>[ppa_info(6)] = '1'	If you answered "Other", please specify :	text<br>Alignement personnalisé : LV
	61	pas_care_experience_questionnaire_complete	En-tête de section : *Form Status*<br>Complete?	dropdown<br>0 Incomplete<br>1 Unverified<br>2 Complete
Formulaire : **Previous personal experience with PAs questionnaire** (previous_personal_experience_with_pas_questionnair) Collapse				
	62	pa_jumelage_v2	En-tête de section : *CONTACT WITH A PATIENT ADVISOR DURING YOUR OWN CARE PATHWAY*<br>Have you ever been in contact with a patient advisor yourself?	radio<br>0 Yes<br>1 No and I would have liked to benefit from it<br>2 No, and I don't think I needed it.<br>Alignement personnalisé : LV
	63	pa_bidon2_v2_v2_v2<br>Afficher le champ UNIQUEMENT si :<br>[pa_jumelage_v2] = '0'	What topics did you discuss with the patient advisor? (Check all that apply)	descriptive
	64	pa_themeorg_v2<br>Afficher le champ UNIQUEMENT si :<br>[pa_jumelage_v2] = '0'	Organizational aspects	checkbox<br>0 pa_themeorg_v2___0 Her/His role as patient advisor<br>1 pa_themeorg_v2___1 The role of different health care professionals<br>2 pa_themeorg_v2___2 The role of external and internal organizations<br>3 pa_themeorg_v2___3 The care trajectory<br>4 pa_themeorg_v2___4 Rights as a patient (e.g., refusing treatment, asking questions)<br>5 pa_themeorg_v2___5 Where and how to get to medical appointments<br>6 pa_themeorg_v2___6 Financial support for patients and transportation assistance<br>7 pa_themeorg_v2___7 The Parole-Onco Research Project<br>8 pa_themeorg_v2___8 Other information<br>Alignement personnalisé : LV
	65	pa_themeorgautre_v2<br>Afficher le champ UNIQUEMENT si :<br>[pa_themeorg_v2(8)] = '1'	If other information, please specify which one:	text<br>Alignement personnalisé : LV

	66	pa_themecli_v2<br><br>Afficher le champ UNIQUEMENT si :<br>[pa_jumelage_v2] = '0'	Clinical aspects	checkbox<br>0 · pa_themecli_v2___0 · The announcement of the cancer diagnosis or genetic predisposition by the doctor<br>1 · pa_themecli_v2___1 · Cancer<br>2 · pa_themecli_v2___2 · Genetic testing<br>3 · pa_themecli_v2___3 · Therapeutic options in the case of cancer<br>4 · pa_themecli_v2___4 · Ways to reduce risk in carriers of a gene mutation that increases the risk of cancer<br>5 · pa_themecli_v2___5 · Surgical and reconstructive options for cancer<br>6 · pa_themecli_v2___6 · Breast implants<br>7 · pa_themecli_v2___7 · Hormonal issues<br>8 · pa_themecli_v2___8 · Reproductive issues<br>9 · pa_themecli_v2___9 · Urinary and erectile dysfunction issues<br>10 · pa_themecli_v2___10 · Fatigue following treatments<br>11 · pa_themecli_v2___11 · Pain and discomfort after treatment<br>12 · pa_themecli_v2___12 · Pain and discomfort after surgery<br>13 · pa_themecli_v2___13 · Possible emotions<br>14 · pa_themecli_v2___14 · Stress and anxiety management<br>15 · pa_themecli_v2___15 · Impact on physical appearance and self-esteem<br>16 · pa_themecli_v2___16 · Decision-making processes<br>17 · pa_themecli_v2___17 · Other information<br><br>Alignement personnalisé : LV
	67	pa_themecliautre_v2<br><br>Afficher le champ UNIQUEMENT si :<br>[pa_themecli_v2(17)] = '1'	If other information, please specify which one:	text<br>Alignement personnalisé : LV

	68	pa_themequo_v2<br><br>Afficher le champ UNIQUEMENT si :<br>[pa_jumelage_v2] = '0'	Consequences on daily life	checkbox<br>0 pa_themequo_v2___0 Consequences on children<br>1 pa_themequo_v2___1 Consequences on conjugal life<br>2 pa_themequo_v2___2 Consequences on sexual life<br>3 pa_themequo_v2___3 Returning to work<br>4 pa_themequo_v2___4 The return to daily life<br>5 pa_themequo_v2___5 The consequences on the spiritual life<br>6 pa_themequo_v2___6 How to announce a diagnosis to your loved ones<br>7 pa_themequo_v2___7 Financial implications<br>8 pa_themequo_v2___8 Consequences on insurance<br>9 pa_themequo_v2___9 Consequences on professional life<br>10 pa_themequo_v2___10 Social perception (the reaction of others)<br>11 pa_themequo_v2___11 Strategies for living the treatments in the best conditions<br>12 pa_themequo_v2___12 How to regain control over the disease<br>13 pa_themequo_v2___13 Other information<br><br>Alignement personnalisé : LV
	69	pa_themequoautre_v2<br><br>Afficher le champ UNIQUEMENT si :<br>[pa_themequo_v2(13)] = '1'	If other information, please specify which one:	text<br>Alignement personnalisé : LV
	70	pa_bene_v2<br><br>Afficher le champ UNIQUEMENT si :<br>[pa_jumelage_v2] = '0'	In what area(s) has the accompaniment been beneficial? (You may check more than one answer.)	checkbox<br>0 pa_bene_v2___0 My understanding of my care pathway within the health care institution (steps, role of each person...)<br>1 pa_bene_v2___1 My relationships and exchanges with the medical and nursing teams<br>2 pa_bene_v2___2 My experience of the disease (sharing experiences on side effects, on the impact of the disease on daily life, with those around me...)<br>3 pa_bene_v2___3 My quality of life (decrease in stress level, anxiety, feeling understood...)<br>4 pa_bene_v2___4 My knowledge of services and associations that can help me.<br>5 pa_bene_v2___5 This accompaniment has not been particularly beneficial to me.<br>6 pa_bene_v2___6 Other<br><br>Alignement personnalisé : LV
	71	pa_bene_v2_autre<br><br>Afficher le champ UNIQUEMENT si :<br>[pa_bene_v2(6)] = '1'	If other benefit, please specify which one:	text<br>Alignement personnalisé : LV

<table>
<tr><td></td><td>72</td><td>pa_contri_v2

Afficher le champ UNIQUEM ENT si :
[pa_jumelage_v2] = '0'</td><td>In your opinion, what was the contribution of the patient advisor? You may check more than one answer.</td><td>checkbox
<table>
<tr><td>0</td><td>pa_contri_v2___0</td><td>Giving information</td></tr>
<tr><td>1</td><td>pa_contri_v2___1</td><td>Giving support</td></tr>
<tr><td>2</td><td>pa_contri_v2___2</td><td>Sharing experiences</td></tr>
<tr><td>3</td><td>pa_contri_v2___3</td><td>Facilitate communication between the health care team and the patient advisor.</td></tr>
<tr><td>4</td><td>pa_contri_v2___4</td><td>Helping to bring the patient's perspective to the health care team</td></tr>
<tr><td>5</td><td>pa_contri_v2___5</td><td>Improve understanding of information shared between the patient and the health care team</td></tr>
<tr><td>6</td><td>pa_contri_v2___6</td><td>Helping to remember information</td></tr>
<tr><td>7</td><td>pa_contri_v2___7</td><td>Preparing the patient for medical appointments</td></tr>
<tr><td>8</td><td>pa_contri_v2___8</td><td>Accompanying patients to medical appointments</td></tr>
<tr><td>9</td><td>pa_contri_v2___9</td><td>Helping the patient participate in decision making</td></tr>
<tr><td>10</td><td>pa_contri_v2___10</td><td>Bringing a new perspective to decision making</td></tr>
<tr><td>11</td><td>pa_contri_v2___11</td><td>Referring patients to a variety of resources</td></tr>
<tr><td>12</td><td>pa_contri_v2___12</td><td>Help develop a trusting relationship between the patient and the health care team</td></tr>
<tr><td>13</td><td>pa_contri_v2___13</td><td>Helping the patient become a partner in his or her own care</td></tr>
<tr><td>14</td><td>pa_contri_v2___14</td><td>Listening to the patient</td></tr>
<tr><td>15</td><td>pa_contri_v2___15</td><td>Other contributions</td></tr>
<tr><td>16</td><td>pa_contri_v2___16</td><td>No, the patient advisor has no particular contribution to make to the care trajectory.</td></tr>
</table>
Alignement personnalisé : LV</td></tr>
<tr><td></td><td>73</td><td>pa_contriautre_v2

Afficher le champ UNIQUEM ENT si :
[pa_contri_v2(15)] = '1'</td><td>Please indicate what other contributions the patient advisor has had.</td><td>text
Alignement personnalisé : LV</td></tr>
<tr><td></td><td>74</td><td>pa_bidon3_v2_v2_v2

Afficher le champ UNIQUEM ENT si :
[pa_jumelage_v2] = '1'</td><td>What topics would you have liked to discuss with a patient advisor? You can check more than one answer.</td><td>descriptive</td></tr>
</table>

<table>
<tr><td></td><td>75</td><td>pa_orgpo_v2

Afficher le champ UNIQUEMENT si :
[pa_jumelage_v2] = '1'</td><td>Organizational aspects</td><td>checkbox
<table>
<tr><td>0</td><td>pa_orgpo_v2___0</td><td>Her/His role as patient advisor</td></tr>
<tr><td>1</td><td>pa_orgpo_v2___1</td><td>The role of different health care professionals</td></tr>
<tr><td>2</td><td>pa_orgpo_v2___2</td><td>The role of external and internal organizations</td></tr>
<tr><td>3</td><td>pa_orgpo_v2___3</td><td>The care trajectory</td></tr>
<tr><td>4</td><td>pa_orgpo_v2___4</td><td>Rights as a patient (e.g., refusing treatment, asking questions)</td></tr>
<tr><td>5</td><td>pa_orgpo_v2___5</td><td>Where and how to get to medical appointments</td></tr>
<tr><td>6</td><td>pa_orgpo_v2___6</td><td>Financial support for patients and transportation assistance</td></tr>
<tr><td>7</td><td>pa_orgpo_v2___7</td><td>The Parole-Onco Research Project</td></tr>
<tr><td>8</td><td>pa_orgpo_v2___8</td><td>Other information</td></tr>
</table>
Alignement personnalisé : LV</td></tr>
<tr><td></td><td>76</td><td>pa_orgpoautre_v2

Afficher le champ UNIQUEMENT si :
[pa_orgpo_v2(8)] = '1'</td><td>If other information, please specify which one:</td><td>text
Alignement personnalisé : LV</td></tr>
</table>

<table>
<tr>
<td></td>
<td>77</td>
<td>pa_ppaclipo_v2_v2_v2

Afficher le champ UNIQUEM ENT si :
[pa_jumelage_v2] = '1'</td>
<td>Clinical aspects</td>
<td>checkbox
<table>
<tr><td>0</td><td>pa_ppaclipo_v2_v2_v2___0</td><td>Announcement of cancer diagnosis/genetic predisposition by the doctor</td></tr>
<tr><td>1</td><td>pa_ppaclipo_v2_v2_v2___1</td><td>Cancer</td></tr>
<tr><td>2</td><td>pa_ppaclipo_v2_v2_v2___2</td><td>Genetic testing</td></tr>
<tr><td>3</td><td>pa_ppaclipo_v2_v2_v2___3</td><td>Therapeutic options in the case of cancer</td></tr>
<tr><td>4</td><td>pa_ppaclipo_v2_v2_v2___4</td><td>Ways to reduce risk in carriers of a gene mutation that increases the risk of cancer</td></tr>
<tr><td>5</td><td>pa_ppaclipo_v2_v2_v2___5</td><td>Surgical and re-constructive options in the case of cancer</td></tr>
<tr><td>6</td><td>pa_ppaclipo_v2_v2_v2___6</td><td>Breast implants</td></tr>
<tr><td>7</td><td>pa_ppaclipo_v2_v2_v2___7</td><td>Hormonal issues</td></tr>
<tr><td>8</td><td>pa_ppaclipo_v2_v2_v2___8</td><td>Reproductive issues</td></tr>
<tr><td>9</td><td>pa_ppaclipo_v2_v2_v2___9</td><td>The issues of urinary and erectile dysfunctions</td></tr>
<tr><td>10</td><td>pa_ppaclipo_v2_v2_v2___10</td><td>Fatigue following treatments</td></tr>
<tr><td>11</td><td>pa_ppaclipo_v2_v2_v2___11</td><td>Pain and discomfort after treatment</td></tr>
<tr><td>12</td><td>pa_ppaclipo_v2_v2_v2___12</td><td>Pain and discomfort after surgery</td></tr>
<tr><td>13</td><td>pa_ppaclipo_v2_v2_v2___13</td><td>Possible emotions</td></tr>
<tr><td>14</td><td>pa_ppaclipo_v2_v2_v2___14</td><td>Stress and anxiety management</td></tr>
<tr><td>15</td><td>pa_ppaclipo_v2_v2_v2___15</td><td>Impact on physical appearance and self-esteem</td></tr>
<tr><td>16</td><td>pa_ppaclipo_v2_v2_v2___16</td><td>Decision-making processes</td></tr>
<tr><td>17</td><td>pa_ppaclipo_v2_v2_v2___17</td><td>Other information</td></tr>
</table>
Alignement personnalisé : LV</td>
</tr>
<tr>
<td></td>
<td>78</td>
<td>pa_ppaclipoautre_v2_v2_v2

Afficher le champ UNIQUEM ENT si :
[pa_ppaclipo_v2_v2_v2(17)] = '1'</td>
<td>If other information, please specify which one:</td>
<td>text
Alignement personnalisé : LV</td>
</tr>
</table>

	79	pa_quopo_v2<br><br>Afficher le champ UNIQUEM ENT si :<br>[pa_jumelage_v2] = '1'	Consequences on daily life	checkbox<br>1 | pa_quopo_v2___1 | Consequences on children<br>2 | pa_quopo_v2___2 | Consequences on conjugal life<br>3 | pa_quopo_v2___3 | Consequences on sexual life<br>4 | pa_quopo_v2___4 | Returning to work<br>5 | pa_quopo_v2___5 | The return to daily life<br>6 | pa_quopo_v2___6 | The consequences on the spiritual life<br>7 | pa_quopo_v2___7 | How to announce a diagnosis to your loved ones<br>8 | pa_quopo_v2___8 | Financial implications<br>9 | pa_quopo_v2___9 | Consequences on insurance<br>10 | pa_quopo_v2___10 | Consequences on professional life<br>11 | pa_quopo_v2___11 | Social perception (the reaction of others)<br>12 | pa_quopo_v2___12 | Strategies for living the treatments in the best conditions<br>13 | pa_quopo_v2___13 | How to regain control over the disease<br>14 | pa_quopo_v2___14 | Other information<br><br>Alignement personnalisé : LV
	80	pa_ppaquopoautre_v2_v2_v2<br><br>Afficher le champ UNIQUEM ENT si :<br>[pa_quopo_v2(14)] = '1'	If other information, please specify which one:	text<br>Alignement personnalisé : LV

	81	pa_contripo_v2<br>Afficher le champ UNIQUEM ENT si :<br>[pa_jumelage_v2] = '1' or [pa_jumelage_v2] = '2'	In your opinion, what could be/could have been the contribution of the patient advisor?	checkbox<br>0 | pa_contripo_v2___0 | Giving information<br>1 | pa_contripo_v2___1 | Giving support<br>2 | pa_contripo_v2___2 | Sharing experiences<br>3 | pa_contripo_v2___3 | Facilitate communication between the health care team and the patient advisor.<br>4 | pa_contripo_v2___4 | Helping to bring the patient's perspective to the health care team<br>5 | pa_contripo_v2___5 | Improve understanding of information shared between the patient and the health care team<br>6 | pa_contripo_v2___6 | Helping to remember information<br>7 | pa_contripo_v2___7 | Preparing the patient for medical appointments<br>8 | pa_contripo_v2___8 | Accompanying patients to medical appointments<br>9 | pa_contripo_v2___9 | Helping the patient participate in decision making<br>10 | pa_contripo_v2___10 | Bringing a new perspective to decision making<br>11 | pa_contripo_v2___11 | Referring patients to a variety of resources<br>12 | pa_contripo_v2___12 | Help develop a trusting relationship between the patient and the health care team<br>13 | pa_contripo_v2___13 | Helping the patient become a partner in his or her own care<br>14 | pa_contripo_v2___14 | Listening to the patient<br>15 | pa_contripo_v2___15 | Other contribution<br>16 | pa_contripo_v2___16 | No, the patient advisor has no particular contribution to make to the care trajectory.<br>Alignement personnalisé : LV
	82	pa_ppacontripoautre_v2<br>Afficher le champ UNIQUEM ENT si :<br>[pa_contripo_v2(15)] = '1'	Please indicate what other contribution could make/could have been made by the patient advisor.	text<br>Alignement personnalisé : LV
	83	previous_personal_experience_with_pas_questionnair_complete	En-tête de section : *Form Status*<br>Complete?	dropdown<br>0 | Incomplete<br>1 | Unverified<br>2 | Complete

Formulaire : **PA-K6** (pak6) Collapse

	84	pa_k6nerv_v2_v2_v2	During the past 30 days, about how often did you feel nervous?	radio (Matrice)<br>1 | All of the time<br>2 | Most of the time<br>3 | Some of the time<br>4 | A little of the time<br>5 | None of the time

	85	pa_k6deses_v2_v2_v2	During the past 30 days, about how often did you feel hopeless?	radio (Matrice)<br>1 All of the time<br>2 Most of the time<br>3 Some of the time<br>4 A little of the time<br>5 None of the time
	86	pa_k6agite_v2_v2_v2	During the past 30 days, about how often did you feel restless or fidgety?	radio (Matrice)<br>1 All of the time<br>2 Most of the time<br>3 Some of the time<br>4 A little of the time<br>5 None of the time
	87	pa_k6sourire_v2_v2_v2	During the past 30 days, about how often did you feel so depressed that nothing could cheer you up?	radio (Matrice)<br>1 All of the time<br>2 Most of the time<br>3 Some of the time<br>4 A little of the time<br>5 None of the time
	88	pa_k6effort_v2_v2_v2	During the past 30 days, about how often did you feel that everything was an effort?	radio (Matrice)<br>1 All of the time<br>2 Most of the time<br>3 Some of the time<br>4 A little of the time<br>5 None of the time
	89	pa_k6rien_v2_v2_v2	During the past 30 days, about how often did you feel worthless?	radio (Matrice)<br>1 All of the time<br>2 Most of the time<br>3 Some of the time<br>4 A little of the time<br>5 None of the time
	90	pak6_complete	En-tête de section : *Form Status*<br>Complete?	dropdown<br>0 Incomplete<br>1 Unverified<br>2 Complete
Formulaire : **Review of PA experience with patient questionnaire** (review_of_pa_experience_with_patient_questionnaire) Collapse				
	91	bilan_eval_res_form	En-tête de section : *The first section of this questionnaire aims to assess your experience as a patient advisor. The questions asked are designed to assess the extent to which the intervention implemented helped you to play your role as a patient advisor. To what extent do you agree with each of the following statements?*<br>The training you have received is sufficient.	radio (Matrice)<br>1 Strongly Disagree<br>2 Somewhat Disagree<br>3 Somewhat agree<br>4 Strongly agree
	92	bilan_eval_res_out	You felt sufficiently equipped to meet the needs of patients.	radio (Matrice)<br>1 Strongly Disagree<br>2 Somewhat Disagree<br>3 Somewhat agree<br>4 Strongly agree
	93	bilan_eval_res_conf	You could attend conferences and continuing education activities.	radio (Matrice)<br>1 Strongly Disagree<br>2 Somewhat Disagree<br>3 Somewhat agree<br>4 Strongly agree

	94	bilan_eval_res_ech	Discussions and exchanges with the other patient advisors were sufficient.	radio (Matrice)<br>1 Strongly Disagree<br>2 Somewhat Disagree<br>3 Somewhat agree<br>4 Strongly agree
	95	bilan_eval_res_logis	You felt supported in the logistical organization of patient meetings.	radio (Matrice)<br>1 Strongly Disagree<br>2 Somewhat Disagree<br>3 Somewhat agree<br>4 Strongly agree
	96	bilan_eval_res_role	You have always felt comfortable with the roles and responsibilities assigned to you.	radio (Matrice)<br>1 Strongly Disagree<br>2 Somewhat Disagree<br>3 Somewhat agree<br>4 Strongly agree
	97	bilan_eval_res_infequ	Means have been put in place to inform the health care team of your interventions.	radio (Matrice)<br>1 Strongly Disagree<br>2 Somewhat Disagree<br>3 Somewhat agree<br>4 Strongly agree
	98	bilan_eval_res_compro	It was easy to communicate with the health professionals involved.	radio (Matrice)<br>1 Strongly Disagree<br>2 Somewhat Disagree<br>3 Somewhat agree<br>4 Strongly agree
	99	bilan_eval_res_reu	You could attend health care team meetings.	radio (Matrice)<br>1 Strongly Disagree<br>2 Somewhat Disagree<br>3 Somewhat agree<br>4 Strongly agree
	100	bilan_eval_res_integ	You felt part of the care team.	radio (Matrice)<br>1 Strongly Disagree<br>2 Somewhat Disagree<br>3 Somewhat agree<br>4 Strongly agree
	101	bilan_eval_res_expe	The people you accompanied had experiences similar to yours.	radio (Matrice)<br>1 Strongly Disagree<br>2 Somewhat Disagree<br>3 Somewhat agree<br>4 Strongly agree
	102	bilan_eval_res_adeq	The number of accompaniments you provided was adequate.	radio (Matrice)<br>1 Strongly Disagree<br>2 Somewhat Disagree<br>3 Somewhat agree<br>4 Strongly agree
	103	bilan_eval_res_avant	You had certain advantages in the establishment (parking, preferential rates).	radio (Matrice)<br>1 Strongly Disagree<br>2 Somewhat Disagree<br>3 Somewhat agree<br>4 Strongly agree

	104	bilan_sout_eq_logis	En-tête de section : *At least one member of the oncology care team (nurses, physicians, social workers, psychologists, etc.)*<br>...supported you in the logistical organization (room, documentation...) of patient meetings.	radio (Matrice)<br>1 Strongly Disagree<br>2 Somewhat Disagree<br>3 Somewhat agree<br>4 Strongly agree
	105	bilan_sout_eq_superv	...supervised your interventions with patients.	radio (Matrice)<br>1 Strongly Disagree<br>2 Somewhat Disagree<br>3 Somewhat agree<br>4 Strongly agree
	106	bilan_sout_eq_quest	...took the necessary time to answer your questions and discuss your interventions with you.	radio (Matrice)<br>1 Strongly Disagree<br>2 Somewhat Disagree<br>3 Somewhat agree<br>4 Strongly agree
	107	bilan_sout_eq_patacc	...was available to provide feedback to the people who benefited from your interventions.	radio (Matrice)<br>1 Strongly Disagree<br>2 Somewhat Disagree<br>3 Somewhat agree<br>4 Strongly agree
	108	chumpa_v2<br>Afficher le champ UNIQUEMENT si :<br>[0_arm_1][ppa_recru] = '0'	At least one member of the Virage team (nurses, doctors, social workers, psychologists, etc.)	descriptive
	109	bilan_virage_logis<br>Afficher le champ UNIQUEMENT si :<br>[0_arm_1][ppa_recru] = '0'	...supported you in the logistical organization (room, documentation...) of patient meetings.	radio (Matrice)<br>1 Strongly Disagree<br>2 Somewhat Disagree<br>3 Somewhat agree<br>4 Strongly agree
	110	bilan_virage_superv<br>Afficher le champ UNIQUEMENT si :<br>[0_arm_1][ppa_recru] = '0'	...supervised your interventions with patients.	radio (Matrice)<br>1 Strongly Disagree<br>2 Somewhat Disagree<br>3 Somewhat agree<br>4 Strongly agree
	111	bilan_virage_quest<br>Afficher le champ UNIQUEMENT si :<br>[0_arm_1][ppa_recru] = '0'	...took the necessary time to answer your questions and discuss your interventions with you.	radio (Matrice)<br>1 Strongly Disagree<br>2 Somewhat Disagree<br>3 Somewhat agree<br>4 Strongly agree
	112	bilan_virage_patacc<br>Afficher le champ UNIQUEMENT si :<br>[0_arm_1][ppa_recru] = '0'	...was available to provide feedback to the people who benefited from your interventions.	radio (Matrice)<br>1 Strongly Disagree<br>2 Somewhat Disagree<br>3 Somewhat agree<br>4 Strongly agree
	113	cemtlpa_v2<br>Afficher le champ UNIQUEMENT si :<br>[0_arm_1][ppa_recru] = '2'	At least one member of the CROIRE team (nurses, doctors, social workers, psychologists, etc.)	descriptive
	114	bilan_croire_logis<br>Afficher le champ UNIQUEMENT si :<br>[0_arm_1][ppa_recru] = '2'	...supported you in the logistical organization (room, documentation...) of patient meetings.	radio (Matrice)<br>1 Strongly Disagree<br>2 Somewhat Disagree<br>3 Somewhat agree<br>4 Strongly agree

	115	bilan_croire_superv<br>Afficher le champ UNIQUEMENT si :<br>[0_arm_1][ppa_recru] = '2'	...supervised your interventions with patients.	radio (Matrice)<br>1 Strongly Disagree<br>2 Somewhat Disagree<br>3 Somewhat agree<br>4 Strongly agree
	116	bilan_croire_quest<br>Afficher le champ UNIQUEMENT si :<br>[0_arm_1][ppa_recru] = '2'	...took the necessary time to answer your questions and discuss your interventions with you.	radio (Matrice)<br>1 Strongly Disagree<br>2 Somewhat Disagree<br>3 Somewhat agree<br>4 Strongly agree
	117	bilan_croire_patacc<br>Afficher le champ UNIQUEMENT si :<br>[0_arm_1][ppa_recru] = '2'	...was available to provide feedback to the people who benefited from your interventions.	radio (Matrice)<br>1 Strongly Disagree<br>2 Somewhat Disagree<br>3 Somewhat agree<br>4 Strongly agree
	118	chupa_v2<br>Afficher le champ UNIQUEMENT si :<br>[0_arm_1][ppa_recru] = '3'	At least one member of the Quebec Cancer Foundation team (nurses, doctors, social workers, psychologists, etc.)	descriptive
	119	bilan_fqc_logis<br>Afficher le champ UNIQUEMENT si :<br>[0_arm_1][ppa_recru] = '3'	...supported you in the logistical organization (room, documentation...) of patient meetings.	radio (Matrice)<br>1 Strongly Disagree<br>2 Somewhat Disagree<br>3 Somewhat agree<br>4 Strongly agree
	120	bilan_fqc_superv<br>Afficher le champ UNIQUEMENT si :<br>[0_arm_1][ppa_recru] = '3'	...supervised your interventions with patients.	radio (Matrice)<br>1 Strongly Disagree<br>2 Somewhat Disagree<br>3 Somewhat agree<br>4 Strongly agree
	121	bilan_fqc_quest<br>Afficher le champ UNIQUEMENT si :<br>[0_arm_1][ppa_recru] = '3'	...took the necessary time to answer your questions and discuss your interventions with you.	radio (Matrice)<br>1 Strongly Disagree<br>2 Somewhat Disagree<br>3 Somewhat agree<br>4 Strongly agree
	122	bilan_fqc_patacc<br>Afficher le champ UNIQUEMENT si :<br>[0_arm_1][ppa_recru] = '3'	...was available to provide feedback to the people who benefited from your interventions.	radio (Matrice)<br>1 Strongly Disagree<br>2 Somewhat Disagree<br>3 Somewhat agree<br>4 Strongly agree
	123	gaspepa_v2<br>Afficher le champ UNIQUEMENT si :<br>[0_arm_1][ppa_recru] = '6'	At least one member of the PROCURE team (nurses, doctors, social workers, psychologists, etc.)	descriptive
	124	bilan_proc_logis<br>Afficher le champ UNIQUEMENT si :<br>[0_arm_1][ppa_recru] = '6'	...supported you in the logistical organization (room, documentation...) of patient meetings.	radio (Matrice)<br>1 Strongly Disagree<br>2 Somewhat Disagree<br>3 Somewhat agree<br>4 Strongly agree
	125	bilan_proc_superv<br>Afficher le champ UNIQUEMENT si :<br>[0_arm_1][ppa_recru] = '6'	...supervised your interventions with patients.	radio (Matrice)<br>1 Strongly Disagree<br>2 Somewhat Disagree<br>3 Somewhat agree<br>4 Strongly agree

	126	bilan_proc_quest<br>Afficher le champ UNIQUEM ENT si :<br>[0_arm_1][ppa_recru] = '6'	...took the necessary time to answer your questions and discuss your interventions with you.	radio (Matrice)<br>1 Strongly Disagree<br>2 Somewhat Disagree<br>3 Somewhat agree<br>4 Strongly agree
	127	bilan_proc_patacc<br>Afficher le champ UNIQUEM ENT si :<br>[0_arm_1][ppa_recru] = '6'	...was available to provide feedback to the people who benefited from your interventions.	radio (Matrice)<br>1 Strongly Disagree<br>2 Somewhat Disagree<br>3 Somewhat agree<br>4 Strongly agree
	128	gaspepa_ogpac_v2<br>Afficher le champ UNIQUEM ENT si :<br>[0_arm_1][ppa_recru] = '6'	At least one member of the OGPAC team (nurses, doctors, social workers, psychologists, etc.)	descriptive
	129	bilan_ogpac_logis<br>Afficher le champ UNIQUEM ENT si :<br>[0_arm_1][ppa_recru] = '6'	...supported you in the logistical organization (room, documentation...) of patient meetings.	radio (Matrice)<br>1 Strongly Disagree<br>2 Somewhat Disagree<br>3 Somewhat agree<br>4 Strongly agree
	130	bilan_ogpac_superv<br>Afficher le champ UNIQUEM ENT si :<br>[0_arm_1][ppa_recru] = '6'	...supervised your interventions with patients.	radio (Matrice)<br>1 Strongly Disagree<br>2 Somewhat Disagree<br>3 Somewhat agree<br>4 Strongly agree
	131	bilan_ogpac_quest<br>Afficher le champ UNIQUEM ENT si :<br>[0_arm_1][ppa_recru] = '6'	...took the necessary time to answer your questions and discuss your interventions with you.	radio (Matrice)<br>1 Strongly Disagree<br>2 Somewhat Disagree<br>3 Somewhat agree<br>4 Strongly agree
	132	bilan_ogpac_patacc<br>Afficher le champ UNIQUEM ENT si :<br>[0_arm_1][ppa_recru] = '6'	...was available to provide feedback to the people who benefited from your interventions.	radio (Matrice)<br>1 Strongly Disagree<br>2 Somewhat Disagree<br>3 Somewhat agree<br>4 Strongly agree
	133	ctreouestpa_v2<br>Afficher le champ UNIQUEM ENT si :<br>[0_arm_1][ppa_recru] = '5'	At least one member of the Hope and Cope team (nurses, doctors, social workers, psychologists, etc.)	descriptive
	134	bilan_co_logis<br>Afficher le champ UNIQUEM ENT si :<br>[0_arm_1][ppa_recru] = '5'	...supported you in the logistical organization (room, documentation...) of patient meetings.	radio (Matrice)<br>1 Strongly Disagree<br>2 Somewhat Disagree<br>3 Somewhat agree<br>4 Strongly agree
	135	bilan_co_interv<br>Afficher le champ UNIQUEM ENT si :<br>[0_arm_1][ppa_recru] = '5'	...supervised your interventions with patients.	radio (Matrice)<br>1 Strongly Disagree<br>2 Somewhat Disagree<br>3 Somewhat agree<br>4 Strongly agree
	136	bilan_co_quest<br>Afficher le champ UNIQUEM ENT si :<br>[0_arm_1][ppa_recru] = '5'	...took the necessary time to answer your questions and discuss your interventions with you.	radio (Matrice)<br>1 Strongly Disagree<br>2 Somewhat Disagree<br>3 Somewhat agree<br>4 Strongly agree

	137	bilan_co_patacc<br><br>Afficher le champ UNIQUEMENT si :<br>[0_arm_1][ppa_recru] = '5'	...was available to provide feedback to the people who benefited from your interventions.	radio (Matrice)<br>1 Strongly Disagree<br>2 Somewhat Disagree<br>3 Somewhat agree<br>4 Strongly agree
	138	paq39_v2	The logbook at your disposal to keep track of your interventions has been useful to you.	radio<br>1 Strongly disagree<br>2 Somewhat disagree<br>3 Somewhat agree<br>4 Strongly agree<br><br>Alignement personnalisé : LV
	139	paq40_v2	Are there other tools at yout disposal?	radio<br>1 Yes<br>2 No<br><br>Alignement personnalisé : LV
	140	paq41_v2<br><br>Afficher le champ UNIQUEMENT si :<br>[paq40_v2] = '1'	Other tool at your disposal #1:	text<br>Alignement personnalisé : LH
	141	paq41a_v2<br><br>Afficher le champ UNIQUEMENT si :<br>[paq40_v2] = '1'	This tool put at your disposal has been useful to you.	radio<br>1 Strongly Disagree<br>2 Somewhat Disagree<br>3 Somewhat agree<br>4 Strongly agree<br><br>Alignement personnalisé : LV
	142	paq40_v3<br><br>Afficher le champ UNIQUEMENT si :<br>[paq40_v2] <> "" and [paq40_v2] = '1'	Are there other tools at your disposal?	radio<br>1 Yes<br>2 No<br><br>Alignement personnalisé : LV
	143	paq41_2_v2<br><br>Afficher le champ UNIQUEMENT si :<br>[paq40_v3] = '1'	Other tool at your disposal #2:	text<br>Alignement personnalisé : LH
	144	paq41b_v2<br><br>Afficher le champ UNIQUEMENT si :<br>[paq40_v3] = '1'	This tool put at your disposal has been useful to you .	radio<br>1 Strongly Disagree<br>2 Somewhat Disagree<br>3 Somewhat agree<br>4 Strongly agree<br><br>Alignement personnalisé : LV
	145	paq40_v4<br><br>Afficher le champ UNIQUEMENT si :<br>[paq40_v3] <> "" and [paq40_v3] = '1'	Are there other tools at your disposal?	radio<br>1 Yes<br>2 No<br><br>Alignement personnalisé : LV
	146	paq41_3_v2<br><br>Afficher le champ UNIQUEMENT si :<br>[paq40_v4] = '1'	Other tool at your disposal #3:	text<br>Alignement personnalisé : LH

	147	paq41c_v2<br>Afficher le champ UNIQUEM ENT si :<br>[paq40_v4] = '1'	This tool put at your disposal has been useful to you.	radio<br>1 Strongly Disagree<br>2 Somewhat Disagree<br>3 Somewhat agree<br>4 Strongly agree<br>Alignement personnalisé : LV
	148	paq40_v5<br>Afficher le champ UNIQUEM ENT si :<br>[paq40_v4] <> "" and [paq40_ v4] = '1'	Are there other tools at your disposal?	radio<br>1 Yes<br>2 No<br>Alignement personnalisé : LV
	149	paq41_4_v2<br>Afficher le champ UNIQUEM ENT si :<br>[paq40_v5] = '1'	Other tool at your disposal #4:	text<br>Alignement personnalisé : LH
	150	paq41d_v2<br>Afficher le champ UNIQUEM ENT si :<br>[paq40_v5] = '1'	This tool put at your disposal has been useful to you.	radio<br>1 Strongly Disagree<br>2 Somewhat Disagree<br>3 Somewhat agree<br>4 Strongly agree<br>Alignement personnalisé : LV
	151	paq42_v2	Please indicate your overall level of appreciation of your experience as a patient advisor using the response options below.	radio<br>1 I did not enjoy my experience<br>2 I moderately appreciated my experience<br>3 I enjoyed my experience<br>4 I enjoyed my experience very much<br>5 Not applicable<br>Alignement personnalisé : LV
	152	paq43a_v2	En-tête de section : *The purpose of this section is to identify the information that has been provided to you to assist you in your role as a patient advisor.*<br>Organizational aspects<br>Did you receive the information you needed regarding...	descriptive
	153	bilan_org_role	...your role as a patient advisor?	radio (Matrice)<br>1 No<br>2 Yes, in part<br>3 Yes, absolutely<br>4 I didn't need it<br>5 Not applicable
	154	bilan_org_rolpro	...the role of different health professionals?	radio (Matrice)<br>1 No<br>2 Yes, in part<br>3 Yes, absolutely<br>4 I didn't need it<br>5 Not applicable
	155	bilan_org_organisme	...the role of external and internal organizations?	radio (Matrice)<br>1 No<br>2 Yes, in part<br>3 Yes, absolutely<br>4 I didn't need it<br>5 Not applicable

	156	bilan_org_traj	...the care trajectory?	radio (Matrice)<br>1 No<br>2 Yes, in part<br>3 Yes, absolutely<br>4 I didn't need it<br>5 Not applicable
	157	bilan_org_droits	...the patient's rights (e.g., refusing treatment, asking questions)?	radio (Matrice)<br>1 No<br>2 Yes, in part<br>3 Yes, absolutely<br>4 I didn't need it<br>5 Not applicable
	158	bilan_org_rdvmed	...where and how to get to medical appointments?	radio (Matrice)<br>1 No<br>2 Yes, in part<br>3 Yes, absolutely<br>4 I didn't need it<br>5 Not applicable
	159	bilan_org_transp	...financial support for patients and transportation assistance?	radio (Matrice)<br>1 No<br>2 Yes, in part<br>3 Yes, absolutely<br>4 I didn't need it<br>5 Not applicable
	160	bilan_org_projet	...the PAROLE-Onco research project?	radio (Matrice)<br>1 No<br>2 Yes, in part<br>3 Yes, absolutely<br>4 I didn't need it<br>5 Not applicable
	161	bilan_org_autre	Did you receive any other organizational information?	radio<br>0 No<br>1 Yes<br><br>Alignement personnalisé : LH
	162	paq52_v2<br>Afficher le champ UNIQUEMENT si :<br>[bilan_org_autre] = '1'	If yes, please specify which ones.	notes<br>Alignement personnalisé : LH
	163	bilan_relat_savoirs	En-tête de section : *The relational aspects Did you receive the information you needed regarding...*<br>...the attitudes and the skills to have?	radio (Matrice)<br>1 No<br>2 Yes, in part<br>3 Yes, absolutely<br>4 I didn't need it<br>5 Not applicable
	164	bilan_relat_fami	...familiarity?	radio (Matrice)<br>1 No<br>2 Yes, in part<br>3 Yes, absolutely<br>4 I didn't need it<br>5 Not applicable

	165	bilan_relat_imprev	...managing unexpected situations?	radio (Matrice)<br>1 No<br>2 Yes, in part<br>3 Yes, absolutely<br>4 I didn't need it<br>5 Not applicable
	166	bilan_relat_conflit	...conflict management?	radio (Matrice)<br>1 No<br>2 Yes, in part<br>3 Yes, absolutely<br>4 I didn't need it<br>5 Not applicable
	167	bilan_relat_aide	...the helping relationship?	radio (Matrice)<br>1 No<br>2 Yes, in part<br>3 Yes, absolutely<br>4 I didn't need it<br>5 Not applicable
	168	bilan_org_autre_2	Did you receive any other information on relational aspects?	radio<br>1 Yes<br>0 No<br><br>Alignement personnalisé : LH
	169	paq59_v2<br>Afficher le champ UNIQUEMENT si :<br>[bilan_org_autre_2] = '1'	If yes, please specify which ones.	notes<br>Alignement personnalisé : LV
	170	bilan_clin_annon	En-tête de section : *Clinical aspects Did you receive the information you needed regarding...*<br>...the announcement of the cancer diagnosis/genetic predisposition by the doctor?	radio (Matrice)<br>1 No<br>2 Yes, in part<br>3 Yes, absolutely<br>4 I didn't need it<br>5 Not applicable
	171	bilan_clin_cancer	...cancer?	radio (Matrice)<br>1 No<br>2 Yes, in part<br>3 Yes, absolutely<br>4 I didn't need it<br>5 Not applicable
	172	bilan_clin_oncogen	... oncogenetics?	radio (Matrice)<br>1 No<br>2 Yes, in part<br>3 Yes, absolutely<br>4 I didn't need it<br>5 Not applicable
	173	bilan_clin_tests	...genetic testing?	radio (Matrice)<br>1 No<br>2 Yes, in part<br>3 Yes, absolutely<br>4 I didn't need it<br>5 Not applicable

	174	bilan_clin_optthera	...treatment options in the case of cancer?	radio (Matrice)<br>1 No<br>2 Yes, in part<br>3 Yes, absolutely<br>4 I didn't need it<br>5 Not applicable
	175	bilan_clin_reducrisq	...ways to reduce the risk in carriers of a gene mutation that increases the risk of cancer?	radio (Matrice)<br>1 No<br>2 Yes, in part<br>3 Yes, absolutely<br>4 I didn't need it<br>5 Not applicable
	176	bilan_clin_options	...surgical and reconstructive options in the case of cancer?	radio (Matrice)<br>1 No<br>2 Yes, in part<br>3 Yes, absolutely<br>4 I didn't need it<br>5 Not applicable
	177	bilan_clin_proth	...breast implants?	radio (Matrice)<br>1 No<br>2 Yes, in part<br>3 Yes, absolutely<br>4 I didn't need it<br>5 Not applicable
	178	bilan_clin_hormo	...hormonal issues?	radio (Matrice)<br>1 No<br>2 Yes, in part<br>3 Yes, absolutely<br>4 I didn't need it<br>5 Not applicable
	179	bilan_clin_repro	...reproductive issues?	radio (Matrice)<br>1 No<br>2 Yes, in part<br>3 Yes, absolutely<br>4 I didn't need it<br>5 Not applicable
	180	bilan_clin_fatig	...fatigue following treatments?	radio (Matrice)<br>1 No<br>2 Yes, in part<br>3 Yes, absolutely<br>4 I didn't need it<br>5 Not applicable
	181	bilan_clin_doulchir	...pain and discomfort after surgery?	radio (Matrice)<br>1 No<br>2 Yes, in part<br>3 Yes, absolutely<br>4 I didn't need it<br>5 Not applicable

	182	bilan_clin_doultrait	...pain and discomfort after treatment?	radio (Matrice)<br>1 No<br>2 Yes, in part<br>3 Yes, absolutely<br>4 I didn't need it<br>5 Not applicable
	183	bilan_clin_emot	...the possible emotions?	radio (Matrice)<br>1 No<br>2 Yes, in part<br>3 Yes, absolutely<br>4 I didn't need it<br>5 Not applicable
	184	bilan_clin_stress	...stress and anxiety management?	radio (Matrice)<br>1 No<br>2 Yes, in part<br>3 Yes, absolutely<br>4 I didn't need it<br>5 Not applicable
	185	bilan_clin_appar	...the impact on physical appearance and self-esteem?	radio (Matrice)<br>1 No<br>2 Yes, in part<br>3 Yes, absolutely<br>4 I didn't need it<br>5 Not applicable
	186	bilan_clin_procdecis	...decision-making processes?	radio (Matrice)<br>1 No<br>2 Yes, in part<br>3 Yes, absolutely<br>4 I didn't need it<br>5 Not applicable
	187	bilan_clin_urin	...urinary and erectile dysfunction issues?	radio (Matrice)<br>1 No<br>2 Yes, in part<br>3 Yes, absolutely<br>4 I didn't need it<br>5 Not applicable
	188	bilan_org_autre_3	Have you received any other clinical information?	radio<br>0 No<br>1 Yes<br><br>Alignement personnalisé : LH
	189	paq82_v2<br>Afficher le champ UNIQUEMENT si :<br>[bilan_org_autre_3] = '1'	If yes, please specify which ones.	notes<br>Alignement personnalisé : LV
	190	bilan_quot_enf	En-tête de section : *Consequences on daily life Did you receive the information you needed regarding...*<br>...the consequences on children?	radio (Matrice)<br>1 No<br>2 Yes, in part<br>3 Yes, absolutely<br>4 I didn't need it<br>5 Not applicable

	191	bilan_quot_conj	...the consequences on conjugal life?	radio (Matrice)<br>1 No<br>2 Yes, in part<br>3 Yes, absolutely<br>4 I didn't need it<br>5 Not applicable
	192	bilan_quot_sexu	...the consequences on sexual life?	radio (Matrice)<br>1 No<br>2 Yes, in part<br>3 Yes, absolutely<br>4 I didn't need it<br>5 Not applicable
	193	bilan_quot_prof	...the consequences on professional life?	radio (Matrice)<br>1 No<br>2 Yes, in part<br>3 Yes, absolutely<br>4 I didn't need it<br>5 Not applicable
	194	bilan_quot_financ	...the financial consequences?	radio (Matrice)<br>1 No<br>2 Yes, in part<br>3 Yes, absolutely<br>4 I didn't need it<br>5 Not applicable
	195	bilan_quot_assur	...the consequences on insurance?	radio (Matrice)<br>1 No<br>2 Yes, in part<br>3 Yes, absolutely<br>4 I didn't need it<br>5 Not applicable
	196	bilan_quot_spirit	...the consequences on the spiritual life?	radio (Matrice)<br>1 No<br>2 Yes, in part<br>3 Yes, absolutely<br>4 I didn't need it<br>5 Not applicable
	197	bilan_quot_travail	...back to work?	radio (Matrice)<br>1 No<br>2 Yes, in part<br>3 Yes, absolutely<br>4 I didn't need it<br>5 Not applicable
	198	bilan_quot_retquot	...the return to daily life?	radio (Matrice)<br>1 No<br>2 Yes, in part<br>3 Yes, absolutely<br>4 I didn't need it<br>5 Not applicable

	199	bilan_quot_annon	...how to announce a diagnosis to your loved ones?	radio (Matrice)<br>1 No<br>2 Yes, in part<br>3 Yes, absolutely<br>4 I didn't need it<br>5 Not applicable
	200	bilan_quot_percsoc	...social perception (the reactions of others)?	radio (Matrice)<br>1 No<br>2 Yes, in part<br>3 Yes, absolutely<br>4 I didn't need it<br>5 Not applicable
	201	bilan_quot_strat	...strategies to live the treatments in the best conditions?	radio (Matrice)<br>1 No<br>2 Yes, in part<br>3 Yes, absolutely<br>4 I didn't need it<br>5 Not applicable
	202	bilan_quot_control	...how to regain control over the disease?	radio (Matrice)<br>1 No<br>2 Yes, in part<br>3 Yes, absolutely<br>4 I didn't need it<br>5 Not applicable
	203	bilan_org_autre_4	Did you receive any other information about the impact on daily life?	radio<br>0 No<br>1 Yes<br><br>Alignement personnalisé : LH
	204	paq97_v2<br>Afficher le champ UNIQUEMENT si :<br>[bilan_org_autre_4] = '1'	If yes, please specify which ones.	notes<br>Alignement personnalisé : LV
	205	mat_info_pers_form	En-tête de section : *The following section aims to better understand how you communicated with the team as a patient advisor. How were you informed that you had a new request for a meeting with a patient?*<br>In person, at a formal meeting	radio (Matrice)<br>1 Never<br>2 A few times<br>3 Usually<br>4 Always
	206	mat_info_pers_inform	In person, informally	radio (Matrice)<br>1 Never<br>2 A few times<br>3 Usually<br>4 Always
	207	mat_info_tel	By phone	radio (Matrice)<br>1 Never<br>2 A few times<br>3 Usually<br>4 Always
	208	mat_info_form	In writing, on a dedicated form	radio (Matrice)<br>1 Never<br>2 A few times<br>3 Usually<br>4 Always

	209	mat_info_courri	By Email	radio (Matrice)<br>1 Never<br>2 A few times<br>3 Usually<br>4 Always
	210	paq105_v2	If another means was used, please specify which one, otherwise leave the box blank.	notes<br>Alignement personnalisé : LV
	211	paq106_v2	Did you follow up with a member of the care team following your accompaniment?	radio<br>1 Yes, systematically<br>2 Yes, only if there were important elements to bring back to the team<br>3 No<br><br>Alignement personnalisé : LV
	212	bilan_suivi_acc<br>Afficher le champ UNIQUEMENT si :<br>[paq106_v2] = '1' or [paq106_v2] = '2'	If yes, how did you follow up on your accompaniments with the health care team?	descriptive<br>1 Jamais 2, Quelques fois 3, Habituellement 4, Toujours
	213	mat_suivi_pers_form<br>Afficher le champ UNIQUEMENT si :<br>[paq106_v2] = '1' or [paq106_v2] = '2'	In person, at a formal meeting	radio (Matrice)<br>1 Never<br>2 A few times<br>3 Usually<br>4 Always
	214	mat_suivi_pers_inform<br>Afficher le champ UNIQUEMENT si :<br>[paq106_v2] = '1' or [paq106_v2] = '2'	In person, informally	radio (Matrice)<br>1 Never<br>2 A few times<br>3 Usually<br>4 Always
	215	mat_suivi_tel<br>Afficher le champ UNIQUEMENT si :<br>[paq106_v2] = '1' or [paq106_v2] = '2'	By phone	radio (Matrice)<br>1 Never<br>2 A few times<br>3 Usually<br>4 Always
	216	mat_suivi_form<br>Afficher le champ UNIQUEMENT si :<br>[paq106_v2] = '1' or [paq106_v2] = '2'	In writing, on a dedicated form	radio (Matrice)<br>1 Never<br>2 A few times<br>3 Usually<br>4 Always
	217	mat_suivi_courri<br>Afficher le champ UNIQUEMENT si :<br>[paq106_v2] = '1' or [paq106_v2] = '2'	By Email	radio (Matrice)<br>1 Never<br>2 A few times<br>3 Usually<br>4 Always
	218	paq112_v2<br>Afficher le champ UNIQUEMENT si :<br>[paq106_v2] = '1' or [paq106_v2] = '2'	If another means was used, please specify which one, otherwise leave the box blank.	notes<br>Alignement personnalisé : LV
	219	mat_premier_bur	En-tête de section : *How did you make the first contact with the patients?*<br>In person, in a dedicated office in the health care facility	radio (Matrice)<br>1 Never<br>2 A few times<br>3 Usually<br>4 Always

	220	mat_premier_etab_autre	In person, at another location in the health care facility	radio (Matrice)<br>1 Never<br>2 A few times<br>3 Usually<br>4 Always
	221	mat_premier_ext	In person, outside the health care facility	radio (Matrice)<br>1 Never<br>2 A few times<br>3 Usually<br>4 Always
	222	mat_premier_tel	By phone	radio (Matrice)<br>1 Never<br>2 A few times<br>3 Usually<br>4 Always
	223	mat_premier_reacts	By secure teleconference (Reacts)	radio (Matrice)<br>1 Never<br>2 A few times<br>3 Usually<br>4 Always
	224	paq118_v2	If another means was used, please specify which one, otherwise leave the box blank.	notes<br>Alignement personnalisé : LV
	225	mat_interv_bur	En-tête de section : *How did you carry out your interventions?*<br>In person, in a dedicated office in the health care facility	radio (Matrice)<br>1 Never<br>2 A few times<br>3 Usually<br>4 Always
	226	mat_interv_etab_autre	In person, at another location in the health care facility	radio (Matrice)<br>1 Never<br>2 A few times<br>3 Usually<br>4 Always
	227	mat_interv_rdv	In person, at a medical appointment	radio (Matrice)<br>1 Never<br>2 A few times<br>3 Usually<br>4 Always
	228	mat_interv_ext	In person, outside the health care facility	radio (Matrice)<br>1 Never<br>2 A few times<br>3 Usually<br>4 Always
	229	mat_interv_tel	By phone	radio (Matrice)<br>1 Never<br>2 A few times<br>3 Usually<br>4 Always
	230	mat_interv_reacts	By secure teleconference (Reacts)	radio (Matrice)<br>1 Never<br>2 A few times<br>3 Usually<br>4 Always

	231	paq125_v2	If another means was used, please specify which one, otherwise leave the box blank.	notes<br>Alignement personnalisé : LV
	232	paq126_v2	En-tête de section : *DOCUMENTATION OF THE CONTRIBUTION OF THE PATIENT ADVISOR*<br>The purpose of this section is to document the contribution of the patient advisor in the care trajectory of the accompanied patient. In your opinion, what is the contribution of the patient advisor? Check all that apply.	checkbox<br>1 paq126_v2___1 Giving information<br>2 paq126_v2___2 Giving support<br>3 paq126_v2___3 Sharing experiences<br>4 paq126_v2___4 Facilitate communication between the health care team and the patient advisor.<br>5 paq126_v2___5 Helping to bring the patient's perspective to the health care team<br>6 paq126_v2___6 Improving understanding of information shared between patient and health care team<br>7 paq126_v2___7 Helping to remember information<br>8 paq126_v2___8 Preparing the patient for medical appointments<br>9 paq126_v2___9 Accompanying patients to medical appointments<br>10 paq126_v2___10 Helping the patient participate in decision making<br>11 paq126_v2___11 Bringing a new perspective to decision making<br>12 paq126_v2___12 Referring patients to a variety of resources<br>13 paq126_v2___13 Help develop a trusting relationship between the patient and the health care team<br>14 paq126_v2___14 Helping the patient become a partner in his or her own care<br>15 paq126_v2___15 Listening to the patient<br>16 paq126_v2___16 Other contributions, Please specify below.<br>17 paq126_v2___17 No, the patient advisor has no particular contribution to the care trajectory.<br>Alignement personnalisé : LV
	233	pacontribuautre_v2<br>Afficher le champ UNIQUEMENT si :<br>[paq126_v2(16)] = '1'	If other contributions from the patient advisor, please specify which ones.	notes<br>Alignement personnalisé : LV
	234	paq127_v2	En-tête de section : *THE IMPACTS The purpose of this section is to document the impacts (positive or negative) on yourself while you are involved as a patient advisor.*<br>Has your involvement as a patient advisor had a positive impact on you?	radio<br>1 Yes<br>2 No<br>Alignement personnalisé : LV

	235	paq127a_v2<br>Afficher le champ UNIQUEM ENT si :<br>[paq127_v2] = '1'	If yes, what are they about?	checkbox<br>1 | paq127a_v2___1 | Giving meaning to my past experience<br>2 | paq127a_v2___2 | Give back to the next one what I have received<br>3 | paq127a_v2___3 | Make me useful<br>4 | paq127a_v2___4 | Valuing me<br>5 | paq127a_v2___5 | Breaking my isolation<br>6 | paq127a_v2___6 | Developing new skills<br>7 | paq127a_v2___7 | Other, Please specify<br>Alignement personnalisé : LV
	236	paimpactpo_v2<br>Afficher le champ UNIQUEM ENT si :<br>[paq127a_v2(7)] = '1'	If other, please specify which ones.	notes<br>Alignement personnalisé : LV
	237	paq128_v2	Has your involvement as a patient advisor had a negative impact on you?	radio<br>1 | Yes<br>2 | No<br>Alignement personnalisé : LV
	238	paq128a_v2<br>Afficher le champ UNIQUEM ENT si :<br>[paq128_v2] = '1'	If yes, what are they about?	checkbox<br>1 | paq128a_v2___1 | Not feeling supported enough in my intervention<br>2 | paq128a_v2___2 | Not having had the necessary training<br>3 | paq128a_v2___3 | Not having had the necessary information<br>4 | paq128a_v2___4 | Do not fully understand my contribution<br>5 | paq128a_v2___5 | Not feeling part of the health care team<br>6 | paq128a_v2___6 | Reliving my illness<br>7 | paq128a_v2___7 | Having too many responsibilities<br>8 | paq128a_v2___8 | Having too many constraints<br>9 | paq128a_v2___9 | Not feeling up to the situation<br>10 | paq128a_v2___10 | Other, Please specify<br>Alignement personnalisé : LV
	239	paimpactne_v2<br>Afficher le champ UNIQUEM ENT si :<br>[paq128a_v2(10)] = '1'	If other, please specify which ones.	notes<br>Alignement personnalisé : LV
	240	paq_recom	In the end, do you think this type of meeting would be interesting to deploy to help more patients and caregivers?	radio<br>1 | Yes, absolutely<br>2 | Yes, but by reviewing some of the terms and conditions.<br>3 | Rather not, it doesn't really matter<br>4 | No, not at all
	241	paq_recom_precis<br>Afficher le champ UNIQUEM ENT si :<br>[paq_recom] = '2'	Please specify these terms and conditions to be reviewed.	text
	242	paq145_v2	Thank you for your participation and collaboration!	radio<br>Alignement personnalisé : LV

	243	review_of_pa_experience_with_patient_questionnaire_complete	En-tête de section : *Form Status*<br>Complete?	dropdown<br>0 Incomplete<br>1 Unverified<br>2 Complete

Formulaire : **Focus group** (focus_group) Expand

Formulaire : **PA-Debriefing report** (padebriefing_report) Expand

Formulaire : **End-of-study status** (endofstudy_status) Expand

Formulaire : **Interview** (interview) Expand

Formulaire : **Focus group general form** (focus_group_general_form) Expand
