# Supplementary material for: The patient advisor, an organizational resource as a lever for an enhanced oncology patient experience (PAROLE-onco): a longitudinal multiple case study protocol
Source: BMC Health Serv Res. 2021 Jan 4;21:10. doi: 10.1186/s12913-020-06009-4 (PMC7780212; doi:10.1186/s12913-020-06009-4)
Supplement: Supplementary file 6 — Additional file 6. Journal de bord-English-Code du dictionnaire de données - Variable name, question formulation and response options for each question in the patients advisors logbook. [file 12913_2020_6009_MOESM6_ESM.pdf]

Journal de bord-English

Guide des codes

Code du dictionnaire de données

09-10-2020 21:20

Collapse all instruments

	#	Nom de variable/champ	Étiquette de champ<br>*Note de champ*	Attributs de champ (type de champ, validation, choix, logique de branchement, calculs, etc.)
Formulaire : **Logbook** (logbook)				Collapse
	1	record_id	Record ID	text
	2	journal_instruc	Please note that for those who fill this out online, it is possible to decrease or increase the font size by clicking on the + or - at the top right.	descriptive
	3	jb_idppa	Patient advisor code :	text, Required
	4	journal_nomppa	Last name of the patient advisor:<br>*These data are identifying and will be hidden when exporting the data, only your code will appear. They will only be used in case of identification problem if the code is missing.*	text, Identifier
	5	journal_prenom	First name of the patient advisor:<br>*These data are identifying and will be hidden when exporting the data, only your code will appear. They will only be used in case of identification problem if the code is missing.*	text, Identifier
	6	jb_acc_etab	In which institution do you accompany patients?	radio<br>1 At CHUM<br>2 At CIUSSS de la Mauricie-et-du-Centre-du-Québec<br>3 At CIUSSS de l'Est-de-l'Île-de-Montréal<br>4 At CHU de Québec - Université Laval<br>5 At CISSS de Laval<br>6 At CIUSSS du Centre-Ouest-de-l'Île-de-Montréal<br>7 At CISSS de la Gaspésie<br>8 At CIUSSS du Saguenay-Lac-Saint-Jean<br>9 At CUSM
	7	jb_inf_acc	INFORMATION ON ACCOMPANIMENT	descriptive
	8	jb_id_pa	Accompanied patient code:	text
	9	jb_nom	Last name of the accompanied patient:<br>*These data are identifying and will be hidden when exporting the data, only your code will appear. They will only be used in case of identification problem if the code is missing.*	text, Identifier
	10	jb_prenom	First name of the accompanied patient:<br>*These data are identifying and will be hidden when exporting the data, only your code will appear. They will only be used in case of identification problem if the code is missing.*	text, Identifier
	11	da_jb	Date of accompaniment	text (date_dmy)
	12	jb_acc_hdebut	Start time<br>*HH:MM*	text (time)
	13	jb_acc_duree	Duration :<br>*Please write the duration in hour format (e.g. 90 minutes; write 01:30).*	text (time)
	14	jb_acc_suivi_renc	Accompaniment is a:	radio<br>1 First Meeting<br>2 Follow-up meeting at the patient's request<br>3 Follow-up meeting initiated by the patient advisor<br>4 Follow-up meeting at the request of a health care provider
	15	jb_acc_nint<br><br>Afficher le champ UNIQUEMENT si :<br>[jb_acc_suivi_renc] = '1'	Accompaniment requested by:	checkbox<br>0 jb_acc_nint___0 A health care provider<br>1 jb_acc_nint___1 The patient<br>2 jb_acc_nint___2 Other

	16	jb_demacc<br>Afficher le champ UNIQUEMENT si :<br>[jb_acc_nint(2)] = '1'	If other, please specify:	text
	17	jb_acc_numero<br>Afficher le champ UNIQUEMENT si :<br>[jb_acc_suivi_renc] = '2' or [jb_acc_suivi_renc] = '3' or [jb_acc_suivi_renc] = '4'	Meeting number:	text (number, Min. : 1, Max. : 10)
	18	jb_acc_autreint	Other person/people present at the time of the accompaniment	radio<br>0 | No<br>1 | Yes
	19	jb_acc_nautreint<br>Afficher le champ UNIQUEMENT si :<br>[jb_acc_autreint] = '1'	If yes, please specify	checkbox<br>1 | jb_acc_nautreint___1 | Spouse<br>2 | jb_acc_nautreint___2 | Child<br>3 | jb_acc_nautreint___3 | Father; Mother<br>4 | jb_acc_nautreint___4 | Friend<br>5 | jb_acc_nautreint___5 | Other
	20	jb_acc_autreint_autre<br>Afficher le champ UNIQUEMENT si :<br>[jb_acc_nautreint(5)] = '1'	If other, please specify	text
	21	jb_acc_typeacc	Type of accompaniment	radio<br>0 | In person, in a dedicated office of the institution<br>1 | In person, at another location in the institution<br>2 | In person, outside the institution<br>3 | By phone<br>4 | By secure teleconference (REACTS)<br>5 | Other
	22	jb_acc_typeacc_autre<br>Afficher le champ UNIQUEMENT si :<br>[jb_acc_typeacc] = '5'	If other, specify	text
	23	jb_acc_ca	Accompanied patient has cancer	radio<br>0 | No<br>1 | Yes
	24	jb_acc_trajca<br>Afficher le champ UNIQUEMENT si :<br>[jb_acc_ca] = '1'	Stage(s) of the care trajectory	checkbox<br>0 | jb_acc_trajca___0 | Around the announcement of the diagnosis<br>1 | jb_acc_trajca___1 | Around the screening test<br>3 | jb_acc_trajca___3 | Before starting radiotherapy<br>4 | jb_acc_trajca___4 | During radiotherapy<br>5 | jb_acc_trajca___5 | After the end of radiotherapy<br>6 | jb_acc_trajca___6 | Before starting chemotherapy<br>7 | jb_acc_trajca___7 | During chemotherapy<br>8 | jb_acc_trajca___8 | After the end of chemotherapy<br>9 | jb_acc_trajca___9 | Before surgery begins<br>10 | jb_acc_trajca___10 | After surgery<br>11 | jb_acc_trajca___11 | After the end of acute treatments<br>12 | jb_acc_trajca___12 | Other

<table>
<tr><td></td><td>25</td><td>jb_acc_trajca_autre

Afficher le champ UNIQUEM ENT si :
[jb_acc_trajca(12)] = '1'</td><td>If other, specify</td><td>text</td></tr>
<tr><td></td><td>26</td><td>jb_acc_oncogen</td><td>Accompanied patient in onco-genetics</td><td>radio<table><tr><td>0</td><td>No</td></tr><tr><td>1</td><td>Yes</td></tr></table></td></tr>
<tr><td></td><td>27</td><td>jb_acc_trajoncogen

Afficher le champ UNIQUEM ENT si :
[jb_acc_oncogen] = '1'</td><td>Step(s) in the care trajectory</td><td>checkbox<table><tr><td>0</td><td>jb_acc_trajoncogen___0</td><td>Before the genetic test</td></tr><tr><td>1</td><td>jb_acc_trajoncogen___1</td><td>Following the results of the genetic tests</td></tr><tr><td>3</td><td>jb_acc_trajoncogen___3</td><td>Before preventive surgery</td></tr><tr><td>4</td><td>jb_acc_trajoncogen___4</td><td>After preventive surgery</td></tr><tr><td>5</td><td>jb_acc_trajoncogen___5</td><td>Other</td></tr></table></td></tr>
<tr><td></td><td>28</td><td>jb_acc_trajoncogen_a

Afficher le champ UNIQUEM ENT si :
[jb_acc_trajoncogen(5)] = '1'</td><td>If other, specify</td><td>text</td></tr>
<tr><td></td><td>29</td><td>jb_acc_asp_org</td><td>En-tête de section : TOPICS COVERED DURING THE ACCOMPANIMENT

ORGANIZATIONAL ASPECTS</td><td>checkbox<table><tr><td>1</td><td>jb_acc_asp_org___1</td><td>Your role as a patient advisor</td></tr><tr><td>2</td><td>jb_acc_asp_org___2</td><td>The role of different health care professionals</td></tr><tr><td>3</td><td>jb_acc_asp_org___3</td><td>The role of external and internal organizations</td></tr><tr><td>4</td><td>jb_acc_asp_org___4</td><td>The care trajectory</td></tr><tr><td>5</td><td>jb_acc_asp_org___5</td><td>Rights as a patient (e.g., refusing treatment, asking questions)</td></tr><tr><td>6</td><td>jb_acc_asp_org___6</td><td>Where and how to get to medical appointments</td></tr><tr><td>7</td><td>jb_acc_asp_org___7</td><td>Patient financial support and transportation assistance</td></tr><tr><td>8</td><td>jb_acc_asp_org___8</td><td>The PAROLE-Onco research project</td></tr><tr><td>9</td><td>jb_acc_asp_org___9</td><td>Other</td></tr></table></td></tr>
<tr><td></td><td>30</td><td>jb_asp_org_autre

Afficher le champ UNIQUEM ENT si :
[jb_acc_asp_org(9)] = '1'</td><td>If you checked "Other", please specify what other topic was covered in this category:</td><td>text</td></tr>
</table>

<table>
<tr><td></td><td>31</td><td>jb_acc_conseq_vie_quot_fam</td><td>CONSEQUENCES ON DAILY AND FAMILY LIFE</td><td>checkbox
<table>
<tr><td>1</td><td>jb_acc_conseq_vie_quot_fam___1</td><td>On children</td></tr>
<tr><td>2</td><td>jb_acc_conseq_vie_quot_fam___2</td><td>On conjugal life</td></tr>
<tr><td>3</td><td>jb_acc_conseq_vie_quot_fam___3</td><td>About sexual life</td></tr>
<tr><td>4</td><td>jb_acc_conseq_vie_quot_fam___4</td><td>On finances</td></tr>
<tr><td>5</td><td>jb_acc_conseq_vie_quot_fam___5</td><td>On the spiritual life</td></tr>
<tr><td>6</td><td>jb_acc_conseq_vie_quot_fam___6</td><td>On professional life</td></tr>
<tr><td>7</td><td>jb_acc_conseq_vie_quot_fam___7</td><td>On insurance</td></tr>
<tr><td>8</td><td>jb_acc_conseq_vie_quot_fam___8</td><td>On how to announce a diagnosis to your family and loved ones</td></tr>
<tr><td>9</td><td>jb_acc_conseq_vie_quot_fam___9</td><td>On social perception (the reactions of others)</td></tr>
<tr><td>10</td><td>jb_acc_conseq_vie_quot_fam___10</td><td>On the return to work</td></tr>
<tr><td>11</td><td>jb_acc_conseq_vie_quot_fam___11</td><td>On the return to daily life</td></tr>
<tr><td>12</td><td>jb_acc_conseq_vie_quot_fam___12</td><td>Strategies for living the treatments in the best conditions</td></tr>
<tr><td>13</td><td>jb_acc_conseq_vie_quot_fam___13</td><td>How to regain control over the disease</td></tr>
<tr><td>14</td><td>jb_acc_conseq_vie_quot_fam___14</td><td>Other</td></tr>
</table></td></tr>
<tr><td></td><td>32</td><td>jb_viequot_autre

Afficher le champ UNIQUEMENT si : [jb_acc_conseq_vie_quot_fam(14)] = '1'</td><td>If you checked "Other", please specify what other topic was covered in this category:</td><td>text</td></tr>
</table>

<table>
<tr><td></td><td>33</td><td>jb_acc_asp_clin</td><td>CLINICAL ASPECTS</td><td>checkbox<table>
<tr><td>1</td><td>jb_acc_asp_clin___1</td><td>Announcement of cancer diagnosis / genetic predisposition by the doctor</td></tr>
<tr><td>2</td><td>jb_acc_asp_clin___2</td><td>Cancer</td></tr>
<tr><td>3</td><td>jb_acc_asp_clin___3</td><td>Oncogenetics</td></tr>
<tr><td>4</td><td>jb_acc_asp_clin___4</td><td>Genetic Tetst</td></tr>
<tr><td>5</td><td>jb_acc_asp_clin___5</td><td>Therapeutic options in the case of cancer</td></tr>
<tr><td>6</td><td>jb_acc_asp_clin___6</td><td>Reducing risk in mutation carriers</td></tr>
<tr><td>7</td><td>jb_acc_asp_clin___7</td><td>Surgical and reconstructive options</td></tr>
<tr><td>8</td><td>jb_acc_asp_clin___8</td><td>Breast implants</td></tr>
<tr><td>9</td><td>jb_acc_asp_clin___9</td><td>Hormonal issues</td></tr>
<tr><td>10</td><td>jb_acc_asp_clin___10</td><td>Reproductive issues</td></tr>
<tr><td>11</td><td>jb_acc_asp_clin___11</td><td>Impacts on Physical Appearance and Self-Esteem</td></tr>
<tr><td>12</td><td>jb_acc_asp_clin___12</td><td>Decision-making processes</td></tr>
<tr><td>13</td><td>jb_acc_asp_clin___13</td><td>Urinary and erectile dysfunction issues</td></tr>
<tr><td>14</td><td>jb_acc_asp_clin___14</td><td>Fatigue following treatments</td></tr>
<tr><td>15</td><td>jb_acc_asp_clin___15</td><td>Pain and discomfort after surgery</td></tr>
<tr><td>16</td><td>jb_acc_asp_clin___16</td><td>Pain and discomfort after treatment</td></tr>
<tr><td>17</td><td>jb_acc_asp_clin___17</td><td>Possible emotions</td></tr>
<tr><td>18</td><td>jb_acc_asp_clin___18</td><td>Stress and anxiety management</td></tr>
<tr><td>19</td><td>jb_acc_asp_clin___19</td><td>Other</td></tr>
</table></td></tr>
<tr><td></td><td>34</td><td>jb_clin_autre
Afficher le champ UNIQUEMENT si :
[jb_acc_asp_clin(19)] = '1'</td><td>If you checked "Other", please specify what other topic was covered in this category:</td><td>text</td></tr>
<tr><td></td><td>35</td><td>jb_acc_diff</td><td>En-tête de section : FOLLOW-UP OF THE INTERVENTION
Did you encounter any difficulties during the accompaniment?</td><td>radio<table><tr><td>0</td><td>No</td></tr><tr><td>1</td><td>Yes</td></tr></table></td></tr>
<tr><td></td><td>36</td><td>jb_acc_diff_lesquelles
Afficher le champ UNIQUEMENT si :
[jb_acc_diff] = '1'</td><td>If yes, which ones?</td><td>notes</td></tr>
<tr><td></td><td>37</td><td>jb_acc_quest_non_rep</td><td>Are there any questions you couldn't answer?</td><td>radio<table><tr><td>0</td><td>No</td></tr><tr><td>1</td><td>Yes</td></tr></table></td></tr>
<tr><td></td><td>38</td><td>jb_acc_ques_lesquel
Afficher le champ UNIQUEMENT si :
[jb_acc_quest_non_rep] = '1'</td><td>If yes, which ones?</td><td>notes</td></tr>
<tr><td></td><td>39</td><td>jb_acc_retour_equipe</td><td>Is a feedback to the clinical team being considered?</td><td>radio<table><tr><td>0</td><td>No</td></tr><tr><td>1</td><td>Yes</td></tr></table></td></tr>
<tr><td></td><td>40</td><td>jb_acc_retour_precis
Afficher le champ UNIQUEMENT si :
[jb_acc_retour_equipe] = '1'</td><td>Please specify your answer</td><td>notes</td></tr>
</table>

	41	jb_acc_renc_prev	Are there any other meetings scheduled with the patient?	radio<br>0 No<br>1 Yes
	42	jb_autres_info	Other relevant information:	notes<br>Alignement personnalisé : LV
	43	logbook_complete	En-tête de section : *Form Status*<br>Complete?	dropdown<br>0 Incomplete<br>1 Unverified<br>2 Complete
