# Supplementary material for: The patient advisor, an organizational resource as a lever for an enhanced oncology patient experience (PAROLE-onco): a longitudinal multiple case study protocol
Source: BMC Health Serv Res. 2021 Jan 4;21:10. doi: 10.1186/s12913-020-06009-4 (PMC7780212; doi:10.1186/s12913-020-06009-4)
Supplement: Supplementary file 7 — Additional file 7. PAROLE-Onco-Professionnels-English-Code du dictionnaire de données - Variable name, question formulation and response options for each question in the professionals questionnaires. [file 12913_2020_6009_MOESM7_ESM.pdf]

PARAOLE-Onco-Professionnels-English

Guide des codes

Code du dictionnaire de données

09-10-2020 21:17

Collapse all instruments

	#	Nom de variable/champ	Étiquette de champ<br>*Note de champ*	Attributs de champ (type de champ, validation, choix, logique de branchement, calculs, etc.)
Formulaire : **Participant identification form** (participant_identification_form) Expand				
Formulaire : **PAROLE-Onco - Questionnaire T0- Clinical team** (paroleonco_questionnaire_t0_clinical_team) Collapse				

	19	pro_bidon1	En-tête de section : *INFORMATION AND CONSENT*<br>You are invited to participate in the PAROLE-Onco research project as a professional. However, before giving your consent to participate in this project, please take the time to carefully read, understand and consider the following information. In addition, we invite you to ask any questions you may have to the researcher in charge of the project or other members assigned to this research project and to ask them to explain any words or information that are not clear.<br><br>The objective of this research project is to evaluate the impact of integrating patient advisors into cancer care teams. These patient advisors are patients who have already experienced episodes of cancer care and services and who use their experience with other patients who are experiencing a similar episode.<br><br>Your participation in this project consists of completing a 15-minute online questionnaire about your perception of the introduction of patient advisors, collaborative practices and innovative culture.<br><br>You will not derive any personal benefit from your participation in this research project. However, the results obtained will contribute to the advancement of practices and scientific knowledge in this field.<br><br>All information collected will remain confidential within the limits of the law. This information includes your name, your contact information, the name of your institution and the title of your position. You will only be identified by a code number. The code key linking your name to your research file will be kept by the responsible researcher. Research data may be published or discussed scientifically without you being identified. The data collected will be kept for 10 years after the end of the study and then destroyed.<br><br>Your participation in this research is voluntary. You are therefore free to refuse to participate. You can also stop answering the questionnaire at any time by closing your web browser window. Your answers will then be automatically deleted.<br><br>If you have any questions or comments about this study, please do not hesitate to contact us by email at marie-pascale.pomey@umontreal.ca. If you have any questions about your rights as a participant in this research project, or if you have any complaints or comments, you can contact the CHUM's local service quality and complaints commissioner at 514 890-8484 or your institution's service quality and complaints commissioner, if different from the CHUM.<br><br>The CHUM's research ethics committee has approved the project and will ensure that the project is monitored by the participating institutions in Quebec's health and social services network.<br><br>I have read the above information and understand its content. As such, my participation in this questionnaire is voluntary and I consent to the use of my answers for the purposes of this research project. Completion of this questionnaire will be considered as my consent to participate in the study and to the use of the data collected in this questionnaire for the research purposes described in this form.<br><br>Finally, please note that for those who complete the questionnaire online, it is possible to decrease or increase the font size by clicking on the + or - in the upper right-hand corner.	checkbox<br>Alignement personnalisé : LV
	20	pro_bidon6	En-tête de section :	descriptive

	21	pro_bidon3	The statements below address various themes related to collaborative practice. Please indicate your level of agreement with each statement based on your experience with the health care team you work with in oncology or oncogenetics. This is the team where patient advisors will be/are involved.	descriptive
	22	pro_respect	En-tête de section : *COLLABORATIVE PRACTICES: GENERAL RELATIONSHIPS*<br>Respect among team members improves with our ability to work together.	radio (Matrice)<br>0 Strongly disagree<br>1 Mostly disagree<br>2 Somewhat disagree<br>3 Neither agree nor disagree<br>4 Somewhat agree<br>5 Mostly agree<br>6 Strongly agree
	23	pro_bienetre	Team members care about one another's personal well being.	radio (Matrice)<br>0 Strongly disagree<br>1 Mostly disagree<br>2 Somewhat disagree<br>3 Neither agree nor disagree<br>4 Somewhat agree<br>5 Mostly agree<br>6 Strongly agree
	24	pro_actso	Socializing together enhances team work effectiveness.	radio (Matrice)<br>0 Strongly disagree<br>1 Mostly disagree<br>2 Somewhat disagree<br>3 Neither agree nor disagree<br>4 Somewhat agree<br>5 Mostly agree<br>6 Strongly agree
	25	pro_plaisant	It is enjoyable to work with other team members.	radio (Matrice)<br>0 Strongly disagree<br>1 Mostly disagree<br>2 Somewhat disagree<br>3 Neither agree nor disagree<br>4 Somewhat agree<br>5 Mostly agree<br>6 Strongly agree
	26	pro_expertise	Team members respect each other's roles and expertise.	radio (Matrice)<br>0 Strongly disagree<br>1 Mostly disagree<br>2 Somewhat disagree<br>3 Neither agree nor disagree<br>4 Somewhat agree<br>5 Mostly agree<br>6 Strongly agree
	27	pro_enthou	Working collaboratively keeps most team members enthusiastic and interested in their job.	radio (Matrice)<br>0 Strongly disagree<br>1 Mostly disagree<br>2 Somewhat disagree<br>3 Neither agree nor disagree<br>4 Somewhat agree<br>5 Mostly agree<br>6 Strongly agree

	28	pro_confiance	Team members trust each other's work and contributions related to patient care.	radio (Matrice)<br>0 Strongly disagree<br>1 Mostly disagree<br>2 Somewhat disagree<br>3 Neither agree nor disagree<br>4 Somewhat agree<br>5 Mostly agree<br>6 Strongly agree
	29	pro_habil	Our team's level of respect for each other enhances our ability to work together.	radio (Matrice)<br>0 Strongly disagree<br>1 Mostly disagree<br>2 Somewhat disagree<br>3 Neither agree nor disagree<br>4 Somewhat agree<br>5 Mostly agree<br>6 Strongly agree
	30	pro_harmonie	En-tête de section : *COLLABORATIVE PRACTICES: EVALUATION OF PRACTICES*<br>We regularly exchange on the harmonization of care practices during our team meetings and discussions.	radio (Matrice)<br>0 Strongly disagree<br>1 Mostly disagree<br>2 Somewhat disagree<br>3 Neither agree nor disagree<br>4 Somewhat agree<br>5 Mostly agree<br>6 Strongly agree
	31	pro_pairs	Our team has a process for peer review.	radio (Matrice)<br>0 Strongly disagree<br>1 Mostly disagree<br>2 Somewhat disagree<br>3 Neither agree nor disagree<br>4 Somewhat agree<br>5 Mostly agree<br>6 Strongly agree
	32	pro_reconnais	En-tête de section : *COLLABORATIVE PRACTICES: GENERAL ROLE, RESPONSIBILITIES, AUTONOMY*<br>Team members acknowledge the aspects of care where members of my profession have more skills and expertise.	radio (Matrice)<br>0 Strongly disagree<br>1 Mostly disagree<br>2 Somewhat disagree<br>3 Neither agree nor disagree<br>4 Somewhat agree<br>5 Mostly agree<br>6 Strongly agree
	33	pro_medresp	Physicians assume the ultimate responsibility for team decisions and outcomes.	radio (Matrice)<br>0 Strongly disagree<br>1 Mostly disagree<br>2 Somewhat disagree<br>3 Neither agree nor disagree<br>4 Somewhat agree<br>5 Mostly agree<br>6 Strongly agree

	34	pro_nego	Team members negotiate the role they want to take in developing and implementing the patient care plan.	radio (Matrice)<br>0 Strongly disagree<br>1 Mostly disagree<br>2 Somewhat disagree<br>3 Neither agree nor disagree<br>4 Somewhat agree<br>5 Mostly agree<br>6 Strongly agree
	35	pro_reptrav	Team members are held accountable for their work.	radio (Matrice)<br>0 Strongly disagree<br>1 Mostly disagree<br>2 Somewhat disagree<br>3 Neither agree nor disagree<br>4 Somewhat agree<br>5 Mostly agree<br>6 Strongly agree
	36	pro_implique	It is clear who is responsible for aspects of the patient care plan.	radio (Matrice)<br>0 Strongly disagree<br>1 Mostly disagree<br>2 Somewhat disagree<br>3 Neither agree nor disagree<br>4 Somewhat agree<br>5 Mostly agree<br>6 Strongly agree
	37	pro_opinion	Physicians usually ask other team members for opinions about patient care.	radio (Matrice)<br>0 Strongly disagree<br>1 Mostly disagree<br>2 Somewhat disagree<br>3 Neither agree nor disagree<br>4 Somewhat agree<br>5 Mostly agree<br>6 Strongly agree
	38	pro_defint	Team members feel comfortable advocating for the patient.	radio (Matrice)<br>0 Strongly disagree<br>1 Mostly disagree<br>2 Somewhat disagree<br>3 Neither agree nor disagree<br>4 Somewhat agree<br>5 Mostly agree<br>6 Strongly agree
	39	pro_respdec	Each team member shares accountability for team decisions and outcomes.	radio (Matrice)<br>0 Strongly disagree<br>1 Mostly disagree<br>2 Somewhat disagree<br>3 Neither agree nor disagree<br>4 Somewhat agree<br>5 Mostly agree<br>6 Strongly agree

	40	pro_comexpert	Team members have the responsibility to communicate and provide their expertise in an assertive manner.	radio (Matrice)<br>0 Strongly disagree<br>1 Mostly disagree<br>2 Somewhat disagree<br>3 Neither agree nor disagree<br>4 Somewhat agree<br>5 Mostly agree<br>6 Strongly agree
	41	pro_autono	Team members feel limited in the degree of autonomy in patient care that they can assume.	radio (Matrice)<br>0 Strongly disagree<br>1 Mostly disagree<br>2 Somewhat disagree<br>3 Neither agree nor disagree<br>4 Somewhat agree<br>5 Mostly agree<br>6 Strongly agree
	42	pro_preocc	Patients concerns are addressed effectively through regular team meetings and discussion	radio (Matrice)<br>0 Strongly disagree<br>1 Mostly disagree<br>2 Somewhat disagree<br>3 Neither agree nor disagree<br>4 Somewhat agree<br>5 Mostly agree<br>6 Strongly agree
	43	pro_stratcom	Our team has developed effective communication strategies to share patient treatment goals and outcomes of care.	radio (Matrice)<br>0 Strongly disagree<br>1 Mostly disagree<br>2 Somewhat disagree<br>3 Neither agree nor disagree<br>4 Somewhat agree<br>5 Mostly agree<br>6 Strongly agree
	44	pro_rapid	Relevant information relating to changes in patient status or care plan is reported to the appropriate team member in a timely manner.	radio (Matrice)<br>0 Strongly disagree<br>1 Mostly disagree<br>2 Somewhat disagree<br>3 Neither agree nor disagree<br>4 Somewhat agree<br>5 Mostly agree<br>6 Strongly agree
	45	pro_juste	I trust the accuracy of information reported among team members.	radio (Matrice)<br>0 Strongly disagree<br>1 Mostly disagree<br>2 Somewhat disagree<br>3 Neither agree nor disagree<br>4 Somewhat agree<br>5 Mostly agree<br>6 Strongly agree

	46	pro_ouvert	Our team meetings provide an open, comfortable, safe place to discuss concerns.	radio (Matrice)<br>0 Strongly disagree<br>1 Mostly disagree<br>2 Somewhat disagree<br>3 Neither agree nor disagree<br>4 Somewhat agree<br>5 Mostly agree<br>6 Strongly agree
	47	pro_dosmed	The patient health record is used effectively by all team members as a communication tool.	radio (Matrice)<br>0 Strongly disagree<br>1 Mostly disagree<br>2 Somewhat disagree<br>3 Neither agree nor disagree<br>4 Somewhat agree<br>5 Mostly agree<br>6 Strongly agree
	48	pro_liberagir	En-tête de section : *INNOVATIVE CULTURE*<br>In my workplace, people have a great deal of freedom to act and make the necessary changes.	radio (Matrice)<br>0 Strongly disagree<br>1 Mostly disagree<br>2 Somewhat disagree<br>3 Neither agree nor disagree<br>4 Somewhat agree<br>5 Mostly agree<br>6 Strongly agree
	49	pro_encou	People on our team encourage each other to try new things.	radio (Matrice)<br>0 Strongly disagree<br>1 Mostly disagree<br>2 Somewhat disagree<br>3 Neither agree nor disagree<br>4 Somewhat agree<br>5 Mostly agree<br>6 Strongly agree
	50	pro_direncou	Management encourages people to try new things.	radio (Matrice)<br>0 Strongly disagree<br>1 Mostly disagree<br>2 Somewhat disagree<br>3 Neither agree nor disagree<br>4 Somewhat agree<br>5 Mostly agree<br>6 Strongly agree
	51	pro_flex	Our team is flexible and adapts quickly to new opportunities.	radio (Matrice)<br>0 Strongly disagree<br>1 Mostly disagree<br>2 Somewhat disagree<br>3 Neither agree nor disagree<br>4 Somewhat agree<br>5 Mostly agree<br>6 Strongly agree

	52	pro_decision	When a decision is made, it is implemented quickly.	radio (Matrice)<br>0 Strongly disagree<br>1 Mostly disagree<br>2 Somewhat disagree<br>3 Neither agree nor disagree<br>4 Somewhat agree<br>5 Mostly agree<br>6 Strongly agree
	53	pro_autonome	Our team is autonomous enough to implement new ideas without the authorization of supervisors.	radio (Matrice)<br>0 Strongly disagree<br>1 Mostly disagree<br>2 Somewhat disagree<br>3 Neither agree nor disagree<br>4 Somewhat agree<br>5 Mostly agree<br>6 Strongly agree
	54	pro_organise	In this organization, we are always looking for new ways to organize our work to provide better care.	radio (Matrice)<br>0 Strongly disagree<br>1 Mostly disagree<br>2 Somewhat disagree<br>3 Neither agree nor disagree<br>4 Somewhat agree<br>5 Mostly agree<br>6 Strongly agree
	55	pro_bidon4	En-tête de section : *PERCEPTION OF INTEGRATION OF PATIENT ADVISORS (PAs)*<br>In your institution, patient advisors who have experienced significant events related to cancer or a genetic predisposition to cancer are willing to share their experience with other patients who are experiencing similar situations. In this context, we would like to know your opinion.	descriptive
	56	pro_fampa	Are you familiar with the concept of a patient advisor?	radio<br>0 No, I've never heard of it<br>1 I have heard vaguely about it<br>2 Yes, I am familiar with this notion<br><br>Alignement personnalisé : LV
	57	pro_parlerpa<br>Afficher le champ UNIQUEM ENT si :<br>[pro_fampa] = '2' or [pro_fam pa] = '1'	Where did you hear about it? Please select all that apply:	checkbox<br>0 pro_parlerpa___0 Via internet<br>1 pro_parlerpa___1 Via the website of the establishment<br>2 pro_parlerpa___2 Via the written press of the establishment<br>3 pro_parlerpa___3 Via the university<br>4 pro_parlerpa___4 Via my head of department<br>5 pro_parlerpa___5 Via a colleague<br>6 pro_parlerpa___6 Via a patient<br>7 pro_parlerpa___7 Via the media<br>8 pro_parlerpa___8 Via a conference or literature<br>9 pro_parlerpa___9 Other<br><br>Alignement personnalisé : LV
	58	pro_parleraautre<br>Afficher le champ UNIQUEM ENT si :<br>[pro_parlerpa(9)] = '1'	If other, please specify:	text

	59	pro_utilpa	Do you see the value of former patients (called patient advisors) being able to share their experience with others in similar situations?	radio<br>0 No<br>1 Yes<br><br>Alignement personnalisé : LV
	60	pro_integre	Do you think these patient advisors could be integrated into your team?	radio<br>0 Yes<br>1 No<br>2 Maybe<br><br>Alignement personnalisé : LV
	61	pro_obstacles	What might be the barrier(s) to integrating these patient advisors (PAs) into your clinic? Please select all that apply:	checkbox<br>1 pro_obstacles___1 Resistance to change<br>2 pro_obstacles___2 Lack of time<br>3 pro_obstacles___3 Work overload<br>4 pro_obstacles___4 Lack of physical space<br>5 pro_obstacles___5 Fear of inadequate PA's intervention<br>6 pro_obstacles___6 Fear that PAs address emotional issues that may be difficult to manage<br>7 pro_obstacles___7 Confusion of roles<br>8 pro_obstacles___8 Patient distrust<br>9 pro_obstacles___9 Patients' sense of intrusiveness<br>10 pro_obstacles___10 Confronting different perceptions of the PA and the physician<br>11 pro_obstacles___11 No relevance<br>12 pro_obstacles___12 No value added to already available resources<br>13 pro_obstacles___13 There are no barriers to the integration of patient advisors.<br>14 pro_obstacles___14 Don't know<br>15 pro_obstacles___15 Other<br><br>Alignement personnalisé : LV
	62	pro_obstaclesautre<br>Afficher le champ UNIQUEMENT si :<br>[pro_obstacles(13)] = '1'	If other, please specify:	text<br>Alignement personnalisé : LV
	63	pro_bidon5	Please check the box that best corresponds to your opinion.	descriptive
	64	pro_parplan	I believe in the importance of patient and family involvement in planning and decision-making for one's own care.	radio (Matrice)<br>0 Strongly Disagree<br>1 Disagree<br>2 Neither agree nor disagree<br>3 Agree<br>4 Strongly agree
	65	pro_pvtous	I believe that the views and opinions of patients and their families are just as valid as those of staff and clinicians in planning and making decisions about a patient's care plan.	radio (Matrice)<br>0 Strongly Disagree<br>1 Disagree<br>2 Neither agree nor disagree<br>3 Agree<br>4 Strongly agree

	66	pro_utileepi	I believe that involving the PA in the planning and decision-making of a patient's care plan can be helpful.	radio (Matrice)<br>0 Strongly Disagree<br>1 Disagree<br>2 Neither agree nor disagree<br>3 Agree<br>4 Strongly agree
	67	pro_contribu	I believe that PAs can contribute at the same level as health care providers in the provision of services.	radio (Matrice)<br>0 Strongly Disagree<br>1 Disagree<br>2 Neither agree nor disagree<br>3 Agree<br>4 Strongly agree
	68	pro_initiative	I am comfortable asking for PAs to be invited to participate in initiatives.	radio (Matrice)<br>0 Strongly disagree<br>1 Disagree<br>2 Neither agree nor disagree<br>3 Agree<br>4 Strongly agree
	69	pro_chargetrav	I consider that the participation of PAs does not increase my workload.	radio (Matrice)<br>0 Strongly disagree<br>1 Disagree<br>2 Neither agree nor disagree<br>3 Agree<br>4 Strongly agree
	70	pro_resppro	I wonder about my professional responsibility towards the participation of PAs.	radio (Matrice)<br>0 Strongly disagree<br>1 Disagree<br>2 Neither agree nor disagree<br>3 Agree<br>4 Strongly agree
	71	pro_enjeu	I wonder about the legal issues related to the participation of PAs in my organization.	radio (Matrice)<br>0 Strongly disagree<br>1 Disagree<br>2 Neither agree nor disagree<br>3 Agree<br>4 Strongly agree
	72	pro_perspect	I believe that PAs bring an interesting perspective to improve the service offer.	radio (Matrice)<br>0 Strongly disagree<br>1 Disagree<br>2 Neither agree nor disagree<br>3 Agree<br>4 Strongly agree
	73	pro_membre	I consider PAs to be full-fledged members of the health care team.	radio (Matrice)<br>0 Strongly disagree<br>1 Disagree<br>2 Neither agree nor disagree<br>3 Agree<br>4 Strongly agree

	74	pro_favo	My organization promotes the participation of PAs in my daily work.	radio (Matrice)<br>0 Strongly disagree<br>1 Disagree<br>2 Neither agree nor disagree<br>3 Agree<br>4 Strongly agree
	75	pro_formcont	Have you had continuing education about the partnership of care?	radio<br>0 No<br>1 Yes<br><br>Alignement personnalisé : LV
	76	nul	Thank you for contributing to the improvement of patient well-being.<br>Thank you for completing this questionnaire.<br><br>Please click on the check mark to access the socio-demographic questionnaire which will complete the questionnaire.	descriptive
	77	pro_bidonreft1	En-tête de section : *REFERENCES*<br><br>Collaborative practice assessment tool (CPAT) © mars 2009 Owned by the Office of Interprofessional Education and Practice. Queen's University.<br><br>Group Innovation Inventory, Caldwell DF, O'Reilly CA (2003)	descriptive
	78	pro_depot	For the administration: submission of the paper questionnaire	file
	79	paroleonco_questionnaire_t0_clinical_team_complete	En-tête de section : *Form Status*<br>Complete?	dropdown<br>0 Incomplete<br>1 Unverified<br>2 Complete

Formulaire : **PAROLE-Onco - Socio-demographic questionnaire** (paroleonco_sociodemographic_questionnaire) Collapse

	80	pro_region	In which administrative region do you predominantly practice your profession?	radio<br>0 Bas-Saint-Laurent (01)<br>1 Saguenay-Lac-Saint-Jean (02)<br>2 Capitale-Nationale (03)<br>3 Mauricie (04)<br>4 Estrie (05)<br>5 Montréal (06)<br>6 Outaouais (07)<br>7 Abitibi-Témiscamingue (08)<br>8 Côte-Nord (09)<br>9 Nord-du-Québec (10)<br>10 Gaspésie-Îles-de-la-Madeleine (11)<br>11 Chaudière-Appalaches (12)<br>12 Laval (13)<br>13 Lanaudière (14)<br>14 Laurentides (15)<br>15 Montérégie (16)<br>16 Centre-du-Québec (17)<br><br>Alignement personnalisé : LV

	81	recrupro	From which institution were you recruited for this project?	radio<br>1 At CHUM<br>2 At CIUSSS de la Mauricie-et-du-Centre-du-Québec<br>3 At CIUSSS de l'Est-de-l'Île-de-Montréal<br>4 At CHU de Québec-Université Laval<br>5 At CISSS de Laval<br>6 At CIUSSS du Centre-Ouest-de-l'Île-de-Montréal<br>7 At CISSS de la Gaspésie<br>8 At CIUSSS du Saguenay-Lac-Saint-Jean<br>9 At CUSM<br><br>Alignement personnalisé : LV<br>Annotation de champ: @p1000notes{"":"Établissement de recrutement"}
	82	pro_occup	What is your profession?	radio<br>0 Family physician, general practitioner<br>1 Hemato-oncologist<br>3 Radio-oncologist<br>4 Medical oncologist<br>5 General oncological surgeon<br>6 Nurse<br>7 Auxiliary nurse<br>8 Clinical Nurse<br>9 Pivot nurse in oncology<br>11 Pharmacist<br>12 Psychologist<br>13 Social worker<br>14 Community worker<br>15 Technologist (radio-oncology)<br>16 Manager<br>17 Other<br><br>Alignement personnalisé : LV<br>Annotation de champ: @p1000notes{"":"Occupation "}
	83	pro_occupautre<br>Afficher le champ UNIQUEMENT si :<br>[pro_occup] = '17'	If Other, specify :	text<br>Annotation de champ: @p1000notes{"":"Autre occupation"}
	84	pro_sexe	What is your gender?	radio<br>0 Male<br>1 Female<br><br>Alignement personnalisé : LV<br>Annotation de champ: @p1000notes{"":"Sexe"}
	85	pro_age	What is your age group?	radio<br>0 24 years old and under<br>1 25 - 34 years old<br>2 35 - 44 years old<br>3 45 - 54 years old<br>4 55 - 64 years old<br>5 65 - 74 years old<br>6 75 years old and over<br><br>Alignement personnalisé : LV

	86	pro_scol	What is the highest level of education you have completed?	radio<br>0 Secondary or professional (DES/DEP)<br>1 College (Non-University Certificate/DEC)<br>2 University<br>3 Other<br><br>Alignement personnalisé : LV
	87	pro_scolautre<br><br>Afficher le champ UNIQUEMENT si :<br>[pro_scol] = '3'	If other, please specify:	text<br>Alignement personnalisé : LV
	88	pro_postean	How long have you been in this position?	radio<br>0 Less than 1 year<br>1 Between 1 and less than 3 years old<br>2 Between 3 and less than 5 years old<br>3 Between 5 and less than 10 years old<br>4 10 years and over<br><br>Alignement personnalisé : LV
	89	pro_posteanprecise<br><br>Afficher le champ UNIQUEMENT si :<br>[pro_postean] = '4'	If 10 years and over, please specify :<br>*X years*	text (number)<br>Alignement personnalisé : LV
	90	pro_milieu	In which environment(s) do you work?	checkbox<br>0 pro_milieu___0 Family Medicine Group (FMG)<br>1 pro_milieu___1 Outpatient Cancer Clinic<br>2 pro_milieu___2 CLSC<br>3 pro_milieu___3 Inpatient Unit<br>4 pro_milieu___4 Other<br><br>Alignement personnalisé : LV
	91	pro_milieuautre<br><br>Afficher le champ UNIQUEMENT si :<br>[pro_milieu(4)] = '1'	If other, please specify :	text<br>Alignement personnalisé : LV
	92	pro_milieumed<br><br>Afficher le champ UNIQUEMENT si :<br>[pro_milieu(0)] = '1'	If you work in a FMG, please specify :	checkbox<br>0 pro_milieumed___0 Family Medicine Group (FMG)<br>1 pro_milieumed___1 University Family Medicine Group (FMG-U)<br>2 pro_milieumed___2 Family Medicine Network Group (FMG-R or Superclinic)<br><br>Alignement personnalisé : LV
	93	pro_bidon9	Answer the following questions based on an average that you feel is representative.<br>*X heure(s)*	descriptive
	94	pro_heuresem_qso	How many hours do you work per week?<br>*X hours*	text (number)<br>Alignement personnalisé : LV
	95	pro_heureonco_qso	How many hours per week do you work more specifically for oncology or oncogenetics patients?<br>(If the question does not apply, please indicate 0)<br>*X hour(s) (including clinical and management activities, projects, etc.)*	text (number)<br>Alignement personnalisé : LV
	96	pro_perjour_qso	How many patients do you see per week, regardless of the type of patient?<br>(If the question does not apply, please indicate 0)<br>*X patient(s)*	text (number)<br>Alignement personnalisé : LV
	97	pro_personco_qso	How many patients do you see in a week in an oncology or oncogenetic clinic?<br>(If the question does not apply, please indicate 0)<br>*X patient(s)*	text<br>Alignement personnalisé : LV
	98	pro_depotqsocio	For administration: submission of the paper questionnaire.	file<br>Alignement personnalisé : LV

	99	paroleonco_sociodemographic_questionnaire_complete	En-tête de section : *Form Status*<br>Complete?	dropdown<br>0 Incomplete<br>1 Unverified<br>2 Complete

Formulaire : **PAROLE-Onco- Questionnaire T2- Clinical Team** (paroleonco_questionnaire_t2_clinical_team) Expand

Formulaire : **Focus group** (focus_group) Expand

Formulaire : **Interview** (interview) Expand

Formulaire : **Pilot interview** (pilot_interview) Expand

Formulaire : **Focus group general form** (focus_group_general_form) Expand

Formulaire : **Status - End of study** (status_end_of_study) Expand
